# A diazirine's central carbon is sp^2^-hybridized, facilitating conjugation to dye molecules†

**DOI:** 10.1039/d4sc06427e

**Published:** 2024-12-02

**Authors:** Lorenzo Michelini, Tanya Slaney, Seerat Virk, Estefanía Rafic, L. Charlie Qie, Klara Corejova, Mathieu L. Lepage, Stefania F. Musolino, Allen G. Oliver, Roberto Etchenique, W. David Hong, Gino A. DiLabio, Jeremy E. Wulff

**Affiliations:** a Department of Chemistry, University of Victoria Victoria BC V8W 3V6 Canada wulff@uvic.ca; b Department of Chemistry, Sapienza University of Rome P.le A. Moro 5 00185 Rome Italy; c Departamento de Química Inorgánica, Analítica y Química Física, INQUIMAE, Facultad de Ciencias Exactas y Naturales. Universidad de Buenos Aires CONICET. Pabellón 2, Ciudad Universitaria C1428EHA Buenos Aires Argentina; d Department of Chemistry, University of Liverpool Liverpool L69 7ZD UK davidhwq@liverpool.ac.uk; e Fundamental and Applied Heterochemistry Laboratory (UMR CNRS 5069), Paul Sabatier University 31062 Toulouse Cedex 9 France; f XLYNX Materials, Inc. Victoria BC V8P 5C2 Canada; g Molecular Structure Facility, Department of Chemistry and Biochemistry, University of Notre Dame Notre Dame IN 46556 USA; h Department of Chemistry, University of British Columbia Kelowna BC V1V-1V7 Canada gino.dilabio@ubc.ca; i Centre for Advanced Materials and Related Technology (CAMTEC), University of Victoria Victoria BC V8W 2Y2 Canada

## Abstract

Diazirines are versatile carbene precursors that are extensively used in biological target identification experiments. However, their photo-activation wavelength (*ca.* 365 nm) precludes their use in living organisms. Here we show that a reconceptualization of the diazirine hybridization state leads to conjugation of the diazirine motif to longer-wavelength chromophores. In a model diazirine–fluorene conjugate, we are able to achieve direct activation (and subsequent C–H insertion) with >450 nm light for the first time. Two-photon activation using near-IR light is also achieved, suggesting the possibility to prepare new diazirine probes for conducting target identification experiments in deep tissue.

## Abstract 2

A fluorene-conjugated diazirine is described, for which two-photon activation is demonstrated for the first time.

## Graphical abstract

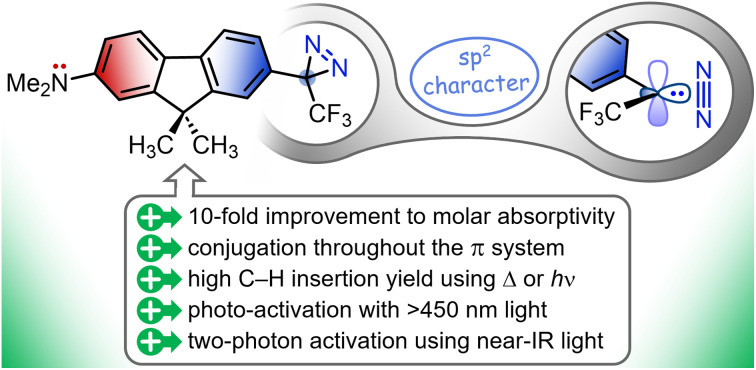

## Introduction

**Fig. 1:**
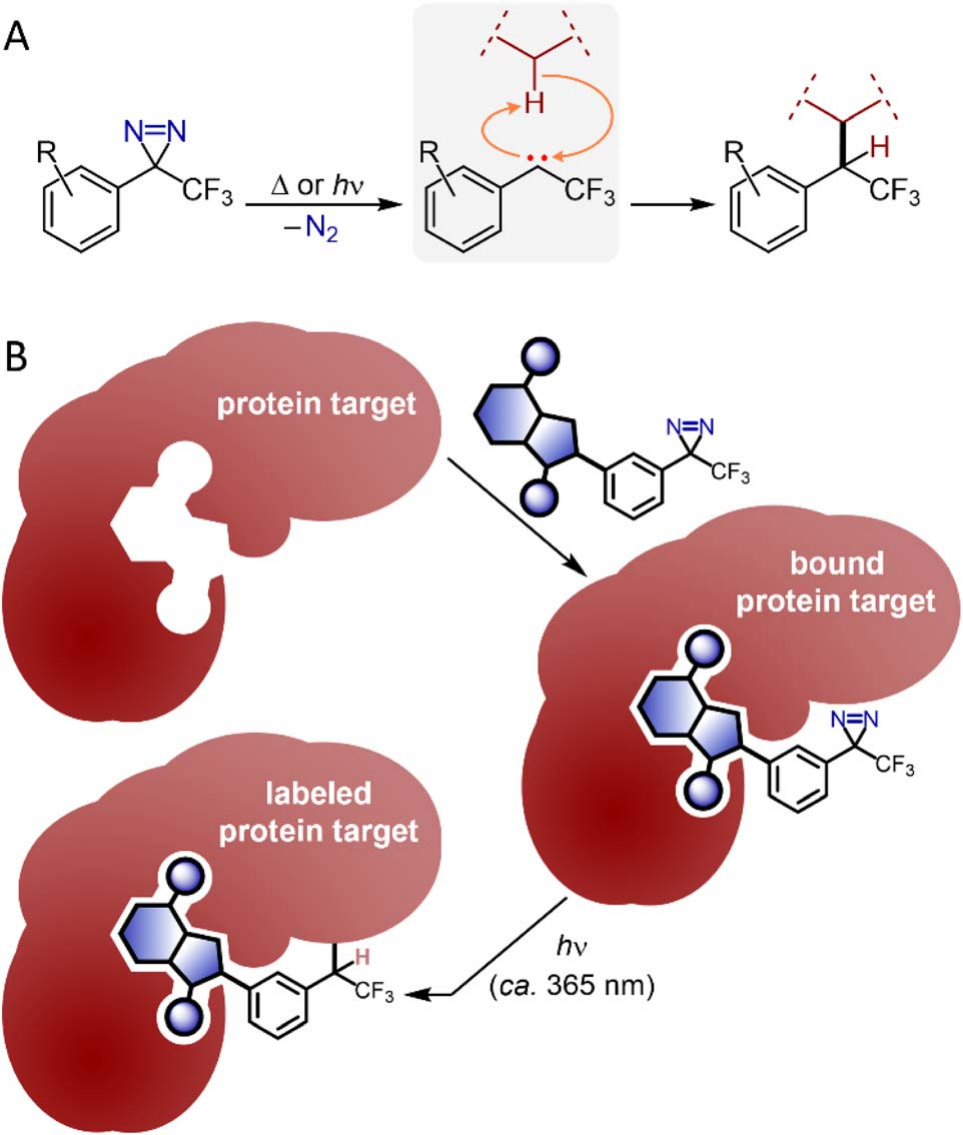
Overview of diazirine reactivity and utility in chemical biology. (A) Photochemical or thermal activation of diazirine reagents reveals reactive carbenes that can engage in X–H insertions to nearby substrate molecules. (B) Photoaffinity labelling of target proteins with diazirine-enabled small molecules allows for identification of macromolecular binding partners.

Diazirine groups—strained three-membered rings containing two doubly bound nitrogen atoms—release nitrogen gas upon thermal or photochemical excitation, revealing reactive carbenes that can undergo C–H, O–H, or N–H insertion to nearby substrates (Fig. 1A). First popularized by Brunner for use in biological target identification,^1^ trifluoromethyl aryl diazirines have been used extensively to search for protein-binding partners for bioactive small molecules (Fig. 1B).^2–4^ Thanks to their small size (relative to alternative photoactivatable groups like benzophenones), excellent stability (in the absence of bright light), lack of toxicity,^5^ and desirable activation wavelength (*ca.* 365 nm; considerably longer than that required for activation of benzophenones or aryl azides), trifluoromethyl aryl diazirines have emerged as the ‘gold standard’ for target identification experiments. Reagents developed on the trifluoromethyl aryl diazirine scaffold are also now frequently employed for proximity mapping of T-cell protein environments,^6^ and for the functionalization^7^ and crosslinking^8–10^ of low-functionality aliphatic polymers.

Activation of diazirine motifs with 365 nm light allows for the convenient labelling of biological targets within cell lysates. However, UV light damages cells, and is not able to penetrate deeply into living tissue, precluding the use of diazirines for identifying protein-binding partners in living organisms. In an effort to find photoactivatable reagents that can be used for target identification in a broader range of contexts, Dai and Yang recently examined the use of a diazoalkyl group (*i.e.* the linear isomer of a cyclic diazirine) that was conjugated to a coumarin dye.^11^ The resulting conjugate could be activated to the corresponding carbene using visible light (430–490 nm), and could even be activated using near-IR light (800 nm) through a two-photon activation process.^11,12^ However, linear diazoalkyl groups react with carboxylic acids present in biomolecules, and are also prone to engaging in dipolar cycloaddition reactions.^13^ This broader reactivity profile makes them useful in organic synthesis (especially when coupled with transition-metal catalysis^14^), but would be a significant limitation for biological applications. Indeed, linear diazo compounds have never been extensively used for biological target identification. Azido-conjugates of dye molecules—which can be photo-activated to the corresponding nitrene—are better known,^15^ but aryl nitrenes are prone to undesirable aza-Büchner ring expansions (which ‘uses up’ the reactive nitrene), and azides have shorter activation wavelengths than diazirines.

Given the preference in chemical biology for trifluoromethyl aryl diazirine reagents, the development of diazirine conjugates that can be activated using longer wavelengths of light would be highly desirable. However, it is unclear whether such an outcome is even possible through directly linking a diazirine group to a suitable chromophore. Certainly the way that diazirines are commonly *drawn* in the chemical literature, with four sigma bonds connected to the central carbon atom, suggests that—unlike azides, benzophenones, or linear diazo groups—cyclic diazirines are linked through a saturated atom. As such, one might not expect to see viable electronic conjugation from the chromophore to the diazirine LUMO, which needs to be populated in order to facilitate loss of N_2_ and generation of a carbene. This expected lack of conjugation, born from an expectation of sp^3^ (or possibly sp^5^) hybridization at the central carbon of a diazirine, is likely the reason that no direct chromophore conjugates of trifluoromethyl diazirines have yet been reported.§§The MacMillan and Steele groups have both shown that trifluoromethyl aryl diazirines can be activated *via* energy transfer from iridium dyes that absorb visible light;^6,33^ this approach, however, requires that the sensitizer and diazirine can come into close contact with one another—a requirement that would not be achievable for most biological targeting applications, without adding significant molecular weight to the probe. To the best of our knowledge, there are no previous examples of direct trifluoromethyl diazirine–chromophore conjugates, where photochemical excitation of the chromophore leads to activation of the diazirine motif.^34,35^

In this communication, we present a combination of computational, spectroscopic, and X-ray crystallographic evidence in support of the contention that the central carbon atom in a diazirine group is best described as being sp^2^-hybridized. While we acknowledge that orbital hybridization is merely a conceptual model, and need not have any bearing on the atoms themselves, the observation of p-orbital contribution from this carbon suggests the ready incorporation of diazirines onto light-harvesting π systems. As a test-case for our hypothesis of diazirine–chromophore conjugation, we use a trifluoromethyl diazirine group as the electron-withdrawing motif in a classic ‘push–pull’ fluorene dye. We find that the resulting conjugate still functions as an effective chromophore, indicating that the diazirine group can replace a traditional conjugated electron-withdrawing group. Moreover, the diazirine–fluorene conjugate could be activated with 460 nm light to form a carbene capable of inserting into stable, unactivated C–H bonds. Finally, a two-photon activation using near-IR light is also demonstrated for the first time on a trifluoromethyl aryl diazirine.^16^

Taken together, these data suggest a significantly expanded role for diazirines in target identification experiments requiring longer-wavelength light sources.

## Results and discussion

**Fig. 2:**
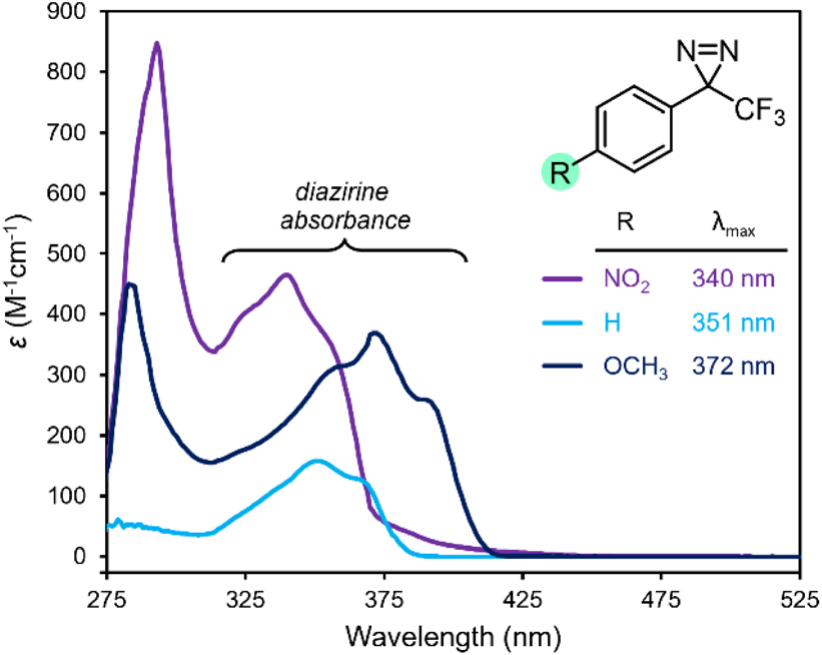
Changes in UV/Vis absorption spectra with varying substitution on the aromatic ring of a trifluoromethyl aryl diazirine. All data were acquired using 5 mM samples in *n*-hexane. Replotted from raw data published in ref. 17.

In 2021, Musolino and co-workers published a comprehensive structure–function survey of the trifluoromethyl aryl diazirine motif, varying the electronics on the aromatic ring.^17^ As shown in Fig. 2, the absorbances associated with the diazirine group shifted to longer wavelengths when electron-donating groups were appended to the *para*-position of the aryl ring, while electron-withdrawing groups had the opposite effect. Superficially, this result might seem puzzling: if the central carbon of the diazirine motif is sp^3^-hybridized (as is implied by the typical structural representation of a diazirine, with four sigma bonds extending from the central carbon), then why should functionality present at the *para*-position of the aromatic ring affect the electronics of the diazirine so dramatically?

**Fig. 3:**
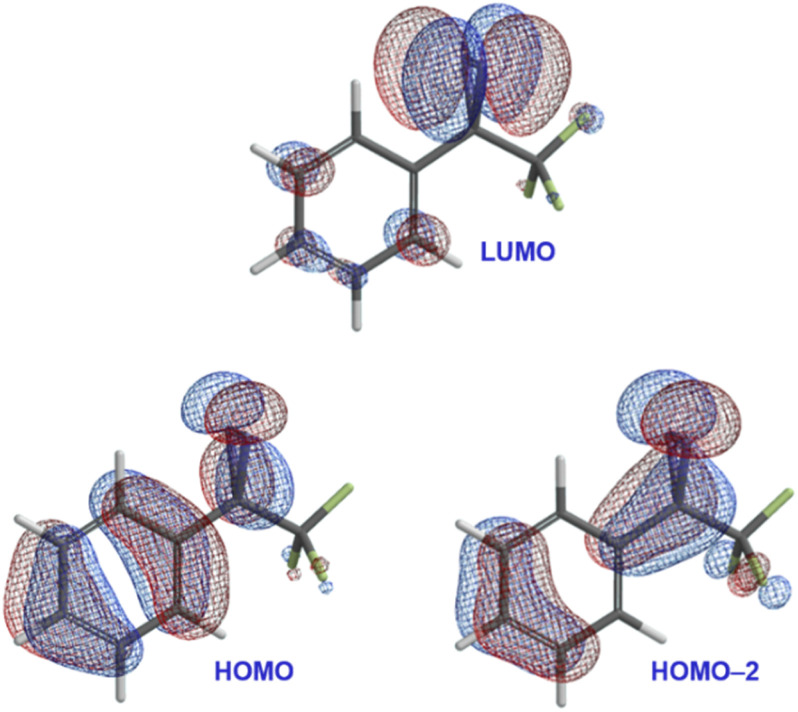
Selected calculated frontier molecular orbitals (M06-2X/6-311+G(2df,2p)) for trifluoromethyl phenyl diazirine, suggesting conjugation from the aromatic ring onto the central carbon atom of the diazirine group.

To address this question, we carried out DFT calculations on trifluoromethyl phenyl diazirine. We found that the lowest unoccupied molecular orbital (LUMO) was centred mostly on the diazirine group (Fig. 3), while both the highest occupied molecular orbital (HOMO) and the HOMO−2 extended across the aromatic ring and onto the diazirine unit. The HOMO−1 (not shown) was localized to the benzene ring.

In the HOMO and HOMO−2 molecular orbitals shown in Fig. 3, the contribution from the diazirine carbon is due nearly 100% to an atomic p-orbital. A Mulliken population analysis (see Table S1 in the ESI†) reveals essentially zero s-character from this carbon atom to either molecular orbital. This suggested that the diazirine group could be considered to be ‘conjugated’ to the benzene ring. Of course, in order for conjugation to be established, the diazirine needs to be coplanar to the aromatic ring (*i.e.* the dihedral angle between the CF_3_ group, the diazirine carbon, the *ipso* carbon on the benzene ring, and the neighbouring *ortho* carbon needs to be close to zero). Achieving this conformation would require that the molecule pay a steric penalty (resulting from interactions between the CF_3_ group and the *ortho* C–H), which must be overcome by the electronic stabilization resulting from orbital delocalization.

Recognizing, then, that the conformational preferences within the system would provide insight into the degree of conjugation, we calculated the relative energies of a series of different conformations for trifluoromethyl phenyl diazirine, varying the dihedral angle in 10° steps. As a control, we also calculated the energies of the corresponding diaziridine: the saturated analogue of a diazirine, for which no conjugation would be expected.

**Fig. 4:**
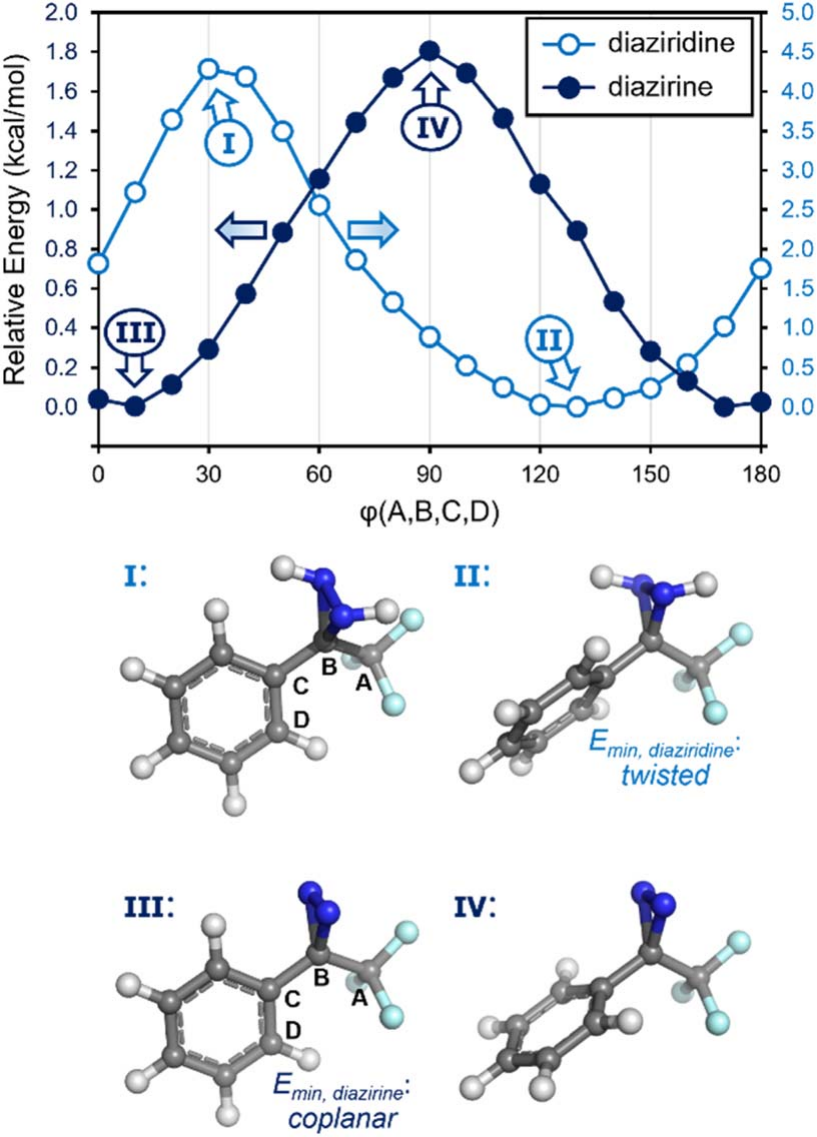
Calculated conformational preferences (M06-2X/6-311+G(2df,2p)) for trifluoromethyl phenyl diazirine, and for the corresponding diaziridine.

As shown in Fig. 4, the diazirine adopts a nearly coplanar conformation in its ground state, in which the *φ*_CF_3_–C_*diazirine*_–C_*ipso*_–C_*ortho*__ dihedral angle is approximately 10°. The coplanar conformer was favoured over the corresponding 90° structure by *ca.* 1.8 kcal mol^−1^. By contrast, the saturated diaziridine molecule preferentially adopted a twisted conformation (*φ* = 130°), in which the steric repulsions within the molecule were minimized.

**Fig. 5:**
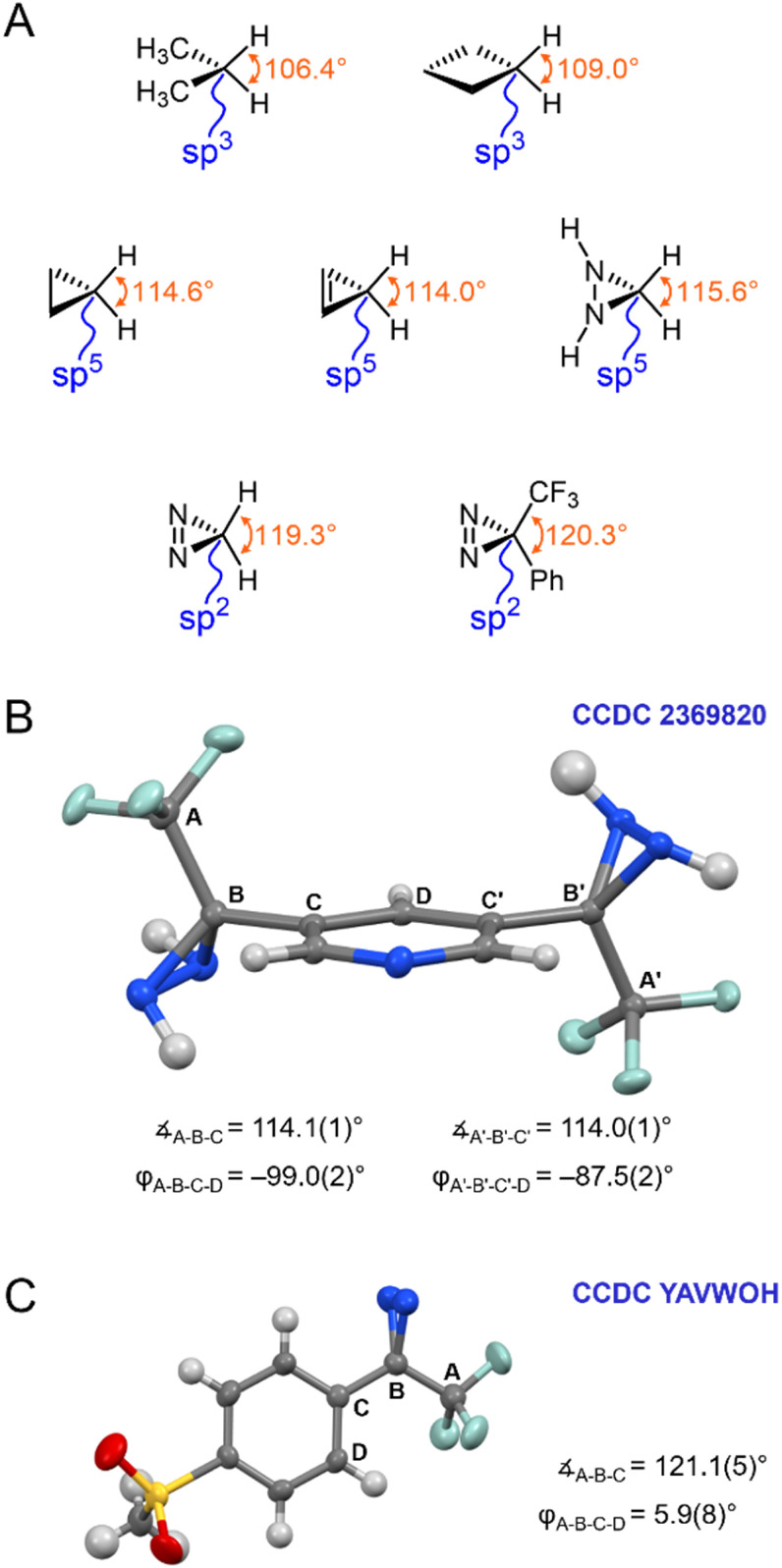
Structural properties of diazirines and comparator molecules. (A) Calculated bond angles (optimized using M06-2X/6-311+G(2df,2p)) for representative sp^3^ and sp^5^ alkanes and alkenes, together with calculated structures for diazirines and diaziridines. (B) X-ray structure for 3,5-bis(3-(trifluoromethyl)diaziridin-3-yl)pyridine (CCDC 2369820). (C) X-ray structure for 3-(4-(methylsulfonyl)phenyl)-3-(trifluoromethyl)-3*H*-diazirine (CCDC YAVWOH).^20^ Thermal ellipsoids are shown at 50% probability.

The calculated properties illustrated in Fig. 3 and 4 strongly imply the existence of conjugation between the diazirine group and the attached aromatic ring. As such, they suggest that the central carbon of the diazirine might best be described as being sp^2^-hybridized, rather than being assigned the sp^3^-hybridization state that is typically ascribed to saturated systems, or the sp^5^-hybridization that is sometimes used to explain the properties of cyclopropanes.^18^ Recognizing that hybridization state strongly influences bond angle (or, put another way, that bond angle constitutes one of the central pieces of data upon which the theoretical construction of hybridization state is based), we calculated the optimized geometry for the parent diazirine (H_2_CN_2_) and diaziridine (H_2_CN_2_H_2_) molecules, as well as for trifluoromethyl phenyl diazirine. A series of control molecules were also calculated for comparison. As shown in Fig. 5A, the control compounds all had the expected bond angles. For example, cyclobutane (in which each carbon atom would be assigned the sp^3^-hybridization state) had calculated H–C–H bond angles of 109°, while cyclopropane (the prototypical sp^5^-hybridized structure) had calculated H–C–H bond angles of 115°. Both values are consistent with literature reports.^19^ As expected, the saturated diaziridine molecule also had an H–C–H bond angle of close to 115°, suggesting that it too can be described as sp^5^-hybridized, similar to cyclopropane or cyclopropene. Remarkably, however, the calculated H–C–H bond angle for the parent diazirine molecule—as well as the calculated CF_3_–C–aryl bond angle for trifluoromethyl phenyl diazirine—were both approximately 120°. This observation strongly supports the description of the central carbon as being sp^2^-hybridized.

Seeking additional structural evidence, we searched the Cambridge Structural Database for relevant diazirines and diaziridines. A 1993 report by the Barton group described the first example of a trifluoromethyl aryl diazirine X-ray structure (Fig. 5C).^20^ Although not remarked upon in the publication associated with the structure, the molecule adopted a coplanar geometry (*φ*_CF_3_–C_*diazirine*_–C_*ipso*_–C_*ortho*__ < 10°), supporting conjugation between the aryl group and the diazirine. Perhaps most strikingly, the CF_3_–C–aryl bond angle was approximately 120°, in perfect agreement with an sp^2^-hybridization state for the central carbon atom.¶¶A total of six X-ray structures for trifluoromethyl aryl diazirines have been reported to date. Five of these have the diazirine positioned approximately coplanar to the aromatic ring, and have CF_3_–C–aryl bond angles of *ca*. 121°.^20,24,36,37^ The sixth structure has the diazirine unit twisted with respect to the aryl group (*φ*_CF_3_–C_*diazirine*_–C_*ipso*_–C_*ortho*__ = 84°), perhaps due to crystal packing forces, and this molecule displays a smaller CF_3_–C–aryl bond angle of 118°.^38^ These data provide further support for the hypothesis that the degree of orbital conjugation and the bond angle around the diazirine central carbon are coupled to one another. See Table S2 in the ESI for a complete list of structures, together with relevant bond and dihedral angles.

We did not find any suitable trifluoromethyl aryl diaziridines in the Cambridge Database for comparison,||||There are six trifluoromethyl aryl diaziridines for which structures have been reported previously in the Cambridge Structural Database,^20,39,40^ but each of these has additional substituents present on the nitrogen atoms. Regardless, for each of these molecules the diazirine group adopted a twisted conformation (*φ*_CF_3_–C_*diazirine*_–C_*ipso*_–C_*ortho*__ > 60°) and the CF_3_–C–aryl bond angle was approximately 114°. See Table S3 in the ESI for a complete list of structures, together with relevant bond and dihedral angles. and so perused our own laboratory's library of diaziridine molecules from previous projects. We were successful in obtaining X-ray quality crystals of 3,5-bis(3-(trifluoromethyl)diaziridin-3-yl)pyridine, a molecule that was first synthesized by our group in 2019.^8^ The solved structure (CCDC 2369820) is shown in Fig. 5B. As anticipated based on our earlier calculations, this control molecule (for which conjugation would not be expected, due to the saturated N–N bonds) adopted a staggered conformation, and had CF_3_–C–aryl bond angles of approximately 115°—consistent with a cyclopropane-like sp^5^-hybridization state at the central carbon atom.

The dramatic differences in conformational preference and bond angles observed for diaziridines and diazirines clearly support a change in hybridization state of the central carbon, as the N–N single bond is changed to an N=N double bond. This is distinct from what happens in moving from cyclopropane to cyclopropene (see Fig. 5A), where the bond angles around the non-doubly bonded carbon remain relatively constant. We contend based upon the data shown here that the diazirine motif (at least when embedded within a trifluoromethyl aryl diazirine functional group) is best described as being connected through an sp^2^-hybridized carbon atom, which thereby supports conjugation to an attached aromatic ring.

If the above statement is accurate, then it should be possible to append trifluoromethyl diazirine groups to better chromophores than the simple substituted benzene rings summarized in Fig. 2. Doing so may allow the diazirine to be activated with longer wavelengths of light, and might even allow for two-photon activation using red or near-IR light. To test this hypothesis, we sought to pursue a synthesis of a representative molecule in which a diazirine was attached to a suitable dye molecule. In principle, several different dyes could have been chosen for this purpose, but we opted to use a 9,9-dimethylfluorene scaffold as our chromophore, since we felt that it constituted an ideal platform for testing our central hypothesis around the degree of conjugation between the diazirine group and the attached aromatic π system.

Dimethylfluorene dyes are known to be effective chromophores when they bear an electron-donating group (typically a disubstituted amine) on one aromatic ring, and a conjugated electron-withdrawing group on the opposite ring.^21,22^ The absorbance spectrum of any given fluorene conjugate thus provides a convenient readout of the degree of electronic communication within the system.

**Scheme 1:**
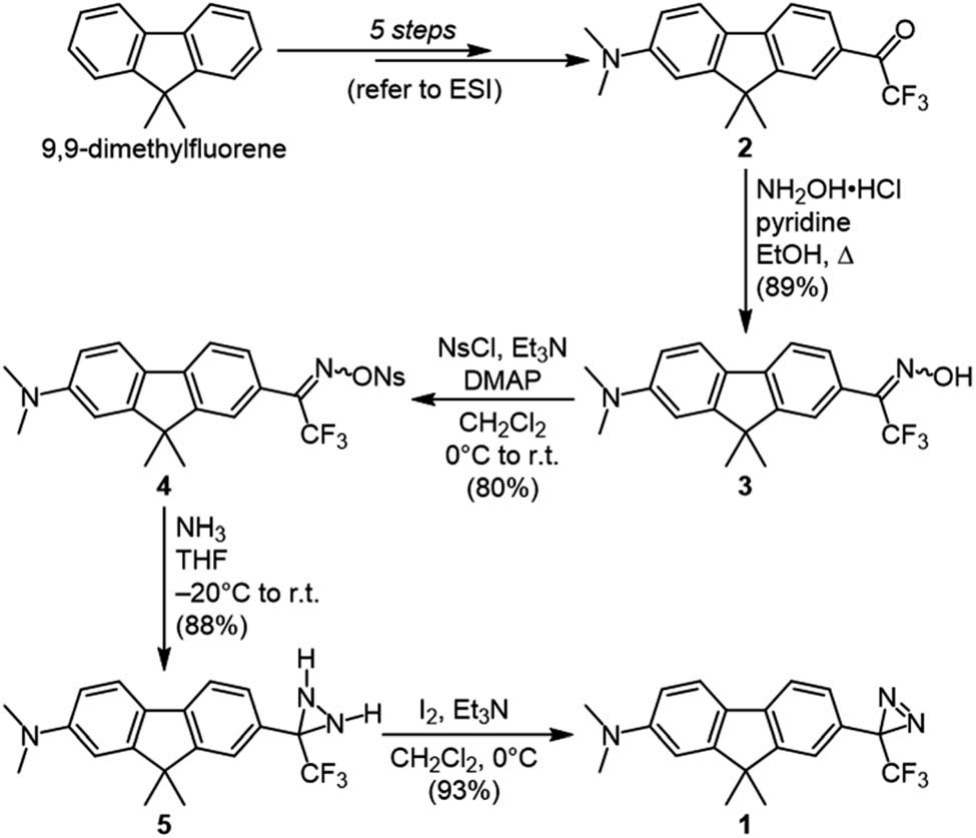
Synthesis of fluorene–diazirine conjugate 1.

Our target diazirine (1) was synthesized as shown in Scheme 1. Beginning from the corresponding trifluoromethyl ketone (2, available in 5 steps from commercial 9,9-dimethylfluorene), we generated oxime 3 through the addition of hydroxylamine hydrochloride. The oxime was then activated as the nosylate (4),^23^ and gaseous ammonia was added to generate the diaziridine intermediate (5). This in turn could be efficiently oxidized to the desired diazirine using molecular iodine in the presence of triethylamine.^8^

**Fig. 6:**
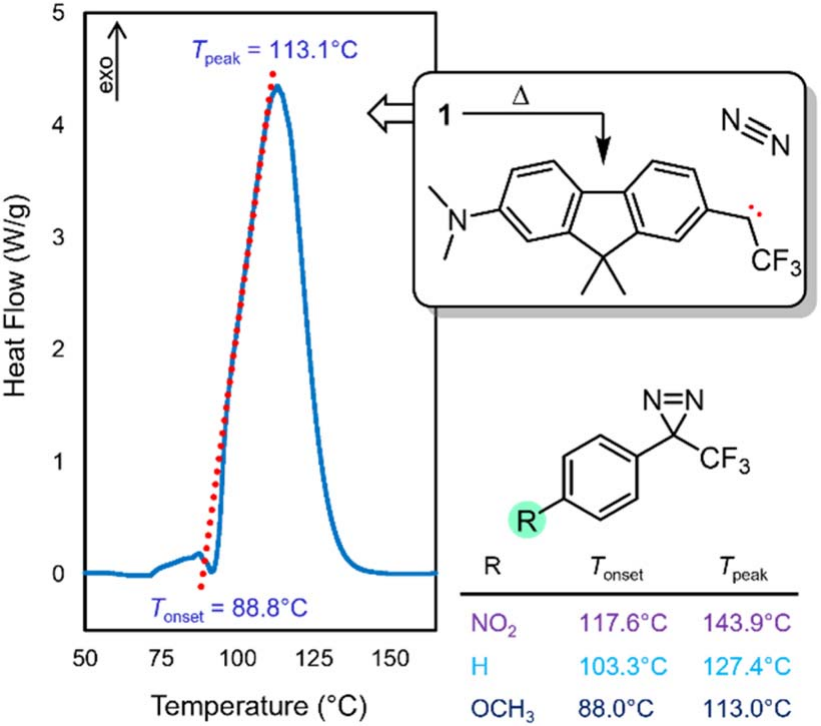
DSC data for compound 1, showing the diazirine activation exotherm. *T*_peak_ corresponds to the temperature at which the maximum heat flow is observed. *T*_onset_ is determined experimentally from extrapolation of the tangent of the upward slope observed in the DSC experiment (red dotted line), to the fitted baseline of the plot. Inset data taken from ref. 17 show the decrease in activation temperature with increasing electron density in the aromatic ring.

**Fig. 7:**
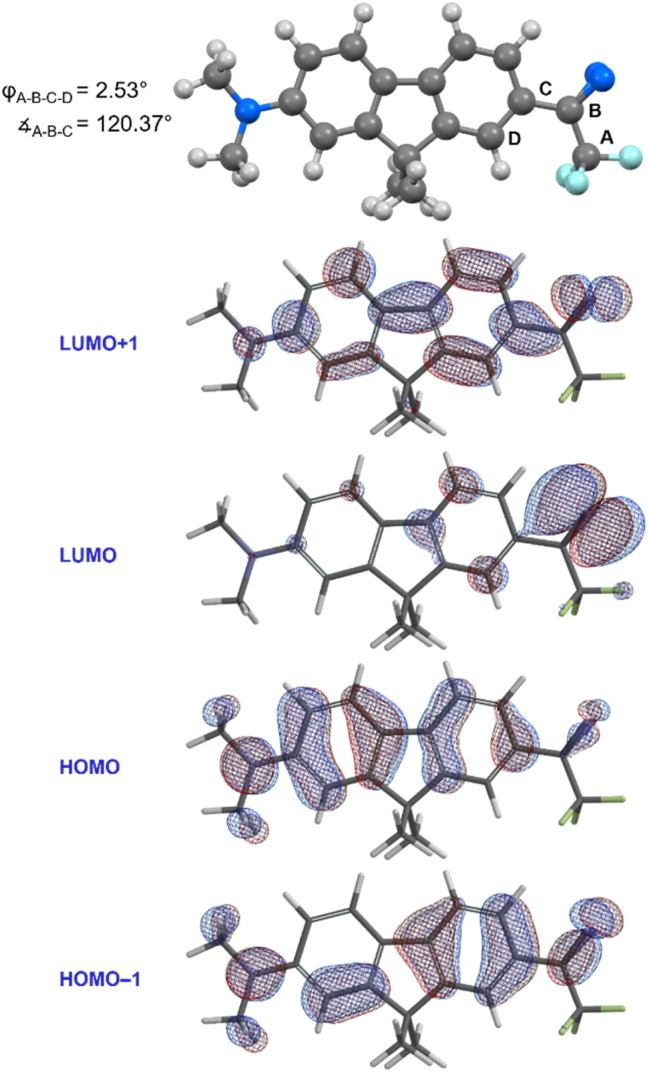
Geometry-optimized structure and frontier molecular orbitals calculated for compound 1 (M06-2X/6-311+G(2df,2p)).

With compound 1 in hand, we studied its optical properties by UV/Vis spectroscopy and its thermal properties by differential scanning calorimetry (DSC). In the DSC experiment, we were pleased to note an onset temperature (*T*_onset_) of 89 °C for the diazirine activation exotherm, and a peak temperature (*T*_peak_) of 113 °C (Fig. 6). The values are significantly lower than those for the unsubstituted trifluoromethyl phenyl diazirine (*T*_onset_ = 103 °C; *T*_peak_ = 127 °C), and are in fact nearly identical to those for the *p*-methoxyphenyl analogue thereof (*T*_onset_ = 88 °C; *T*_peak_ = 113 °C).^17^ We previously showed that the *T*_onset_ and *T*_peak_ values shift with the degree of electron-richness in the aromatic ring (see inset data in Fig. 6).^17^ The activation temperatures that characterize the diazirine activation exotherm for compound 1 thus provided the first experimental verification that the properties of the diazirine were perturbed by the presence of the electron-donating dimethylamino group on the opposite ring of the dimethylfluorene scaffold (although the influence of this strong electron-donating group is no doubt attenuated by being positioned two rings away). As a control, we also synthesized an analogue (1′), which lacked the dimethylamino substituent across the fluorene π system from the diazirine (refer to the ESI for details†). As expected, control molecule 1′ had very similar thermal properties (*T*_onset_ = 102 °C; *T*_peak_ = 125 °C) to those of unsubstituted trifluoromethyl phenyl diazirine. The >10 °C reduction in diazirine activation temperature with the introduction of the remote dimethylamino group suggests a significant degree of electronic communication across the molecule. This suggestion was further supported by DFT calculations, which revealed molecular orbitals extending from the electron-donating dimethylamino substituent all the way through the diazirine motif (Fig. 7). As with the calculations performed for the unsubstituted trifluoromethyl phenyl diazirine in Fig. 3, the geometry-optimized structure for compound 1 had the diazirine motif nearly coplanar to the aromatic ring (*φ*_CF_3_–C_*diazirine*_–C_*ipso*_–C_*ortho*__ = 2.5°), and the calculated CF_3_–C–aryl bond angle was 120.4°. These data once again suggested an sp^2^-hybridization state for the central carbon atom of the diazirine, as did the fact that in the only frontier orbital to which the diazirine carbon made a significant contribution (the HOMO−1), the C*_diazirine_* atomic orbital contributing to the MO is clearly a p-orbital.

**Fig. 8:**
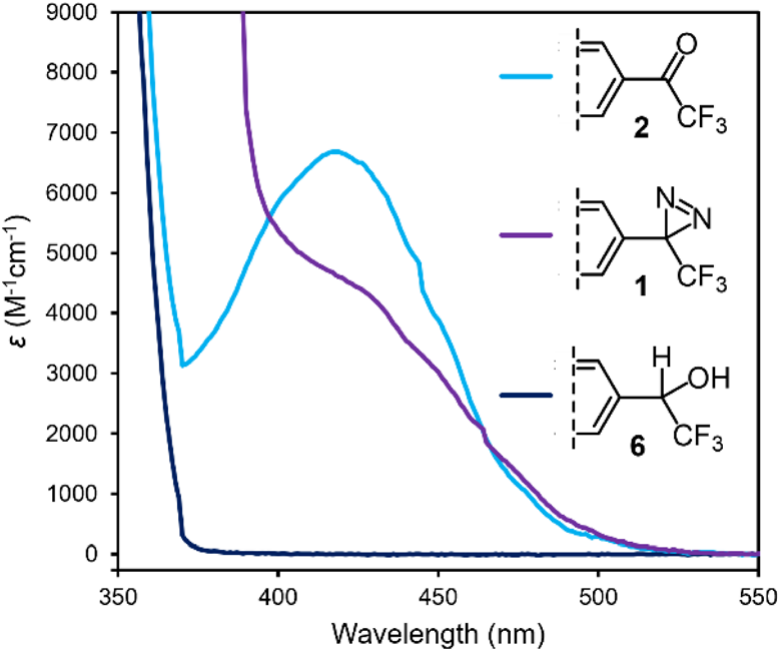
UV/Vis spectrum for diazirine 1, together with positive and negative control compounds 2 and 6. All data were acquired using 0.05 mM samples in MeOH. Refer to the ESI† for UV/Vis data for compounds 1′, 5, and 7, and for UV/Vis spectra collected in cyclohexane.

As described above, the 9,9-dimethylfluorene scaffold functions as an effective chromophore when the electron-donating group on one of the aromatic rings is paired with a conjugated electron-withdrawing group on the opposite ring. The absorbance spectrum for any given fluorene molecule can thus provide a qualitative assessment of the degree of conjugation for a substituent. To probe the electronic communication in diazirine 1, we compared its UV/Vis spectrum to that of trifluoromethyl ketone 2 (a positive control for conjugation) and benzyl alcohol 6 (a negative control for conjugation, available by hydride reduction of the ketone). As expected, ketone 2 behaved as an efficient ‘push–pull’ fluorene chromophore, resulting in a strong absorbance band centred at 418 nm in methanol, and extending beyond 475 nm (light blue trace in Fig. 8). By contrast, negative control 6 (in which the sp^3^-hybridized benzylic centre prohibits conjugation from the electron-withdrawing trifluoromethyl group into the π system), displayed no absorbance beyond 375 nm (dark blue trace in Fig. 8). Similarly, 1′ (in which the electron-donating Me_2_N substituent is absent) exhibited minimal absorbance in the visible region. The UV/Vis spectrum for diazirine 1 (purple trace in Fig. 8) had more in common with that of 2 than with 1′ or 6, displaying a strong absorbance between 400 and 475 nm. Although less strongly absorbing than the corresponding trifluoromethyl ketone in this region, the nonzero absorbance provided a strong indication of effective conjugation between the diazirine and the π system of the dimethylfluorene.

**Fig. 9:**
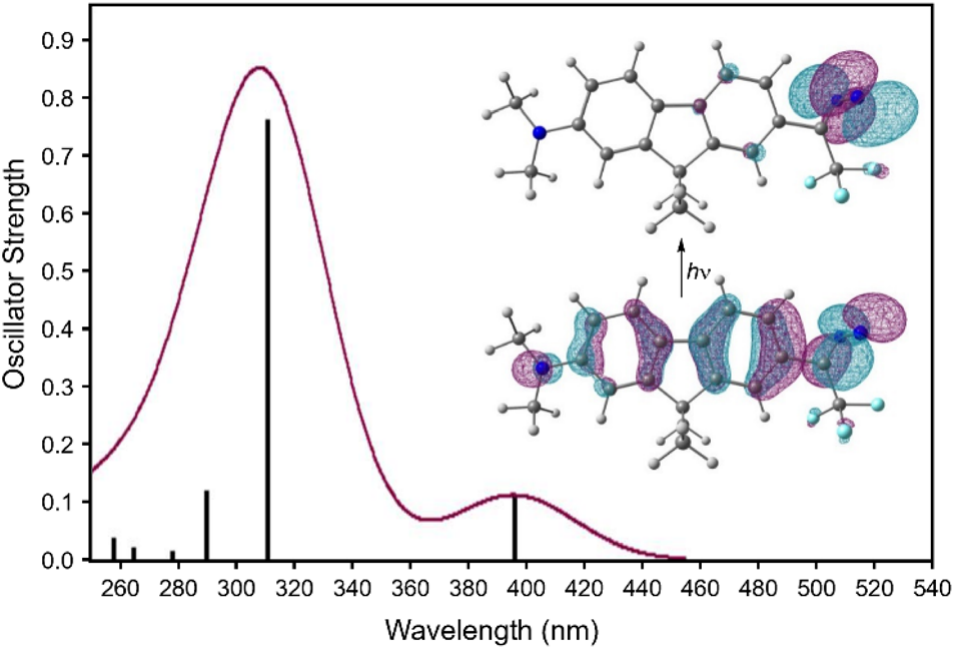
TD-DFT calculated UV/Vis spectrum for diazirine 1. Geometry optimization and frequency calculations: M06-2X/6-31+G(d,p); TD-DFT: CAM-B3LYP/aug-cc-pVTZ with implicit methanol solvation. Inset figure shows natural transition orbitals^30^ involved in the low-energy excitation.

The visible-wavelength absorbance observed for compound 1 suggested the possibility that the diazirine might be activated to the corresponding carbene using visible light. This hypothesis was further supported by TD-DFT calculations (Fig. 9) in which the long-wavelength absorbance band resulted from electronic excitation from the extended π system into the diazirine-centred LUMO.

Spurred on by these findings, we investigated the activation of diazirine 1 with 460 nm light. The reaction was carried out in the presence of cyclohexane, which we and others have used as a convenient benchmarking substrate to compare the efficacy of various diazirine reagents.^8–10,23–29^ The unactivated C–H bonds in cyclohexane present a challenging target for carbene insertion, and the yield of the C–H insertion adduct reports on the degree of singlet stabilization for the intermediate carbene.^17,23^ Diazirine molecules that lead to ground-state triplet carbenes (*e.g.* the unsubstituted trifluoromethyl phenyl diazirine) give low yields of the desired C–H insertion product (typically <20%) due to competing reaction with adventitious oxygen (also a ground-state triplet). By contrast, diazirine molecules that beget ground-state singlet carbenes can lead to very high yields of the corresponding cyclohexane C–H insertion product. The champion carbene precursors in this regard are *p*-alkoxyphenyl trifluoromethyl diazirines, which are able to add to cyclohexane in >90% isolated yield, regardless of whether thermal or photochemical activation conditions are used.^17,23^

**Scheme 2:**
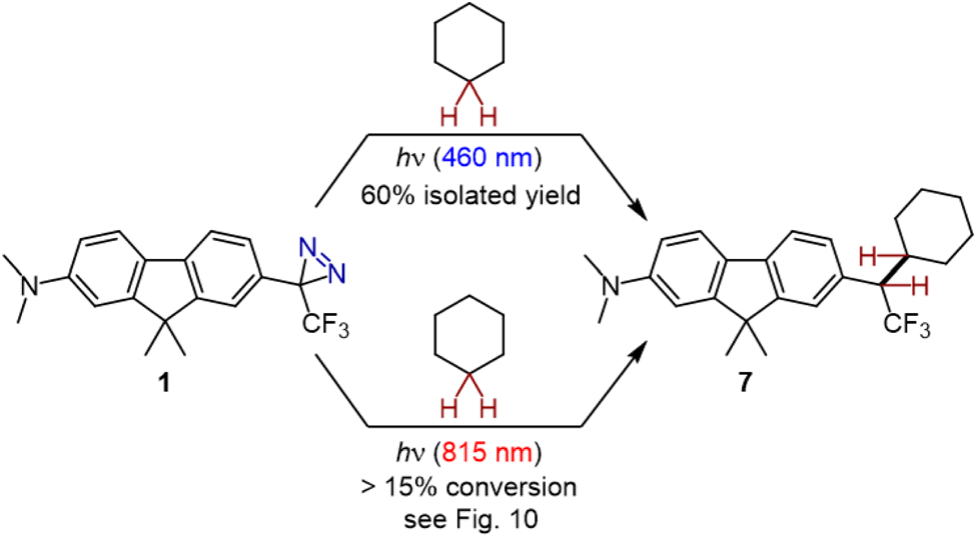
Activation of diazirine 1 with long-wavelength light, in the presence of cyclohexane, leads to C–H insertion of unactivated aliphatic bonds.

In the event, irradiation of diazirine 1 in cyclohexane with 460 nm light (supplied by commercial LED strip lights with a narrow emission profile; refer to the ESI for details†) led to rapid consumption of the diazirine group. We were pleased to observe the formation of the desired adduct 7 in 60% isolated yield (Scheme 2). The production of 7 was confirmed by high-resolution mass spectrometry, as well as a variety of NMR methods. ^1^H-coupled ^19^F NMR spectroscopy was particularly diagnostic; the splitting of the signal corresponding to the trifluoromethyl group (see Fig. S41 in the ESI†) confirmed the presence of the neighbouring proton in the target compound. The ^19^F NMR chemical shift (−63.26 ppm) was also consistent with previously reported cyclohexane adducts.^17,23,25^ Quantum yields of up to 0.88 were subsequently determined for the reaction of 1 with cyclohexane (refer to the ESI for details†).

To the best of our knowledge, the experiment described above represents the first time that any trifluoromethyl aryl diazirine reagent has been successfully activated with >450 nm light, without the use of a separate photosensitizer. The observation of C–H insertion product 7 under these conditions provides strong evidence for the effective conjugation of the diazirine group to the extended π system. Moreover, the high conversion for the reaction leading to 7 (refer to Fig. S38 in the ESI† for the crude ^19^F NMR spectrum showing reaction progress) indicates that the electron-donating dimethylamino group on the opposite ring is helping to stabilize the singlet state of the carbene—although certainly the effect of this electron-donating substituent is tempered by the fact that it is two rings distal to the reactive centre.

Push-pull chromophores based upon 9,9-dimethylfluorene scaffolds are known to have respectable 2-photon cross sections, considering their small size.^31^ This, together with the successful photo-activation documented above, suggested the intriguing possibility that we might be able to activate compound 1 using near-IR light. We therefore irradiated a capillary containing a solution of 1 in cyclohexane, in a two-photon microscope equipped with an 815 nm laser. The laser power was set low enough to avoid any unwanted heating of the sample (confirmed by direct measurement using an infrared probe, and also by the absence of cyclohexane evaporation under the conditions of the experiment).

**Fig. 10:**
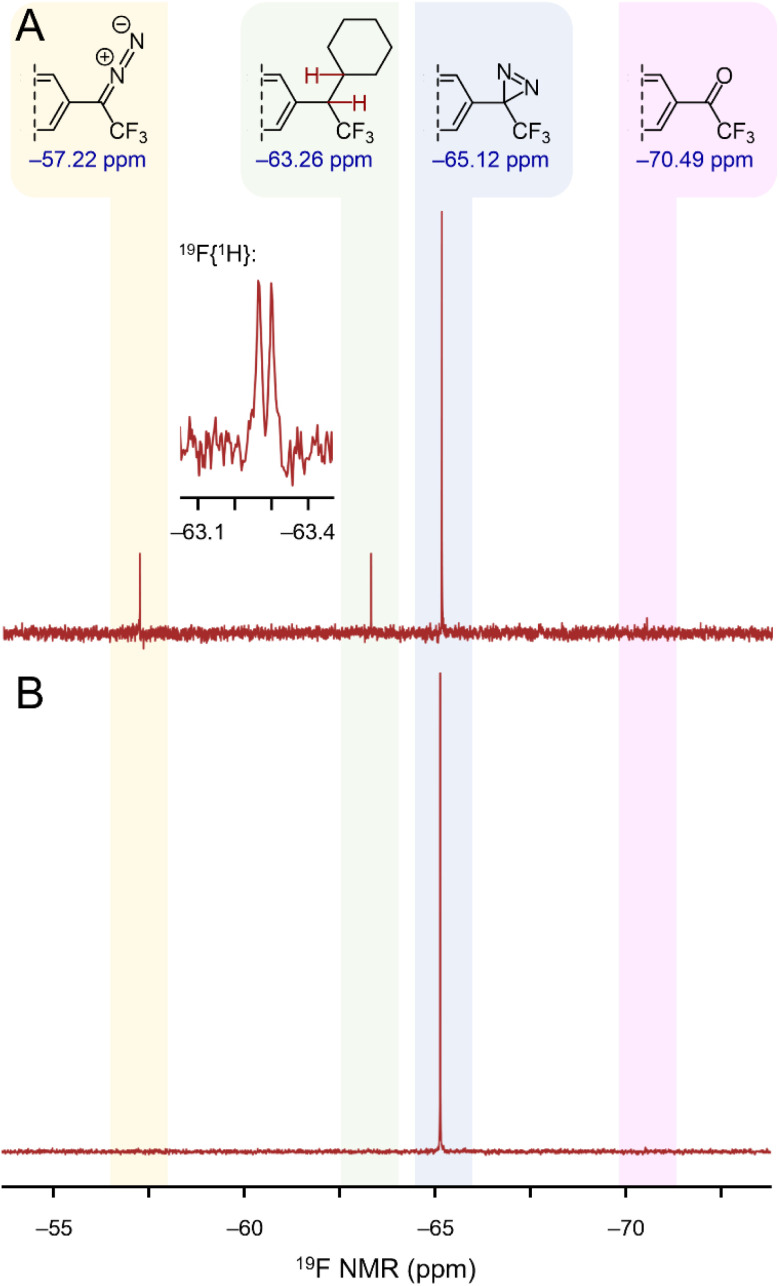
^19^F NMR spectra illustrating the activation of diazirine 1 using near-IR light. (A) NMR spectrum following irradiation of 1 in cyclohexane with 815 nm light. (B) Control sample stored in an amber vial. The inset shows a ^1^H-coupled ^19^F NMR spectrum, which confirms the presence of cyclohexane adduct 7.

Remarkably, ^19^F NMR analysis of the irradiated sample revealed partial conversion of the diazirine to a mixture of the desired C–H insertion product 7 (Fig. 10) and the corresponding linear diazo isomer (a well-known photo-isomerization product for diazirines).^32^ A control sample (maintained in the dark for an equivalent period of time) showed neither the linear diazo isomer nor the C–H insertion product. While the percent conversion in the experiment remained low—a consequence of the fact that the laser irradiated only a small portion of the capillary's total volume—we were able to confirm the presence of adduct 7 from the chemical shift of the new signal (−63.26 ppm in the ^19^F NMR spectrum) and from the fact that this signal split into a doublet when ^1^H-coupling was enabled during the NMR acquisition (inset to Fig. 10). The presence of compound 7 was further confirmed by mass spectrometry.

In an additional control experiment, a representative *p*-alkoxyphenyl trifluoromethyl diazirine (which is known to undergo C–H insertion to cyclohexane in >90% yield, under both photochemical and thermal stimulation, and which has *T*_onset_ and *T*_peak_ values almost identical to those of 1 (ref. 23)) was irradiated with near-IR light in an identical setup to that described above. This control molecule did not activate under the conditions of the experiment, and clean starting material was recovered following multiple irradiation cycles (refer to the ESI for details†). These results rule out any thermal activation in the experiment shown in Fig. 10, and establish that diazirine 1 was activated through two-photon excitation. Subsequent experimentation (refer to the ESI for details†) revealed a two-photon cross-section for diazirine 1 of 21 GM, at 800 nm and 2.3 mW of irradiation.

**Fig. 11:**
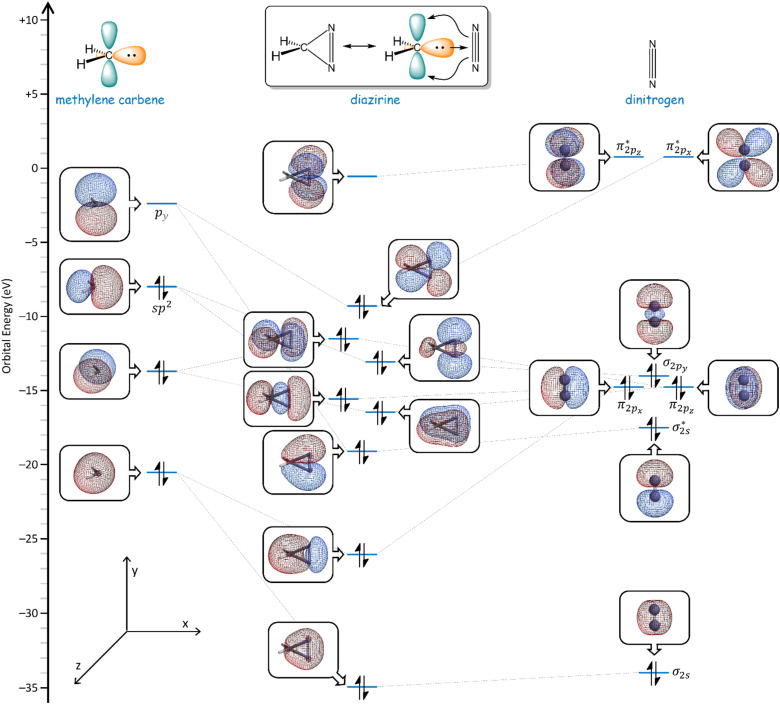
Calculated frontier molecular orbitals (M06-2X/6-311+G(2df,2p)) for the parent diazirine molecule (H_2_CN_2_), and correlations to the calculated orbitals of methylene carbene and dinitrogen.

Taken together, the data presented herein underline the need for a deeper consideration of the molecular orbitals associated with the diazirine group, pursuant to the construction of advanced diazirine–enabled reagents and biological probe molecules. To this end, we carried out a full molecular orbital analysis of the parent diazirine molecule (H_2_CN_2_). As shown in Fig. 11, the diazirine orbitals can be readily described by considering a linear combination of an sp^2^-hybridized methylene carbene (H_2_C:) and a molecule of dinitrogen (N_2_). This treatment is particularly helpful in illustrating how the diazirine LUMO is derived almost solely from the 
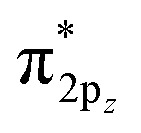
 antibonding orbital of N_2_, while the HOMO is derived from an in-phase (*i.e.* bonding) combination of the methylene p_*y*_ orbital and the dinitrogen 
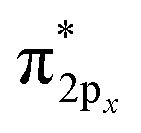
 orbital. A second in-phase combination, between the methylene sp^2^ orbital and the dinitrogen 
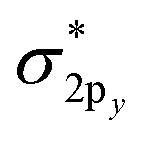
 orbital, constitutes another important bonding interaction. In this way, both frontier orbitals of the methylene carbene fragment can be seen to engage in bonding to the N_2_ fragment, in an overall picture that can be thought of as roughly analogous to that seen between transition metals and cyanide or carbon monoxide ligands, wherein sigma-bonding and π-backbonding interactions both contribute to the overall stabilization of the bond.

## Conclusions

Aryl diazirine reagents are ubiquitous in the chemical biology field, and yet in the vast majority of cases, the aromatic rings incorporated into the diazirine motif have been limited to simple monosubstituted benzenes. While the analogous azide reagents (as well as some representative diazoalkanes) have been extended to include better chromophores, permitting longer-wavelength activation, no such investigations have been made for trifluoromethyl aryl diazirines, perhaps because of a perception that the saturated central carbon atom would not permit effective conjugation to an extended π system.

In this work, we presented a complementary set of X-ray structural, computational, spectroscopic, and reactivity data in support of the contention that a diazirine's central carbon atom can be more accurately viewed as being sp^2^-hybridized, and where p-orbital contributions from this atom to the frontier molecular orbitals permit effective conjugation between the diazirine unit and the neighbouring aromatic ring.

The revised conceptual picture of diazirine bonding suggests the use of extended chromophores to allow longer-wavelength activation of the diazirine group to the corresponding carbene. As a proof of principle, we synthesized a representative diazirine–fluorene conjugate, and showed that this could be activated with >450 nm blue light, or through two-photon excitation using near-IR light. Both of these observations constitute important firsts for the diazirine field, and suggest the development of improved diazirine-containing reagents for carrying out biological target identification in living organisms.

## Author contributions

L. M. and T. S. synthesized diazirine 1, and carried out C–H insertion experiments with cyclohexane using visible light. S. V. synthesized diazirine 1′. L. M. and S. V. performed the activation using 815 nm light, and also measured the UV/Vis spectra. E. R. and R. E. measured the 1-photon quantum yields of 1, and also determined the 2-photon cross-section in cyclohexane. L. C. Q. and K. C. performed the initial synthesis of trifluoromethyl ketone 2 that initiated the research, while working under the supervision of W. D. H. Synthesis and crystallization of 3,5-bis(3-(trifluoromethyl)diaziridin-3-yl)pyridine was performed by M. L., and the corresponding X-ray structure was solved by A. O. The raw data for the plot in Fig. 1 were provided by S. M. Computational work was performed by G. A. D. and J. W. The manuscript was written by J. W. with the input of all authors.

## Conflicts of interest

There are no conflicts to declare.

## Supplementary Material

SC-016-D4SC06427E-s001

SC-016-D4SC06427E-s002

## Data Availability

Crystallographic data for 3,5-bis(3-(trifluoromethyl)diaziridin-3-yl)pyridine have been deposited at the Cambridge Structural Database under CCDC 2369820. All other data supporting this article have been included as part of the ESI.†
