# Supplementary material for: A diazirine's central carbon is sp2-hybridized, facilitating conjugation to dye molecules
Source: Chem Sci. 2024 Dec 2;16(2):970–9. doi: 10.1039/d4sc06427e (PMC11629510; doi:10.1039/d4sc06427e)
Supplement: SC-016-D4SC06427E-s001 [file SC-016-D4SC06427E-s001.pdf]

Supplementary Information (SI) for Chemical Science.
This journal is © The Royal Society of Chemistry 2024

Electronic Supplementary Information for:

A diazirine's central carbon is sp^2-hybridized, facilitating conjugation to dye molecules

Lorenzo Michelini, Tanya Slaney, Seerat Virk, Estefanía Rafic, Li Qie, Klara Corejova, Mathieu L. Lepage, Stefania F. Musolino, Allen G. Oliver, Roberto Etchenique, David Hong, Gino A. DiLabio and Jeremy E. Wulff

Index

Supplemental Tables 2

Table S1: Mulliken population analysis for the HOMO–2 of trifluoromethyl phenyl diazirine *2*
Table S2: X-ray structures for trifluoromethyl aryl diazirines reported in the CSD *3*
Table S3: X-ray structures for trifluoromethyl aryl diaziridines reported in the CSD *4*

Materials and Methods 5

General considerations: *5*

Synthesis of Diazirine **1** 6

Synthesis of Control Diazirine **1'** 26

Preparative Scale C–H Insertion Reactions 36

Photochemical insertion in cyclohexane with visible light (blue, 460 nm): *36*
Thermal insertion in cyclohexane: *41*
Two-photon activation experiments: *42*
General protocol for two-photon activation experiment: *42*
General protocol for control experiment: *42*
^{19}F-NMR Spectra acquired from microscope experiments: *43*
*Two-photon control experiment using bis-diazirine **S.12*** *45*

Photophysical Characterization of Diazirine **1** 47

Determination of one-photon photochemical parameters *47*
One-photon fluorescence measurements *49*
Determination of two-photon absorption cross-section *50*

Differential Scanning Calorimetry (DSC) 52

General protocol for DSC analysis *52*

Supplementary Figures 54

UV-Vis spectra in MeOH *54*
UV-Vis spectra in cyclohexane *58*
Home-made photo chamber *61*

References 63

Supplemental Tables

Table S1: Mulliken population analysis for the HOMO–2 of trifluoromethyl phenyl diazirine

<table>
<tr><th>Orbital Illustrations</th><th colspan="2">Contribution to $C_{Diazirine}$[(a)]</th></tr>
<tr><td rowspan="9">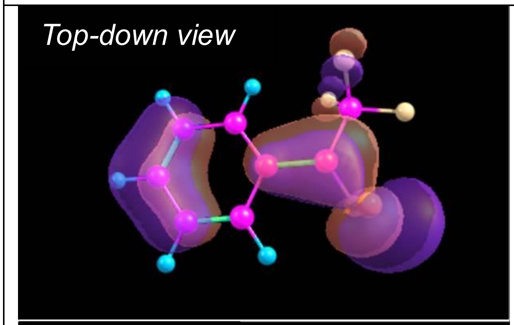
</td><td>1S</td><td>0.00000</td></tr>
<tr><td>2S</td><td>0.00000</td></tr>
<tr><td>2PX</td><td>0.00001</td></tr>
<tr><td>2PY</td><td>0.00003</td></tr>
<tr><td>2PZ</td><td>0.15628</td></tr>
<tr><td>3S</td><td>-0.00000</td></tr>
<tr><td>3PX</td><td>0.00001</td></tr>
<tr><td>3PY</td><td>0.00004</td></tr>
<tr><td>3PZ</td><td>0.24903</td></tr>
<tr><td rowspan="8">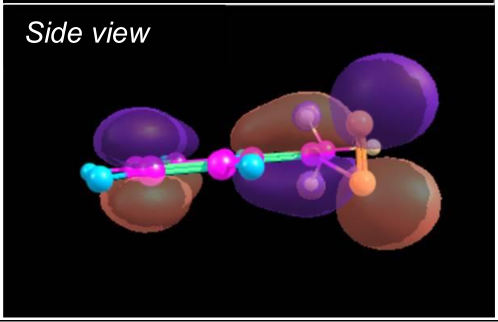
</td><td>4S</td><td>0.00001</td></tr>
<tr><td>4PX</td><td>0.00000</td></tr>
<tr><td>4PY</td><td>0.00002</td></tr>
<tr><td>4PZ</td><td>0.18731</td></tr>
<tr><td>5S</td><td>0.00047</td></tr>
<tr><td>5PX</td><td>0.00001</td></tr>
<tr><td>5PY</td><td>-0.00008</td></tr>
<tr><td>5PZ</td><td>-0.01396</td></tr>
</table>

(a) Arbitrary coordinate system used. Shading added for |coefficient| > 0.01.

Table S2: X-ray structures for trifluoromethyl aryl diazirines reported in the Cambridge Structural Database

Structure: YAVWOH	DOI: 10.1021/ja00071a017
$\varphi_{CF_3\text{-}C_{diazirine}\text{-}C_{ipso}\text{-}C_{ortho}}$	5.85°
$∡_{A\text{-}B\text{-}C}$	121.07°
Structure: YACIKIA	DOI: 10.1002/anie.202108421
$\varphi_{CF_3\text{-}C_{diazirine}\text{-}C_{ipso}\text{-}C_{ortho}}$	–17.17°
$∡_{A\text{-}B\text{-}C}$	121.25°
Structure: VEMQUB	DOI: 10.1021/jm300631e
$\varphi_{CF_3\text{-}C_{diazirine}\text{-}C_{ipso}\text{-}C_{ortho}}$	84.20°
$∡_{A\text{-}B\text{-}C}$	118.36°
Structure: NUJITIY	DOI: 10.1039/C9CC09891G
$\varphi_{CF_3\text{-}C_{diazirine}\text{-}C_{ipso}\text{-}C_{ortho}}$	24.36° / 7.19°
$∡_{A\text{-}B\text{-}C}$	120.70° / 122.16°
Structure: IQINOO	DOI: 10.1021/acs.joc.0c02993
$\varphi_{CF_3\text{-}C_{diazirine}\text{-}C_{ipso}\text{-}C_{ortho}}$	–2.34°
$∡_{A\text{-}B\text{-}C}$	121.93°
Structure: IQINII	DOI: 10.1021/acs.joc.0c02993
$\varphi_{CF_3\text{-}C_{diazirine}\text{-}C_{ipso}\text{-}C_{ortho}}$	–15.05°
$∡_{A\text{-}B\text{-}C}$	121.91°

Table S3: X-ray structures for trifluoromethyl aryl diaziridines reported in the Cambridge Structural Database

Structure: ZAKGIF	DOI: 10.1021/acs.orglett.1c03344
$\varphi_{CF_3\text{-}C_{diazirine}\text{-}C_{ipso}\text{-}C_{ortho}}$	63.79
$∡_{A\text{-}B\text{-}C}$	114.64
Structure: ZAKGEB	DOI: 10.1021/acs.orglett.1c03344
$\varphi_{CF_3\text{-}C_{diazirine}\text{-}C_{ipso}\text{-}C_{ortho}}$	68.98
$∡_{A\text{-}B\text{-}C}$	113.67
Structure: YAVWUN	DOI: 10.1021/ja00071a017
$\varphi_{CF_3\text{-}C_{diazirine}\text{-}C_{ipso}\text{-}C_{ortho}}$	95.02 / 82.18
$∡_{A\text{-}B\text{-}C}$	113.94 / 113.94
Structure: UJERIN	DOI: 10.1021/jacs.0c09403
$\varphi_{CF_3\text{-}C_{diazirine}\text{-}C_{ipso}\text{-}C_{ortho}}$	120.16
$∡_{A\text{-}B\text{-}C}$	115.32
Structure: UJERAF	DOI: 10.1021/jacs.0c09403
$\varphi_{CF_3\text{-}C_{diazirine}\text{-}C_{ipso}\text{-}C_{ortho}}$	66.97 / 69.52
$∡_{A\text{-}B\text{-}C}$	113.18 / 113.72
Structure: UJEQAE	DOI: 10.1021/jacs.0c09403
$\varphi_{CF_3\text{-}C_{diazirine}\text{-}C_{ipso}\text{-}C_{ortho}}$	60.90
$∡_{A\text{-}B\text{-}C}$	113.97

Materials and Methods

General considerations:

DFT calculations were performed on geometry-optimized structures, using the method and functional indicated in the text. For the plot in Figure 4, the relevant dihedral angle was constrained, and the rest of the molecule was subjected to geometry optimization. Gas-phase calculations were employed unless otherwise indicated. Calculations were performed in Spartan 18[12] and Gaussian 16.[13]

Commercial materials were used as received. THF was dried through a solvent purification system (SPS) and stored in oven-dried Strauss flask with molecular sieves (4 Å). Anhydrous cyclohexane (Sigma Aldrich, purity 99.5%) was used in all the diazirine activation experiments. Spectranalyzed™ MeOH and anhydrous cyclohexane (Sigma Aldrich, purity 99.5%) were used as solvents for UV-Vis spectra acquisition.

NMR spectra were acquired on either a Brucker AVANCE 300 (300.27 MHz for ^{1}H, 282.54 MHz for ^{19}F, 75.5 MHz for ^{13}C) or a Bruker AVANCE NEO 500 (500.27 MHz for ^{1}H, 470.72 MHz for ^{19}F, 125.7 MHz for ^{13}C) spectrometer. Chemical shifts were reported in part per million (ppm) and were calibrated to the central peak of residual NMR solvent (central peak of chloroform-d: ^{1}H NMR δ = 7.26 ppm, ^{13}C NMR δ = 77.16 ppm; methanol-d4: ^{1}H NMR δ = 3.31 ppm, ^{13}C NMR δ = 49.00 ppm). ^{13}C spectra and ^{19}F spectra were ^{1}H decoupled. Data are reported as follows: chemical shift (multiplicity [s = singlet, d = doublet, dd = doublet of doublet, t = triplet, q = quartet, ddd= doublets of doublets of doublets, dt = doublet of triplet, dq = doublet of quintet, m = multiplet], coupling constant in Hz, integration). Chemical shifts in ^{19}F spectra are reported in ppm and reported as obtained. Melting points were measured using a Gallenkamp melting point apparatus.

High resolution electrospray ionization mass spectrometry (HRMS) data were acquired using a Thermo Scientific Orbitrap Exactive Plus spectrometer using electrospray ionization experiments. IR spectra were recorded using a Cary 630 FTIR spectrometer. IR wave numbers (ν) are reported in cm^{-1}. UV-Vis spectra were recorded using a SpectraMax M5 Multi-Mode Microplate Reader. All data in MeOH were acquired using a 96-well plate (Greiner™, 96-well in cycloolefin copolymer [COC], Flat bottom). All data in cyclohexane were acquired using a quartz cuvette.

A home-made photoreactor equipped with LED Strip Lights emitting 460 nm light (Waveform Lighting, 220 W/m^2 measured with Pasco wireless light sensor [PS-3213]) was used for photochemical insertion experiments. A Zeiss confocal microscope LMS-880 equipped with a Ti: Saphire (Chameleon from Coherent) laser source was used for all two-photon activation experiments. The instrument settings for all two-photon microscope experiments are as follows: 10x objective, excitation wavelength 815 nm, laser output power 8%, irradiation area 855x855 μm, irradiation time for pixel of 2.4 μs. Differential Scanning Calorimetry analysis was performed using a DSC 25 TA instrument.

All diazirine-forming reactions were performed in the dark. Removal of solvent was done at 30 °C.

Synthesis of Diazirine 1:

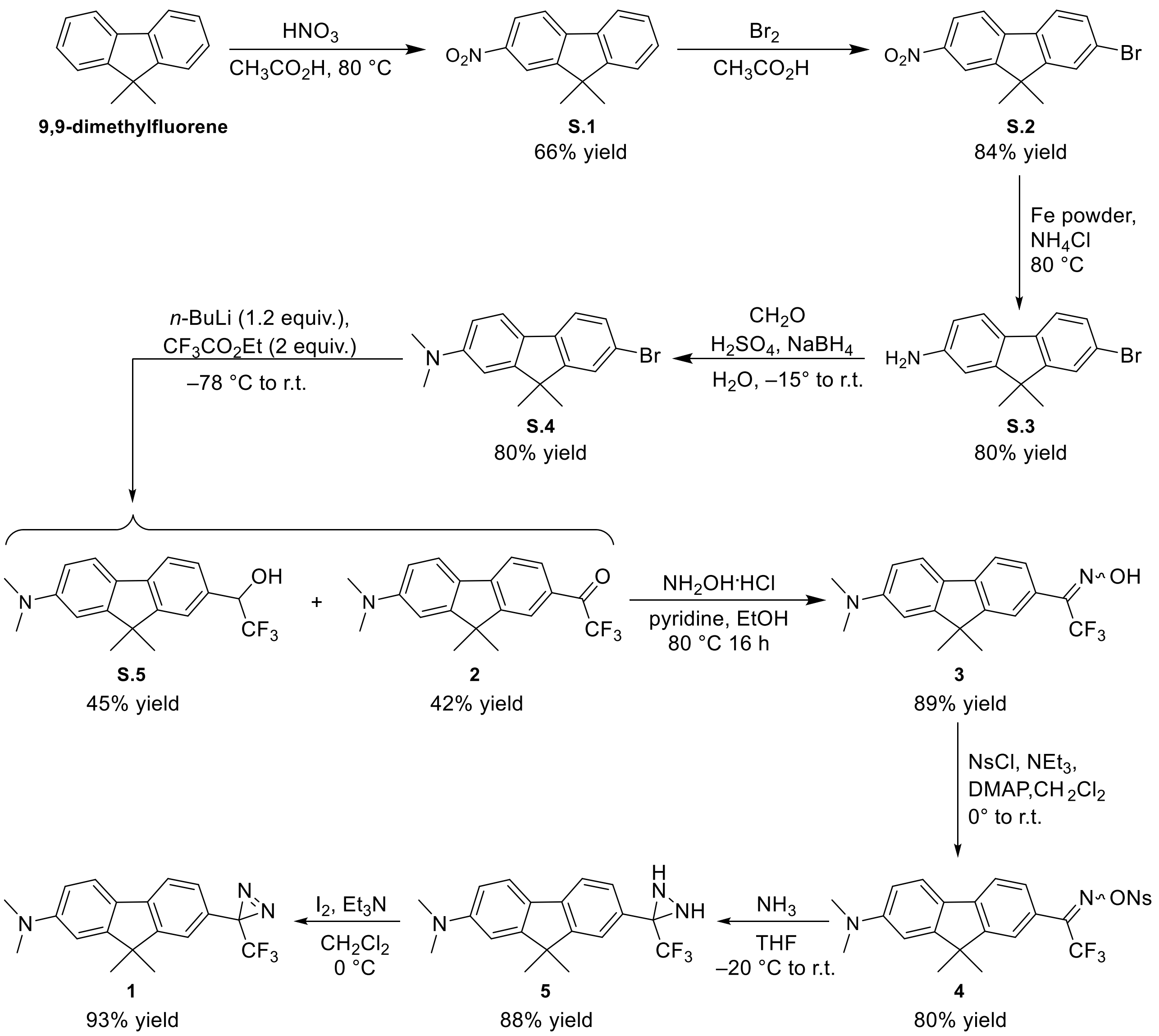

Figure S1. Synthetic scheme for the production of diazirine **1**.

Synthesis of 9,9-dimethyl-2-nitro-9H-fluorene (S.1):

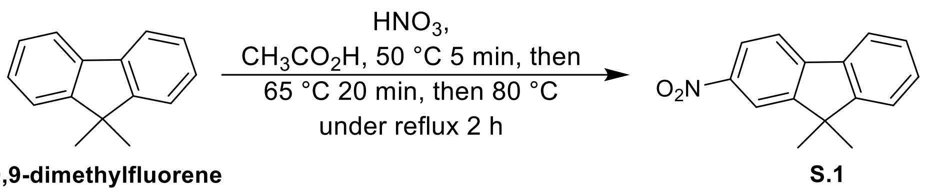

In a 1L round bottom flask, 9,9-dimethylfluorene (15.9 g, 81.8 mmol) was dissolved under stirring in 125 mL of acetic acid. The mixture was gently heated at 50 °C, and 20 mL of HNO_3 were slowly added to the solution forming a white precipitate. The mixture was stirred and gently heated at 65 °C until all the precipitate was dissolved. The solution obtained was heated under reflux at 80 °C for 2 h. The complete consumption of the starting material was verified by TLC (80% Hex, 20% Et_2O). Upon cooling the reaction at room temperature for 24 h a precipitate was formed. The product was collected on a glass frit funnel, washed with cold acetic acid three times and then dried to obtain **S.1** (12.7g, 57.2 mmol, 66 % yield) as a lime-yellowish solid with spectroscopic data in good accordance with the literature.[1,2] ^{1}H-NMR (300 MHz, $CDCl_3$) δ(ppm): 8.30 (dd, *J*= 2.1, 0.6 Hz, 1H, ArH), 8.27 (dd, *J*= 8.3, 2.1 Hz, 1H, ArH), 7.83-7.79 (m, 2H, ArH), 7.52-7.38 (m, 3H, ArH), 1.54 (s, 6H, CH_3).

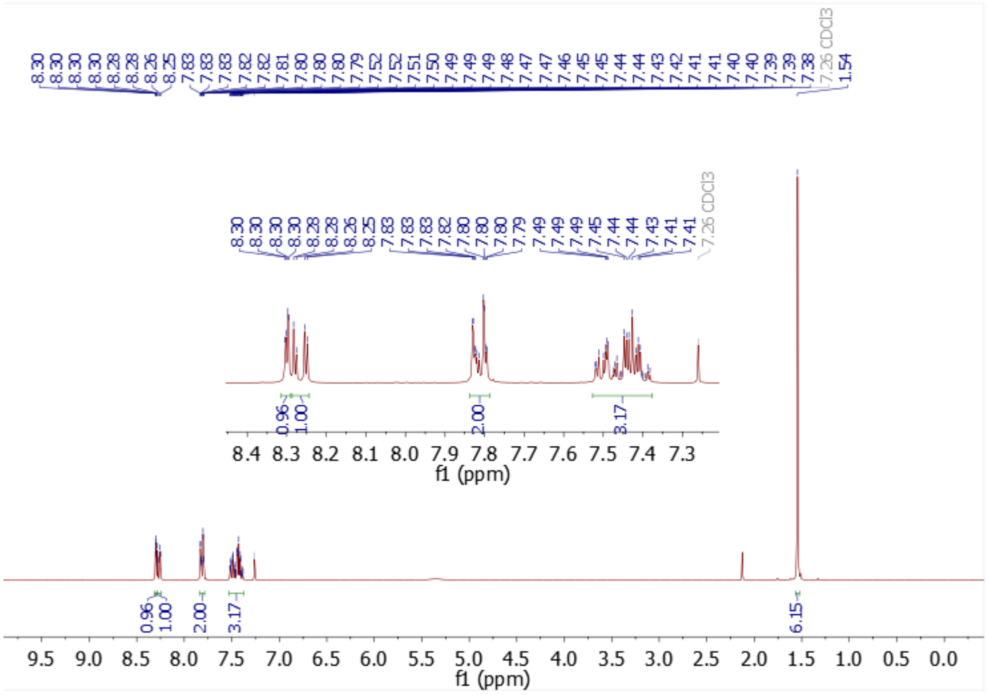

Figure S2. ^{1}H-NMR spectrum of **S.1** in $CDCl_3$.

Synthesis of 2-bromo-9,9-dimethyl-7-nitro-9H-fluorene (S.2):

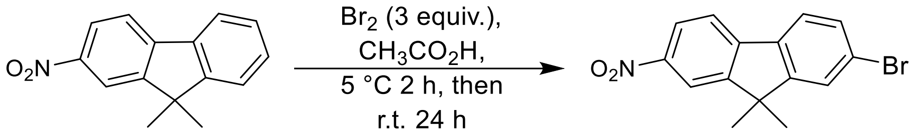

In a 250 mL round bottom flask, **S.1** (12.7 g, 53.1 mmol) was dissolved in 60 mL of acetic acid. The solution was cooled at 5 °C and Br_2 (8.2 mL, 159.3 mmol) was slowly added dropwise for 2 h through an addition funnel. The mixture was then allowed to reach room temperature and left under stirring for 24 h. The mixture was slowly quenched at 0 °C with a solution of KOH (1M) and extracted with DCM. The organic phase was then washed with a saturated solution of $Na_2S_2O_3$, dried over $MgSO_4$, and concentrated under vacuum. The crude material was purified through hot filtration followed by recrystallization in acetonitrile to afford the desired bromo compound **S.2** (14.2 g, 84% yield) as a yellow solid with spectroscopic data in good accordance with the literature.[3,4] ^{1}H-NMR (300 MHz, $CDCl_3$) δ(ppm): 8.29-8.24 (m, 2H, ArH), 7.79 (dd, *J*= 8.9, 1.3 Hz, 1H, ArH), 7.66 (dd, *J*= 8.1, 0.5 Hz, 1H, ArH), 7.63 (d, *J*= 1.3 Hz, 1H, ArH), 7.54 (dd, *J*= 8.1, 1.8 Hz, 1H, ArH), 1.54 (s, 6H, CH_3).

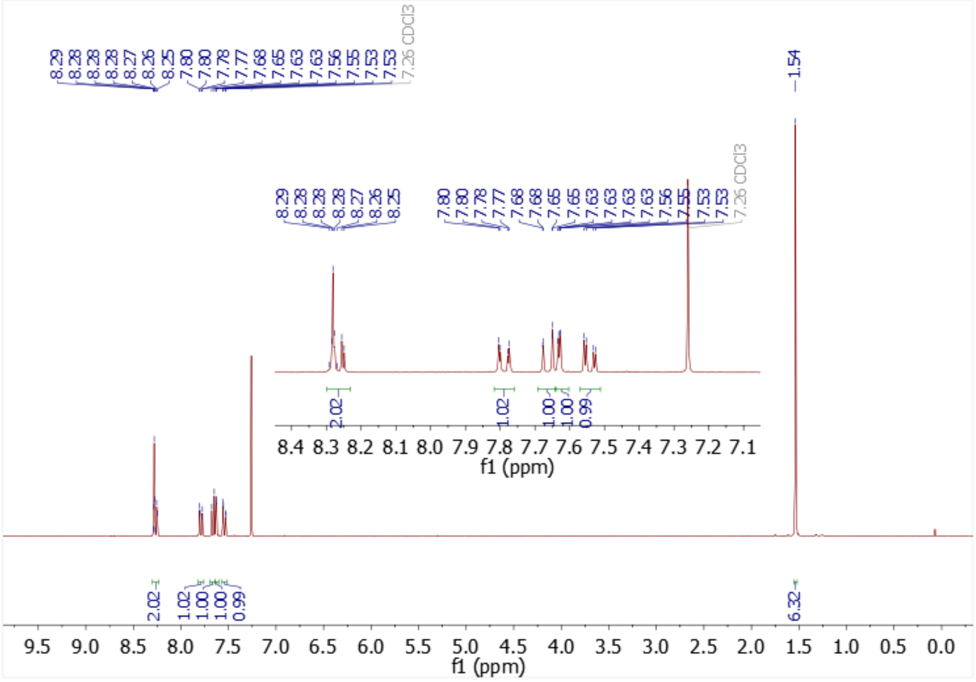

Figure S3: ^{1}H-NMR spectrum of **S.2** in $CDCl_3$.

Synthesis of 7-bromo-9,9-dimethyl-9H-fluoren-2-amine (S.3):

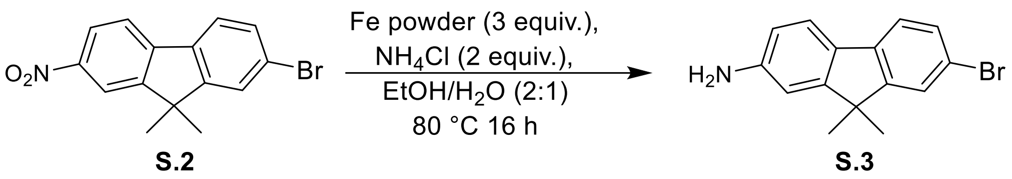

In a 1L three-neck round bottom flask, **S.2** (14.2 g, 44.6 mmol) was dissolved in 550 mL of a mixture of EtOH/H_2O (2:1). NH_4Cl (4.8 g, 89.7 mmol) and iron powder (7.5 g, 134.3 mmol, 70 mesh, <212 µm) was added to the reaction mixture under stirring. A condenser was installed on one of the necks and the other two were sealed with septa. The system was purged with argon and the flask was gently heated at 80 °C. The mixture was stirred under reflux for 16 h until complete consumption of **S.2** was observed by TLC (80% Hex, 20% Et_2O). The dark solution obtained was cooled at room temperature and a saturated solution of $NaHCO_3$ was added until reaching pH= 7. Subsequently, using a neodymium magnet to retain most of the iron nanoparticles in suspension, the crude was poured into a glass frit funnel, filtered and concentrated under vacuum affording **S.3** (10.4 g, 80 % yield) as a white solid with spectroscopic data in accordance with the literature.[5,6] ^{1}H-NMR (300 MHz, $CDCl_3$) δ(ppm): 7.48-7.44 (m, 2H, ArH), 7.41-7.37 (m, 2H, ArH), 6.73 (d, *J*= 2.1 Hz, 1H, ArH), 6.66 (dd, *J*= 8.1, 2.2 Hz, 1H, ArH), 3.79 (s, 2H, NH_2), 1.43 (s, 6H, CH_3).

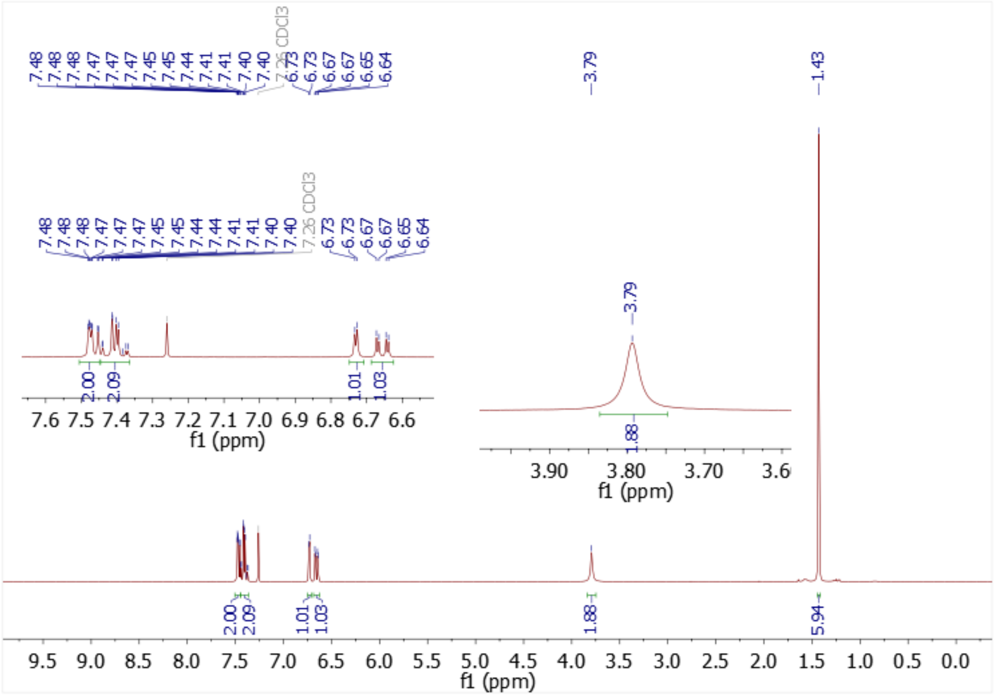

Figure S4. ^{1}H-NMR spectrum of **S.3** in $CDCl_3$.

Synthesis of 7-bromo-N,N,9,9-tetramethyl-9H-fluoren-2-amine (S.4):

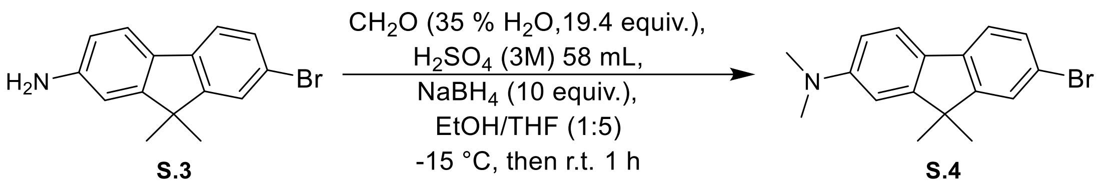

In a 1 L round bottom flask 55 mL of formaldehyde (35% in H_2O, 700.4 mmol), 78 mL of THF and 58 mL of H_2SO_4 (3M) were stirred at room temperature. The solution obtained was cooled down at −15 °C and a suspension of **S.3** (10.4 g, 36.1 mmol) and $NaBH_4$ (13.7 g, 361 mmol) in 167 mL of a mixture EtOH/THF (1:5) was added portionwise. Then the mixture was allowed to reach room temperature and was stirred for 1 h. The complete consumption of compound **S.3** was verified by TLC (70% Hex, 30% Et_2O). The crude mixture obtained was quenched with a saturated solution of $NaHCO_3$ and the aqueous phase was extracted with EtOAc. The combined organic phases were dried over Na_2SO_4, concentrated under vacuum, and recrystallized in EtOH leading to **S.4** (9.1 g, 80% yield) as a white crystal with spectroscopic data in accordance with the literature.[7] ^{1}H-NMR (300 MHz, $CDCl_3$) δ(ppm): 7.54 (d, *J*= 8.2 Hz, 1H, ArH), 7.47 (d, *J*= 1.7 Hz, 1H, ArH), 7.43 (d, *J*= 8.0 Hz, 1H, ArH), 7.38 (dd, , *J*= 8.0 Hz, 1.7 Hz 1H, ArH), 6.75-6.70 (m, 2H, ArH), 3.03 (s, 6H, NCH_3), 1.45 (s, 6H, CH_3).

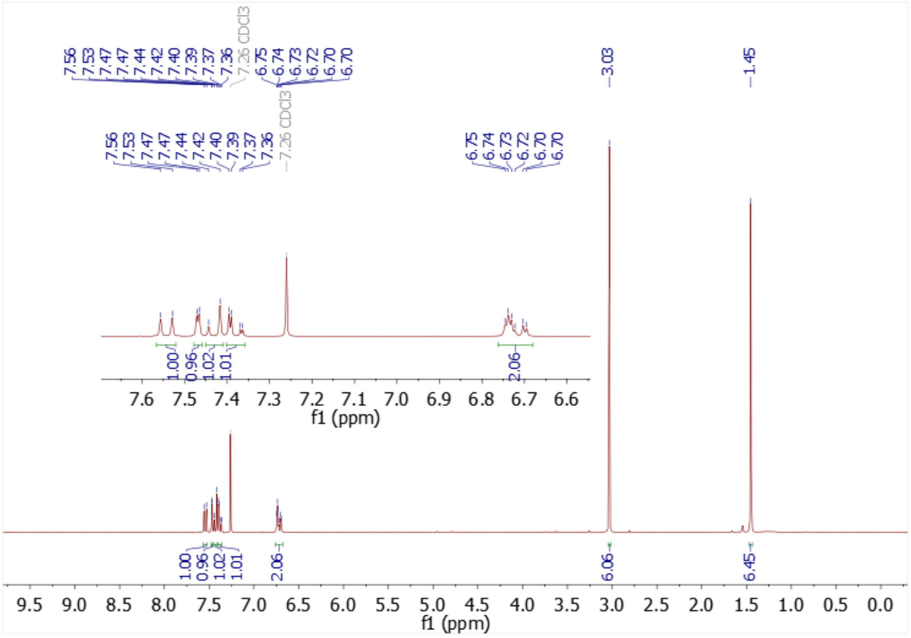

Figure S5. ^{1}H-NMR spectrum of **S.4** in $CDCl_3$.

Synthesis of 1-(7-(dimethylamino)-9,9-dimethyl-9H-fluoren-2-yl)-2,2,2-trifluoroethan-1-one (2) and 1-(7-(dimethylamino)-9,9-dimethyl-9H-fluoren-2-yl)-2,2,2-trifluoroethan-1-ol (S.5):

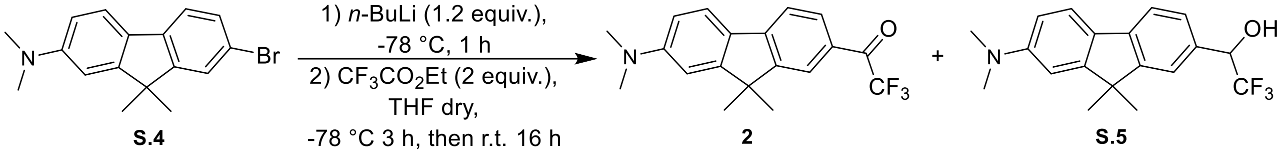

S.4 **2** **S.5**

A solution of **S.4** (3.1 g, 10 mmol) in 95 mL of dry THF was prepared in a 250 mL flame-dried two-neck round bottom flask and cooled down at −78 °C. Under argon atmosphere, *n*-butyl lithium 2.5 M in *n*-hexane (5 mL, 12 mmol) was added dropwise to the solution. The mixture obtained was left under stirring at −78 °C for 1 h and ethyl trifluoroacetate (2.4 mL, 20 mmol) was added dropwise to the mixture that was left under stirring at −78 °C for 3 h. After this time the reaction was allowed to warm up at room temperature slowly and after 16 h stirring was quenched with a saturated solution of NH_4Cl. The aqueous phase was extracted with EtOAc, dried over Na_2SO_4, and concentrated under vacuum. The crude product mixture was purified by flash-column chromatography (90% Hex, 10% Et_2O) leading to **2** (1.4 g, 42% yield) as an orange solid and to **S.5** (1.5 g, 45% yield) as a yellowish solid.

Characterization data for **2**: Melting point: 76-80 °C. ^{1}H-NMR (500 MHz, $CDCl_3$) δ(ppm): 8.06 (s, 1H, ArH), 8.02 (d, *J*= 8.1 Hz, 1H, ArH), 7.64 (t, *J*= 9.0 Hz, 2H, ArH), 6.75-6.74 (m, 2H, ArH), 3.09 (s, 3H, NCH_3), 1.51 (s, 6H, CH_3). ^{13}C-NMR (500 MHz, $CDCl_3$) δ(ppm): 179.97 (q, *J*= 34.3 Hz), 157.66, 153.38, 152.11, 148.02, 130.69 (q= 2.5 Hz), 126.48, 125.75, 124.01, 122.79, 118.45, 116.16, 111.80, 105.78, 47.02, 40.76, 27.31. ^{19}F-NMR (283 MHz, $CDCl_3$) δ(ppm): −70.47. $\lambda_{max\ (MeOH)}$= 318 nm, $\lambda_{abs\ (MeOH)}$= 421 nm. IR: 691.4, 736.1, 754.8, 779.0, 807.0, 844.2, 961.7, 978.4, 1071.6, 1142.4, 1164.8, 1196.5, 1362.3, 1435.0, 1507.7, 1591.6, 1695.9, 2808.5, 2860.7, 2926.0, 2963.2. HRMS (ESI+) m/z $[MH]^+$ calculated for $C_{19}H_{19}F_3NO$: 334.1413, found: 334.1414.

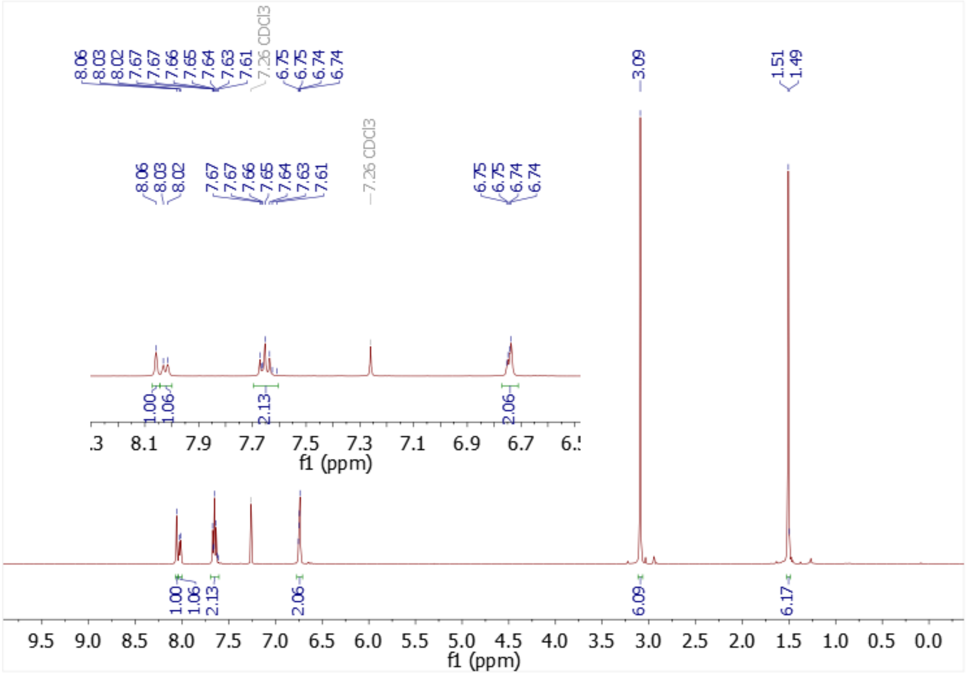

Figure S6. ^{1}H-NMR spectrum of **2** in $CDCl_3$.

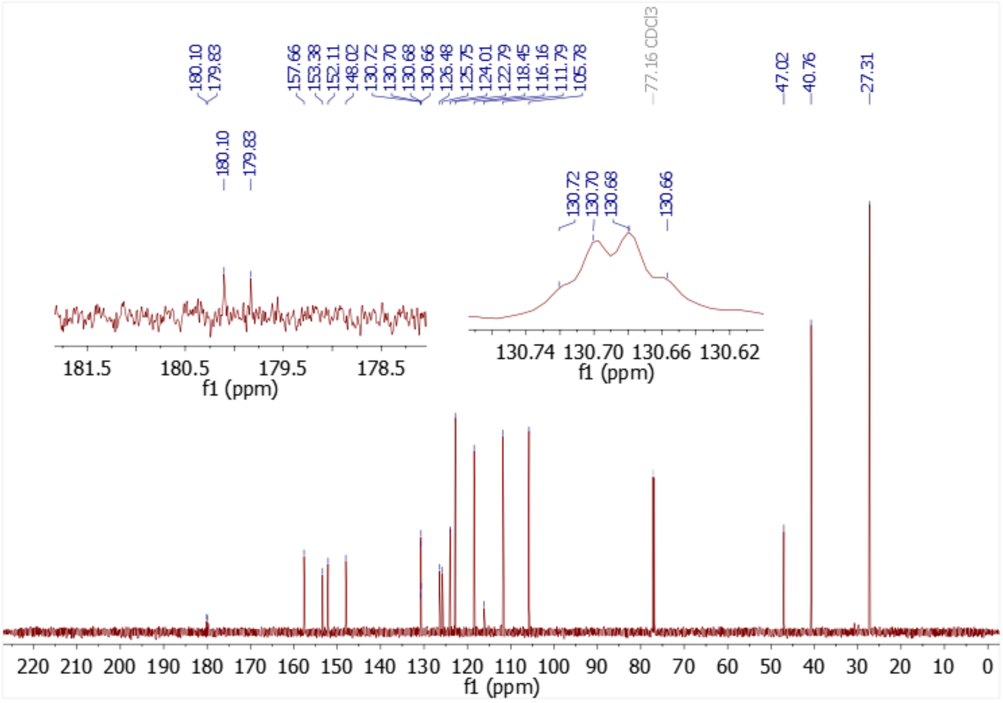

Figure S7. ^{13}C-NMR spectrum of **2** in $CDCl_3$.

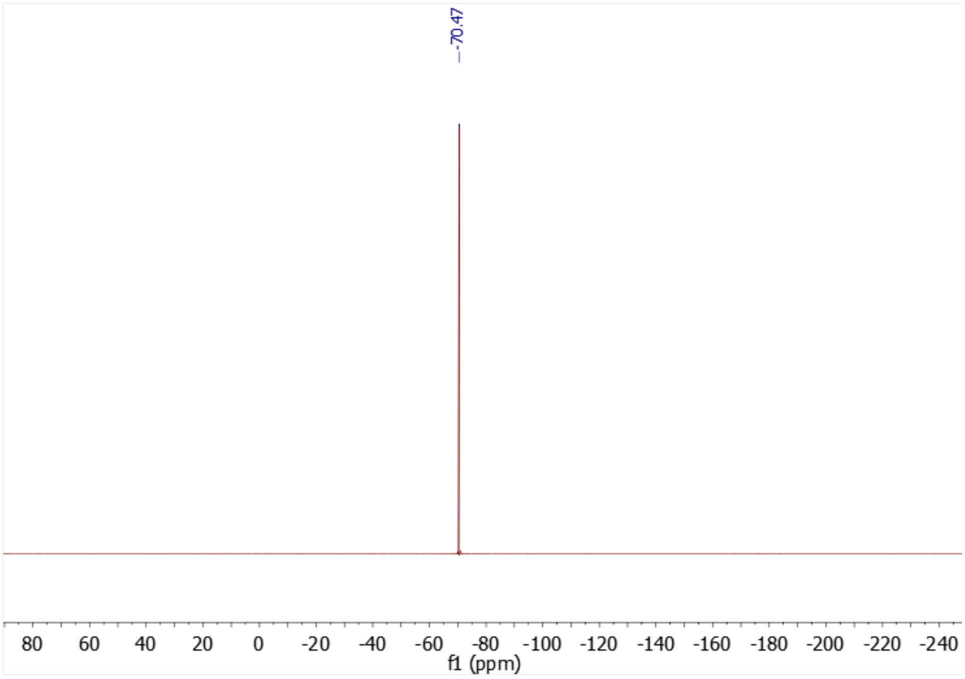

Figure S8. ^{19}F-NMR spectrum of **2** in $CDCl_3$.

Characterization data for **S.5**: Melting point: 137-140 °C. ^{1}H-NMR (500 MHz, $CDCl_3$) δ(ppm): 7.59 (dd, *J*= 8.2, 2.1 Hz, 1H, ArH), 7.46 (s, 1H, ArH), 7.46 (s, 1H, ArH), 7.36 (d, *J*= 7.8 Hz ,1H, ArH), 6.77 (d, *J*= 2.4 Hz ,1H, ArH), 6.73 (dd, *J*= 8.4, 2.4 Hz, 1H, ArH), 5.05 (q, *J*= 6.8 Hz, 1H, ArCH), 3.04 (s, 6H, NCH_3), 2.58 (s, 1H, OH), 1.48 (s, 6H, CH_3). ^{13}C-NMR (500 MHz, $CDCl_3$) δ(ppm): 155.78, 153.44, 151.05, 141.59, 130.81, 127.93, 127.45, 126.57, 124.56 (q, *J*= 282.0 Hz), 121.51, 121.20, 118.64, 111.71, 106.57, 73.48 (q, *J*= 31.8 Hz), 46.98, 41.06, 27.57. F^{19}-NMR (283 MHz, $CDCl_3$) δ(ppm): −78.14. $\lambda_{max\ (MeOH)}$= 317 nm. IR (ν): 680.2, 708.2, 739.9, 779.0, 807.0, 842.4, 909.5, 961.7, 1004.5, 1073.5, 1123.8, 1164.8, 1217.0, 1269.2, 1295.2, 1358.6, 1382.8, 1435.0, 1477.9, 1505.8, 1578.5, 1612.1, 2804.8, 2860.7, 2924.1, 2961.4, 3412.4. HRMS (ESI+) m/z $[MH]^+$ calculated for $C_{19}H_{21}F_3NO$: 336.1569, found: 336.1567.

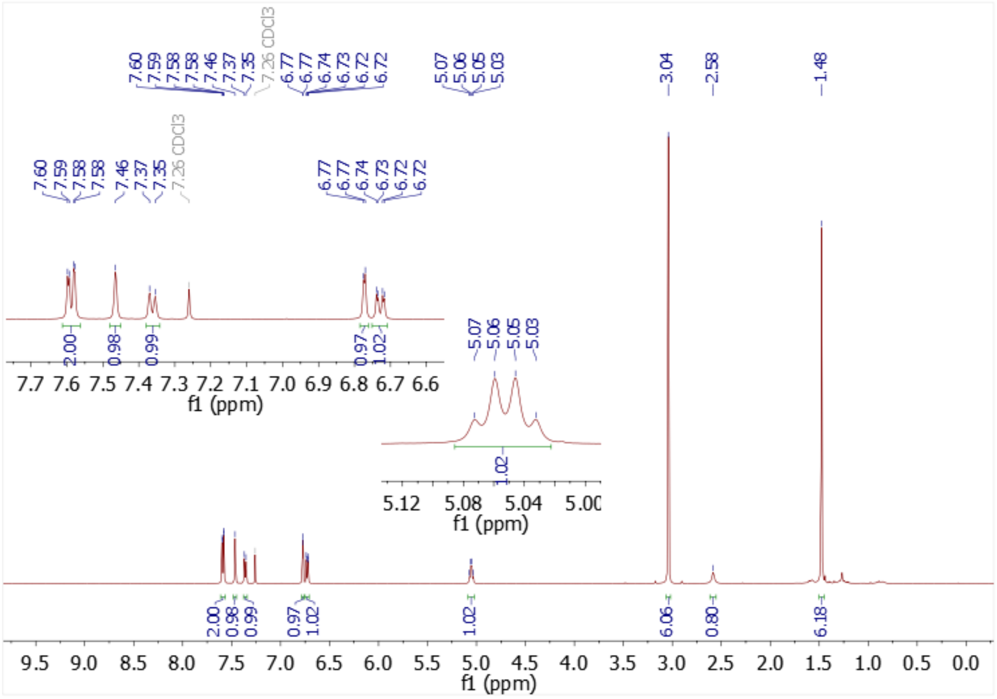

Figure S9. ^{1}H-NMR spectrum of **S.5** in $CDCl_3$.

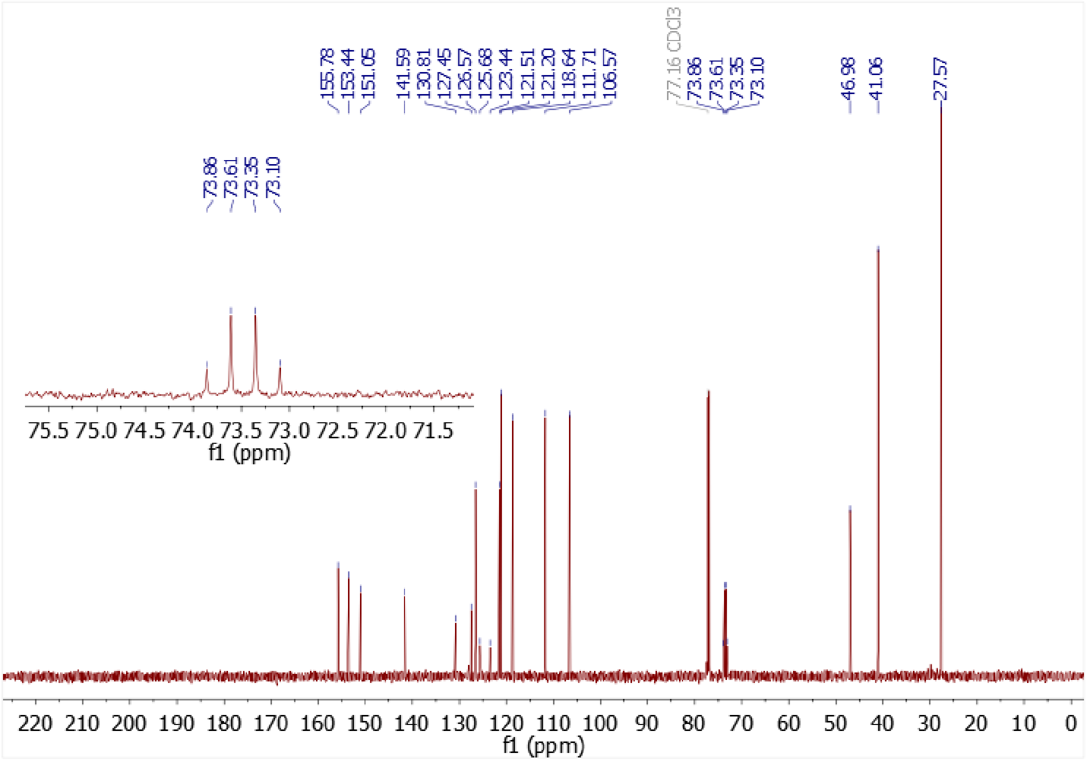

Figure S10. ^{13}C-NMR spectrum of **S.5** in $CDCl_3$.

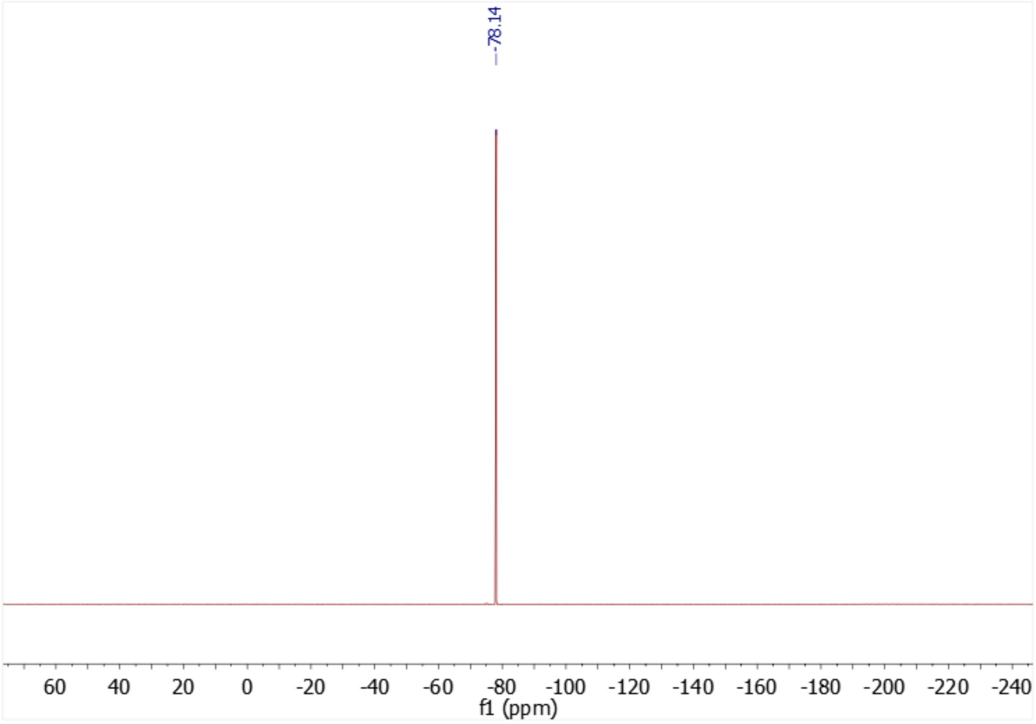

Figure S11. ^{19}F-NMR spectrum of **S.5** in $CDCl_3$.

Synthesis of 1-(7-(dimethylamino)-9,9-dimethyl-9H-fluoren-2-yl)-2,2,2-trifluoroethan-1-one oxime (3):

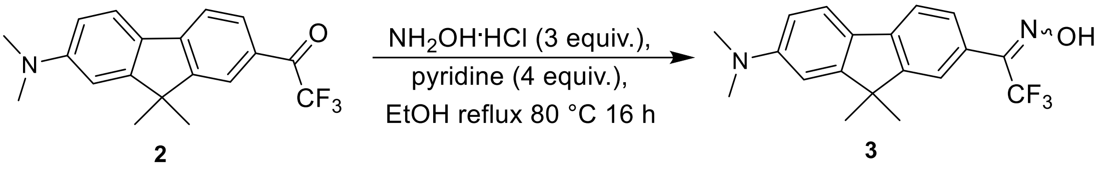

A solution of **2** (280.9 mg, 0.84 mmol) in 7.5 mL of absolute EtOH was prepared in a 50 mL round bottom flask. Under stirring, pyridine (270 µL, 3.4 mmol) and hydroxylamine hydrochloride (175.7 mg, 2.5 mmol) were added to the solution. The mixture obtained was heated under reflux at 80 °C for 16 h then was treated with a 1M solution of HCl and the aqueous phase was extracted several times with Et_2O. The combined organic phases were washed with water until reaching pH= 7, dried over Na_2SO_4, and finally concentrated under vacuum. The crude product **3**, obtained as a mixture of two geometrical isomers, was submitted to the next step without further purification.

On one occasion, for the purpose of NMR characterization, the crude was purified by flash chromatography (70% Hex, 30% Et_2O) to afford pure compound **3** (262.7 mg, 89% yield) as yellowish solid. Melting point: 153-156 °C. ^{1}H-NMR (500 MHz, $CDCl_3$) δ(ppm): 8.37 (s, 1H, OH), 7.63 (dd, *J*= 14.0, 8.1 Hz, 2H, ArH), 7.54 (s, 1H, ArH), 7.49 (d, *J*= 8.0 Hz ,1H, ArH), 6.77 (d, *J*= 2.3 Hz, 1H, ArH), 6.73 (dd, *J*= 8.4, 2.3 Hz, 1H, ArH), 3.06 (s, 6H, NCH_3), 1.49 (s, 6H, CH_3). ^{13}C-NMR (500 MHz, $CDCl_3$) δ(ppm): 156.14, 152.88, 151.31, 148.54 (q, *J*= 32.2 Hz), 142.64, 127.95, 127.10, 122.83, 121.14, 121.57, 119.92, 118.46, 111.74, 106.38, 47.11, 41.00, 40.93, 27.48. ^{19}F-NMR (283 MHz, $CDCl_3$) δ(ppm): −65.96 major isomer, −62.09 minor isomer. IR (ν): 732.4, 775.3, 807.0, 844.2, 909.5, 933.7, 963.5, 1026.9, 1073.5, 1131.2, 1176.0, 1218.8, 1284.1, 1358.6, 1382.8, 1412.7, 1435.0, 1474.2, 1505.8, 1576.7, 1606.5, 2806.7, 2864.5, 2903.6, 2926.0, 2965.1, 3233.5. HRMS (ESI+) m/z $[MH]^+$ calculated for $C_{19}H_{20}F_3N_2O$: 349.1522, found: 349.1520.

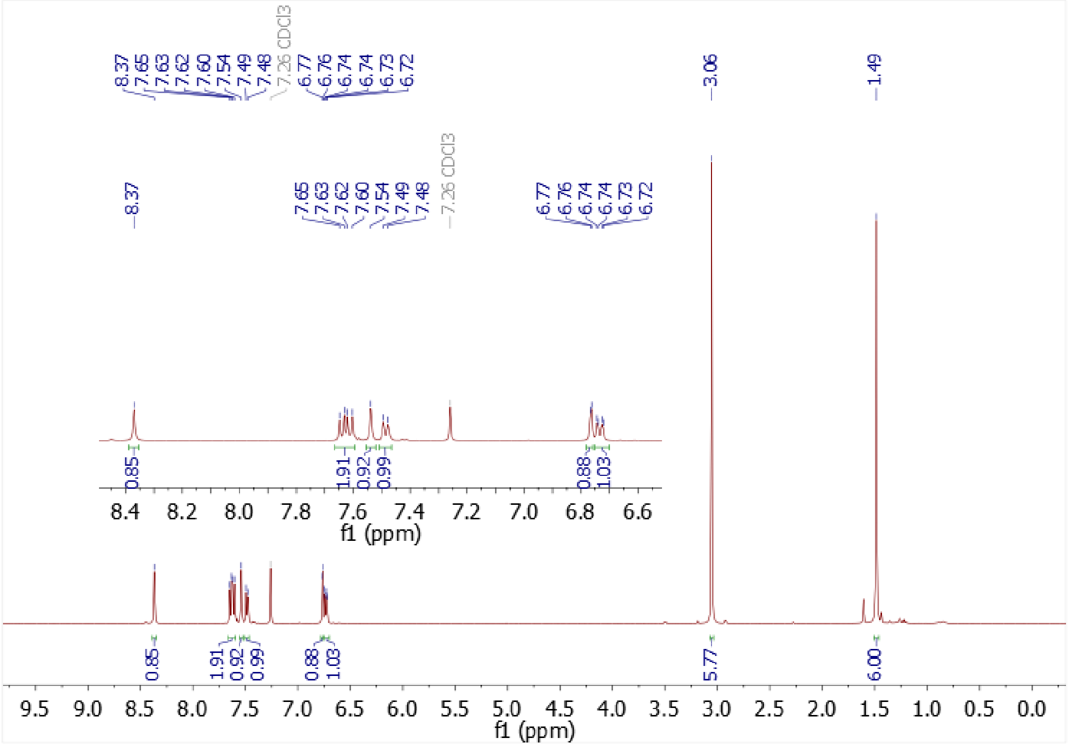

Figure S12. ^{1}H-NMR spectrum of **3** in $CDCl_3$.

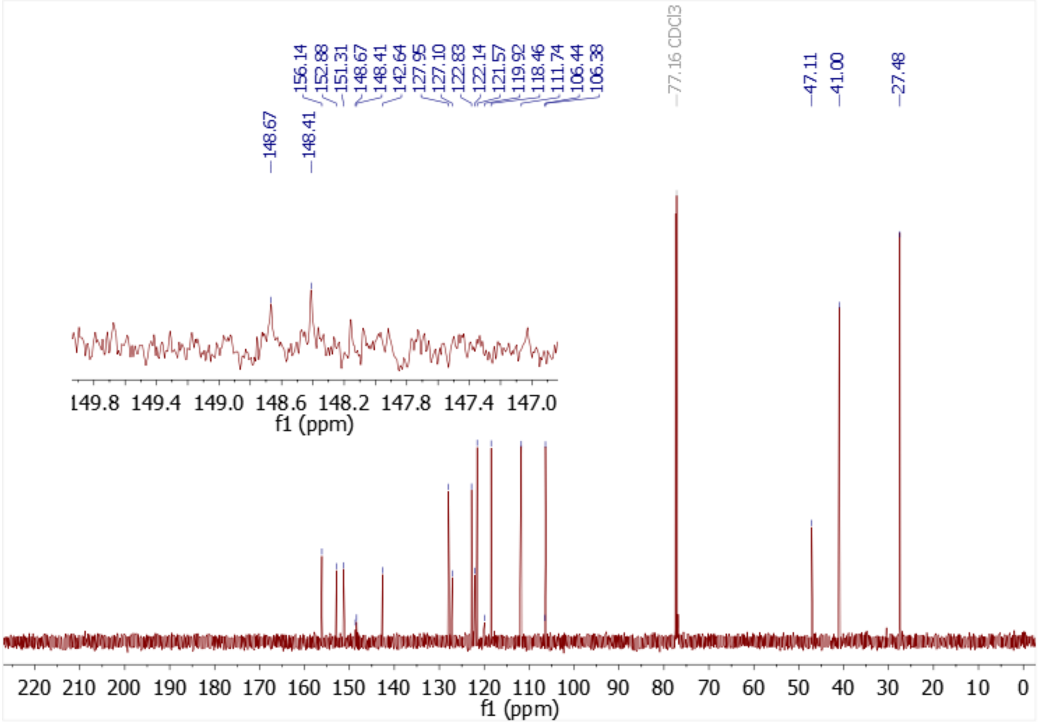

Figure S13. ^{13}C-NMR spectrum of **3** in $CDCl_3$.

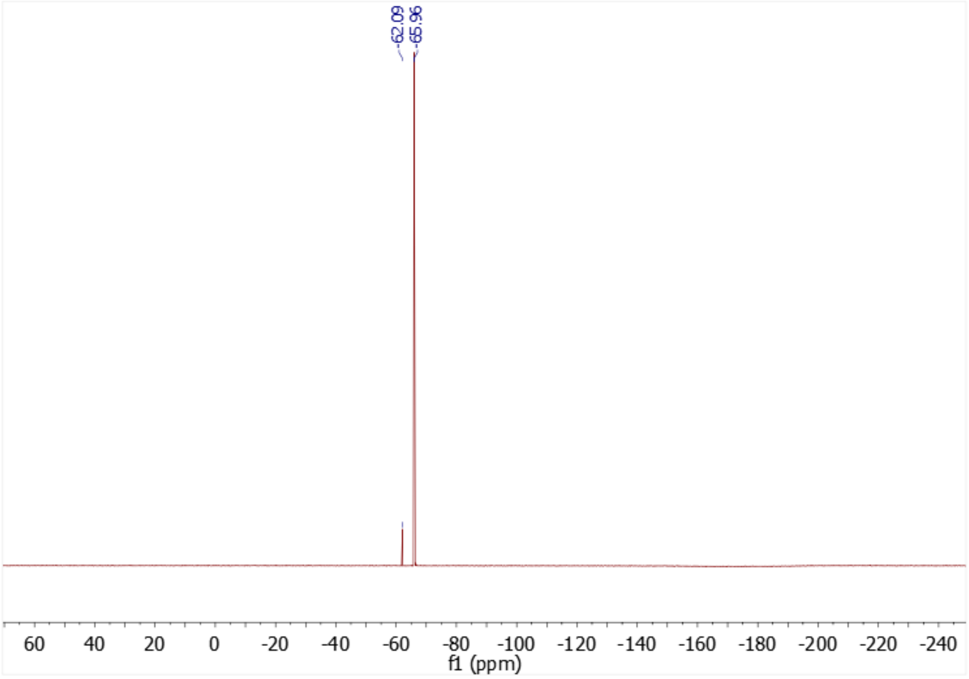

Figure S14. ^{19}F-NMR spectrum of **3** in $CDCl_3$.

Synthesis of 1-(7-(dimethylamino)-9,9-dimethyl-9H-fluoren-2-yl)-2,2,2-trifluoroethan-1-one O-((2-nitrophenyl) sulfonyl) oxime (4):

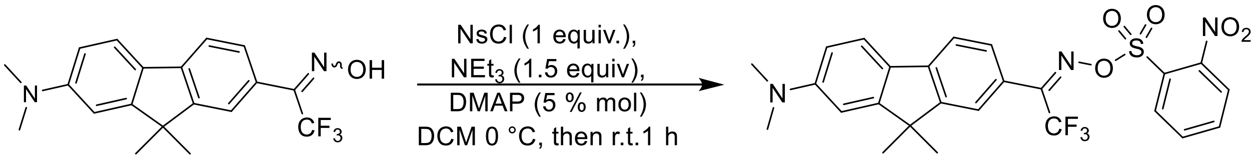

3 **4**

A solution of **3** (251.1 mg, 0.7 mmol) in 4 mL of DCM was prepared in a 25 mL round bottom flask. The solution was cooled down at 0 °C and NEt_3 (152 µL, 1.1 mmol), DMAP (4.3 mg 0.035 mmol), and 2-nitrobenzensulfonyl chloride (186.0 mg, 0.8 mmol) were added in this order. After 5 min, the mixture obtained was allowed to reach room temperature and was left under stirring for 1 h. Subsequently, the mixture was treated with NH_4Cl reaching approximately pH= 6 and the aqueous phase was extracted with Et_2O three times. The combined organic phases were dried over Na_2SO_4 and concentrated under vacuum. The crude product **4** obtained was submitted to the next step without further purification.

On one occasion, for the purpose of NMR characterization, the crude product was purified by flash chromatography (75% Hex, 25% Et_2O) to afford pure compound **4** (309.1 mg, 80% yield) as an

orange solid. Melting point: 117-120 °C. ^{1}H-NMR (500 MHz, $CDCl_3$) δ(ppm): 8.30 (dd, *J*= 7.8, 1.3 Hz, 1H, ArH), 7.92-7.79 (m, 3H, ArH), 7.70 (s, 1H, ArH), 7.64 (dd, *J*= 17.4, 8.2 Hz, 2H, ArH), 7.54 (d, *J*= 8.0 Hz, 1H, ArH), 6.79-6.70 (m, 2H, ArH), 3.07 (s, 6H, NCH_3), 1.51 (s, 6H, CH_3). ^{13}C-NMR (500 MHz, $CDCl_3$) δ(ppm): 156.66, 155.45 (q, J= 33.0 Hz), 153.12, 151.60, 148.93, 144.45, 135.87, 133.80, 132.27, 128.18, 127.74, 125.02, 123.53, 122.02, 121.12, 119.85, 118.90, 118.61, 111.70, 106.9, 47.23, 40.89, 27.30. ^{19}F-NMR (283 MHz, $CDCl_3$) δ(ppm): −65.21. IR (ν): 665.3, 695.1, 736.1, 788.3, 807.0, 836.8, 881.5, 909.5, 1021.3, 1073.5, 1153.6, 1176.0, 1198.3, 1297.1, 1362.3, 1399.6, 1436.9, 1474.2, 1507.7, 1546.8, 1597.2, 1694.1, 2806.7, 2862.6, 2927.8, 2963.2. HRMS (ESI+) m/z $[MH]^+$ calculated for $C_{25}H_{23}F_3N_3O_5S$: 534.1305, found: 534.1307.

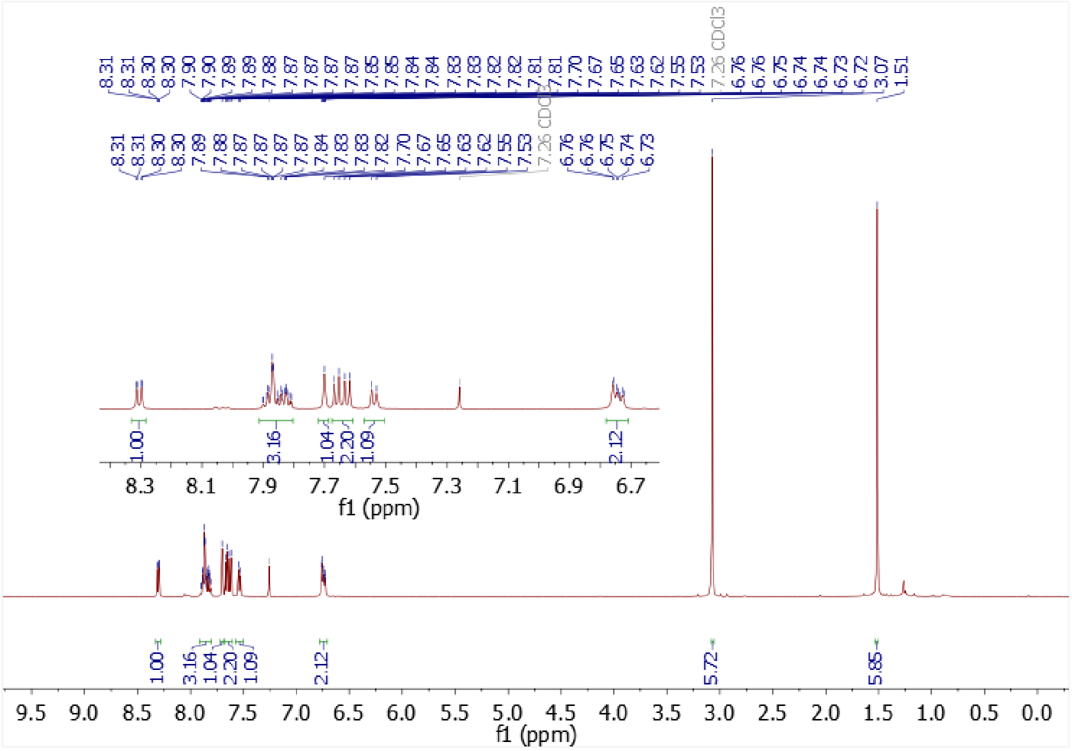

Figure S15. ^{1}H-NMR spectrum of **4** in $CDCl_3$.

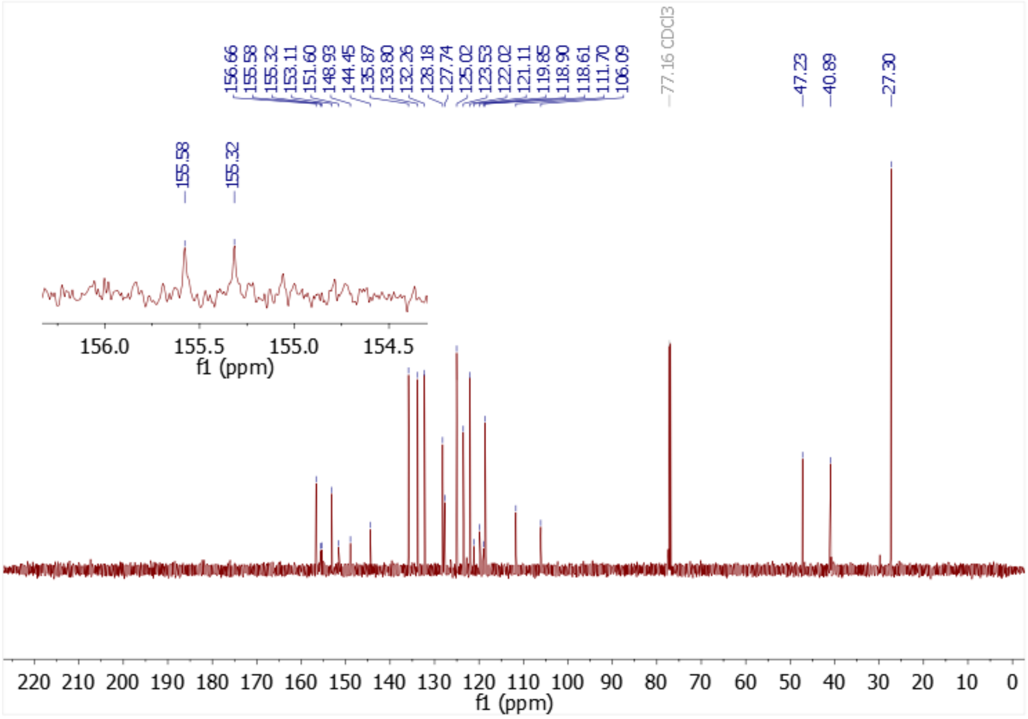

Figure S16. ^{13}C-NMR spectrum of **4** in $CDCl_3$.

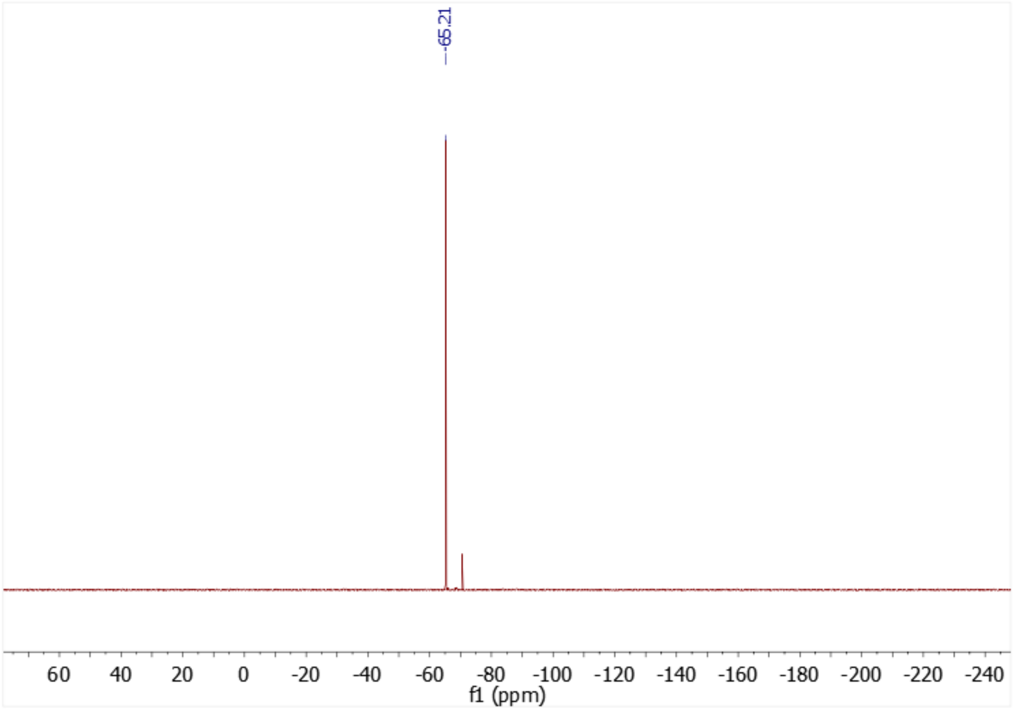

Figure S17. ^{19}F-NMR spectrum of **4** in $CDCl_3$.

Synthesis of N,N,9,9-tetramethyl-7-(3-(trifluoromethyl)diaziridin-3-yl)-9H-fluoren-2-amine (5):

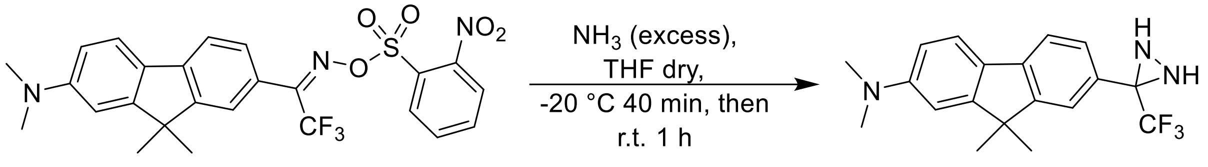

A solution of **4** (656.7 mg, 1.2 mmol) in 15 mL of THF was prepared in a 50 mL round bottom flask, a magnet stirrer was added to the solution, the flask was cooled down at -20 °C and the neck was sealed with a rubber septum. Anhydrous ammonia was bubbled in the solution for 40 min, then the mixture was allowed to reach room temperature and was left under stirring for 1 h. The mixture obtained was quenched with NH_4Cl and the aqueous phase was extracted with Et_2O three times. The combined organic phases were dried over Na_2SO_4 and concentrated under vacuum. The crude was finally purified by flash chromatography (70% pentane, 30% Et_2O) affording pure diaziridine **5** (367.4 mg, 88% yield) as an orange solid. Melting point: 131-133 °C. ^{1}H-NMR (500 MHz, $CDCl_3$) δ(ppm): 7.63-7.56 (m, 3H, ArH), 7.52 (d, *J*= 8.0 Hz, 1H, ArH), 6.76 (d, *J*= 2.4 Hz, 1H, ArH), 6.73 (dd, *J*= 8.4, 2.3 Hz ,1H, ArH), 3.05 (s, 6H, NCH_3), 2.81 (d, *J*= 8.6 Hz, 1H, NH), 2.28 (d, *J*= 8.7 Hz, 1H, NH), 1.48 (s, 6H, CH_3). ^{13}C-NMR (500 MHz, $CDCl_3$) δ(ppm): 155.87, 153.36, 151.14, 142.07, 128.17, 127.18, 127.05, 123.87 (q= 278.3 Hz), 122.06, 121.36, 118.62, 111.63, 106.37, 58.47 (q= 35.8 Hz), 47.04, 40.98, 27.55 (d, *J*= 2.3 Hz). ^{19}F-NMR (283 MHz, $CDCl_3$) δ(ppm): −75.50. IR (ν): 684.0, 713.8, 739.9, 775.3, 805.1, 844.2, 890.8, 913.2, 961.7, 1004.5, 1071.6, 1099.6, 1149.9, 1164.8, 1215.1, 1274.7, 1306.4, 1358.6, 1412.7, 1435.0, 1483.5, 1507.7, 1578.5, 1612.1, 1694.1, 2803.0, 2905.5, 2924.1, 2961.4, 3015.4, 3039.6, 3227.9, 3255.8. HRMS (ESI+) m/z $[MH]^+$ calculated for $C_{19}H_{21}F_3N_3$: 348.1682, found: 348.1677.

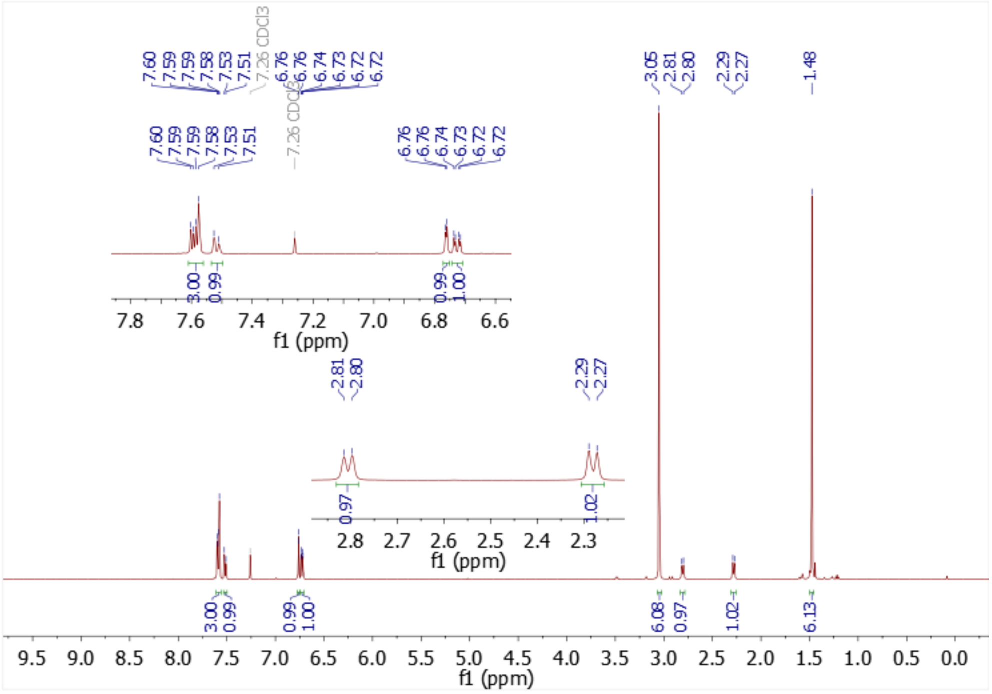

Figure S18. ^{1}H-NMR spectrum of **5** in $CDCl_3$.

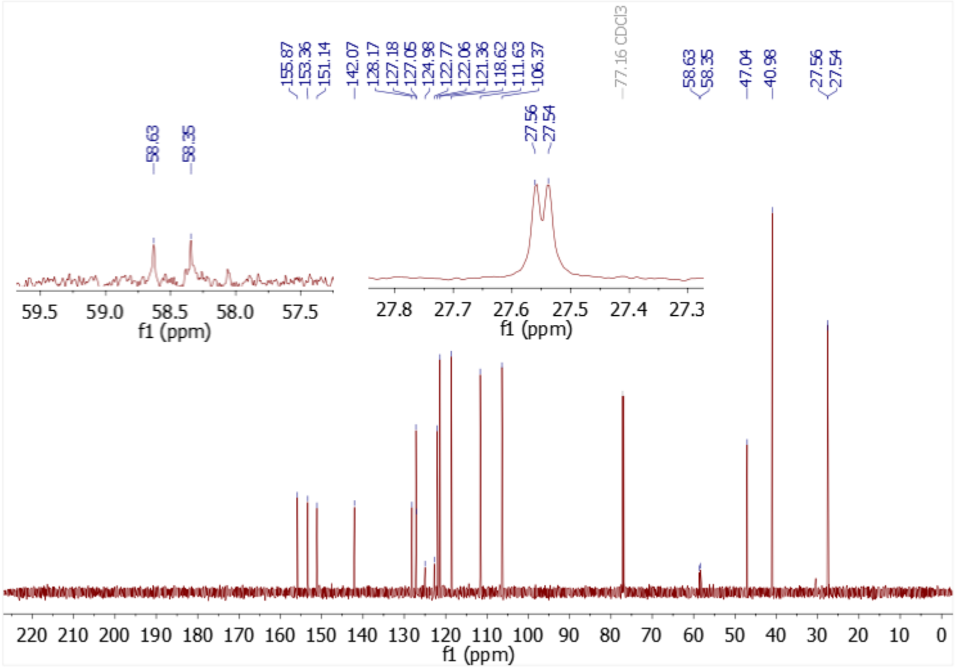

Figure S19. ^{13}C-NMR spectrum of **5** in $CDCl_3$.

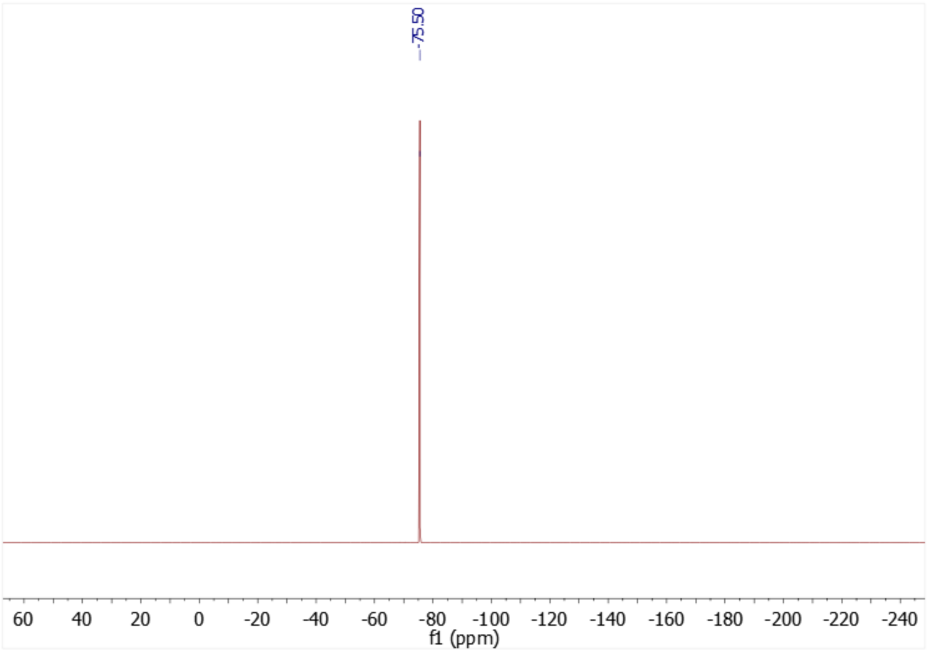

Figure S20. ^{19}F-NMR spectrum of **5** in $CDCl_3$.

Synthesis of N,N-9,9-tetramethyl-7-(3-(trifluoromethyl)-3H-diazirin-3-yl)-9H-fluoren-2-amine (1):

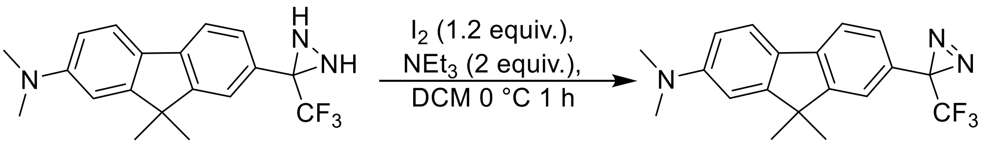

A solution of **5** (424.1 mg, 1.2 mmol) in 6 mL of DCM was prepared in a 25 mL round bottom flask and was cooled at 0 °C. Subsequently, I_2 (370.8 mg, 1.4 mmol) and NEt_3 (0.3mL, 2.4 mmol) were added to the solution and the mixture obtained was left under stirring for 1 h before being quenched with a saturated solution of $Na_2S_2O_3$. The aqueous phase was extracted three times with DCM and the combined organic phases were washed with brine, dried over Na_2SO_4, and concentrated under vacuum. The crude obtained was purified by flash chromatography (80% pentane, 20% Et_2O) affording **1** (390 mg, 93% yield) as an orange solid. ^{1}H-NMR (500 MHz, $CDCl_3$) δ(ppm): 7.57 (t, *J*= 9.0 Hz, 2H, ArH), 7.14 (d, *J*= 7.9 Hz, 2H, ArH), 6.75 (d, *J*= 2.4 Hz, 1H, ArH), 6.72 (dd, *J*= 8.4, 2.4 Hz ,1H, ArH), 3.05 (s, 6H, NCH_3), 1.45 (s, 6H, CH_3). ^{13}C-NMR (500 MHz, $CDCl_3$) δ(ppm): 155.85, 153.53, 151.23, 141.77, 126.78, 125.74, 125.55, 123.21 (q, *J*= 123.62 Hz), 121.44, 120.40, 118.79, 111.67, 106.30, 47.03, 40.95, 29.07 (q, *J*= 40.4 Hz), 27.48. ^{19}F-NMR (283 MHz, $CDCl_3$) δ(ppm): −65.12. $\lambda_{max\ (MeOH)}$= 358 nm, $\lambda_{abs\ (MeOH)}$= 415 nm. IR(ν): 723.1, 807.0, 838.7, 930.0, 963.5, 978.4, 1049.2,

1071.6, 1155.5, 1176.0, 1203.9, 1222.6, 1258.0, 1295.2, 1336.3, 1358.6, 1435.0, 1485.3, 1507.7, 1576.7, 1608.3, 2801.1, 2864.5, 2898.0, 2927.8, 2965.1. HRMS (ESI+) m/z [MH]+ calculated for $C_{19}H_{19}F_{3}N_{3}$: 346.1526, found: 346.1521.

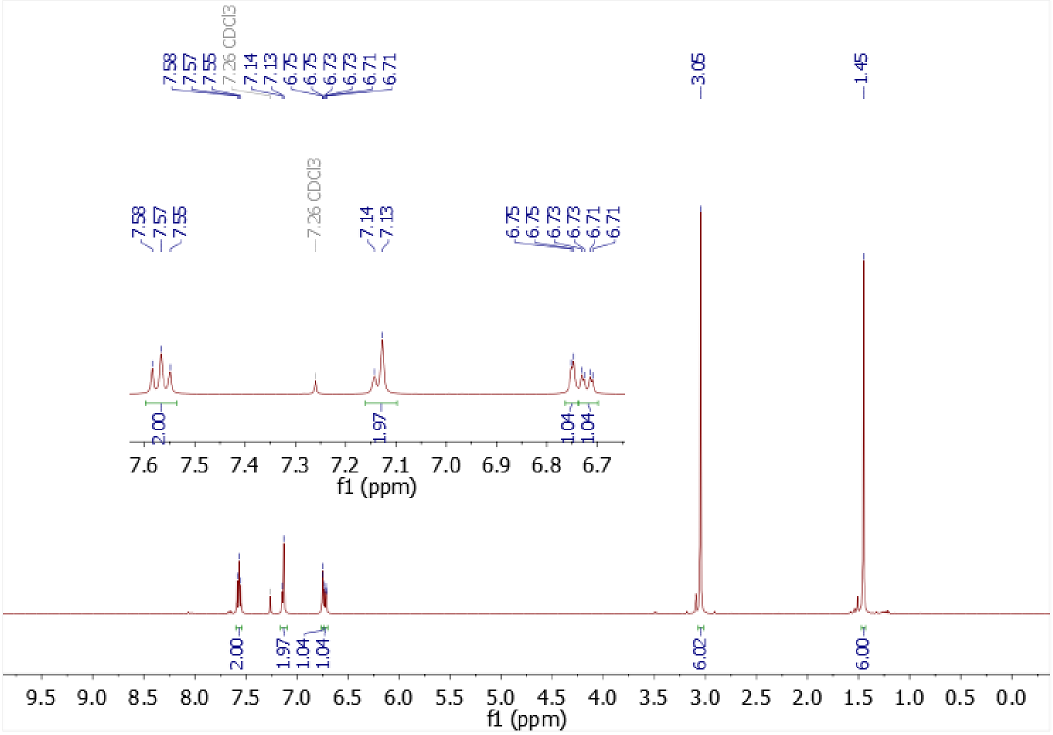

Figure S21. ^{1}H-NMR spectrum of **1** in $CDCl_3$.

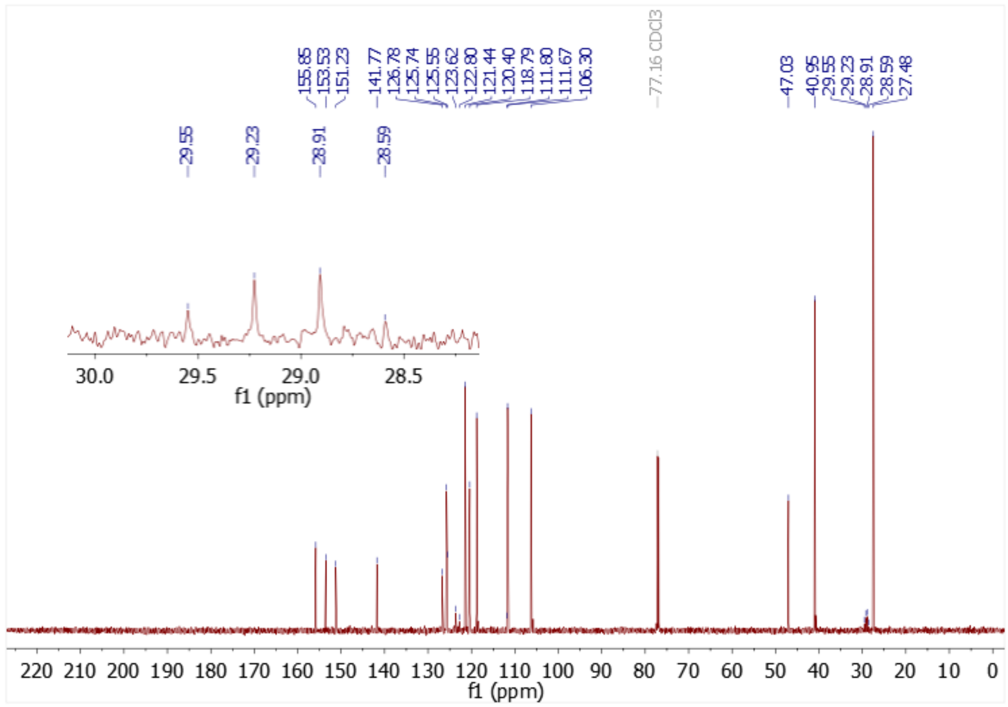

Figure S22. ^{13}C-NMR spectrum of **1** in $CDCl_3$.

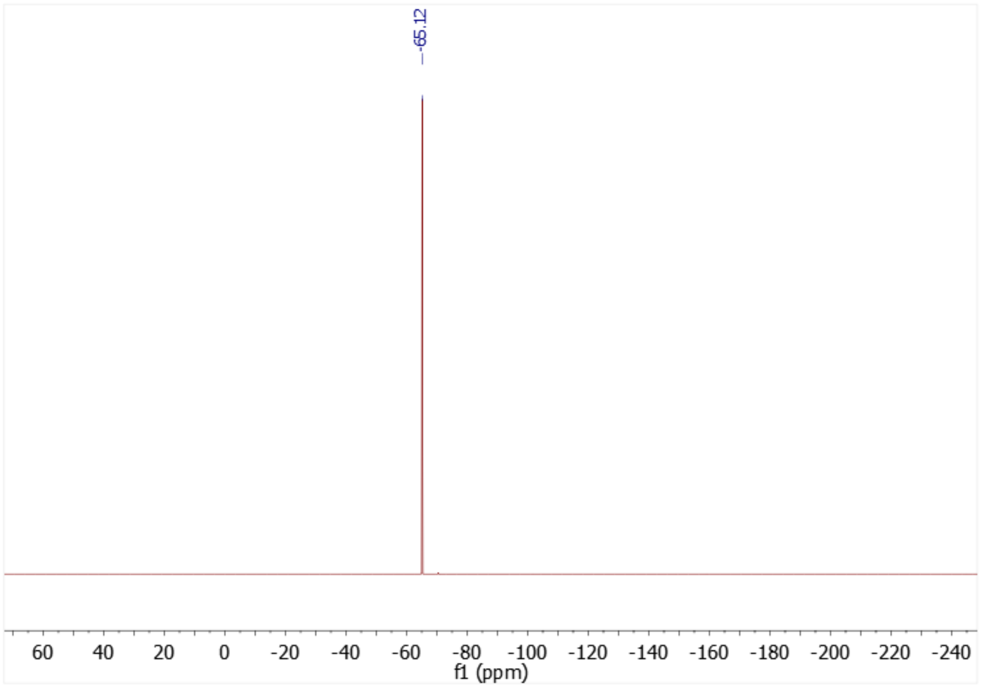

Figure S23. ^{19}F-NMR spectrum of **1** in $CDCl_3$.

Synthesis of Control Diazirine 1':

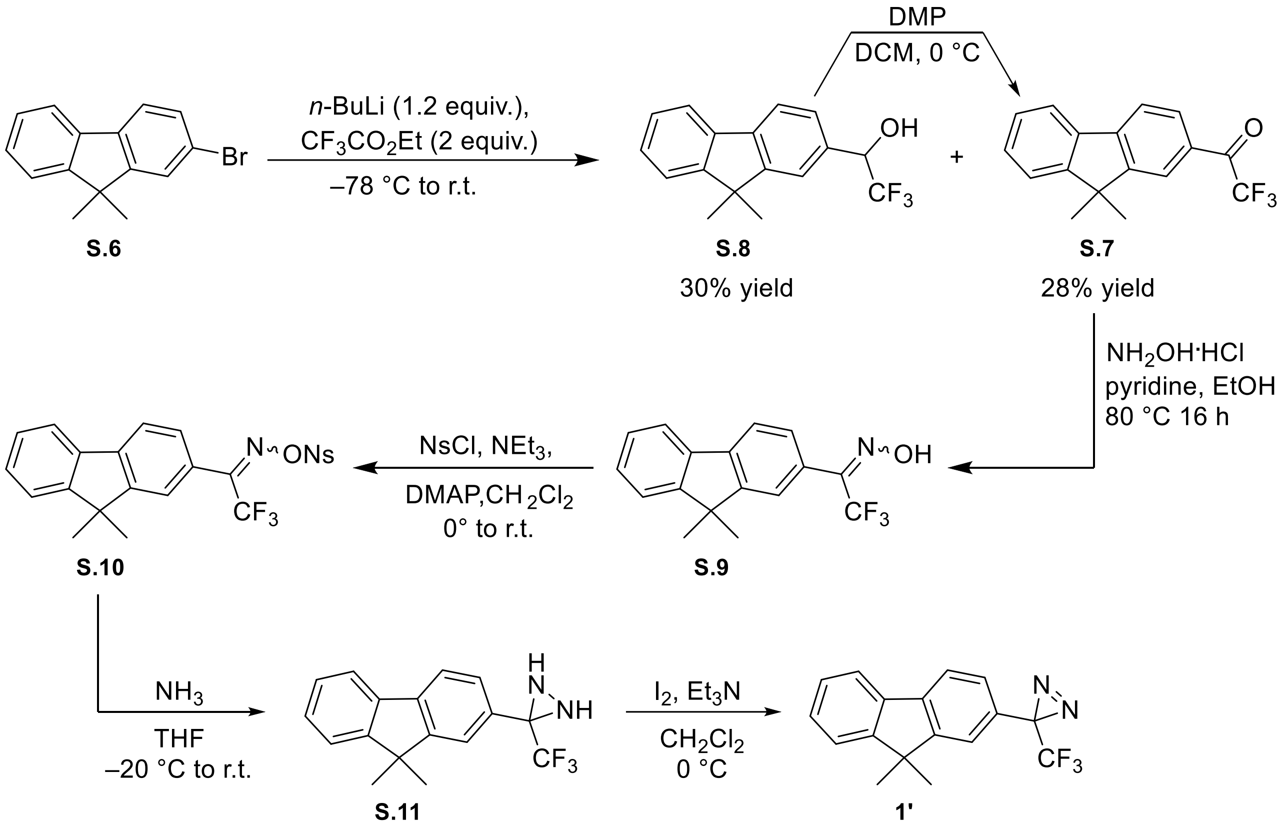

Figure S24. Synthetic scheme for the production of control diazirine **1'**.

Synthesis of 1-(9,9-dimethyl-9H-fluoren-2-yl)-2,2,2-trifluoroethan-1-one (S.7) and 1-(9,9-dimethyl-9H-fluoren-2-yl)-2,2,2-trifluoroethan-1-ol (S.8):

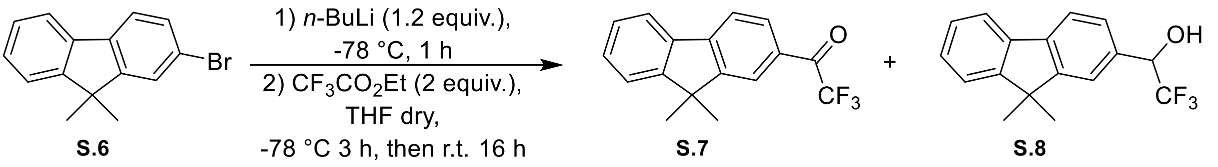

A solution of commercially available 2-bromo-9,9-dimethylfluorene (**S.6**, 10 g, 36.6 mmol) in 100 mL of dry THF was prepared in a 250 mL flame-dried two-neck round bottom flask and cooled at −78 °C. Under argon atmosphere, *n*-butyl lithium 2.5 M in *n*-hexane (17.6 mL, 43.9 mmol) was added dropwise to the solution. The mixture obtained was left under stirring at −78 °C for 1 h and ethyl trifluoroacetate (8.7 mL, 73.2 mmol) was added dropwise to the mixture that was left under stirring at −78 °C for 3 h. After this time the reaction was allowed to warm up at room temperature slowly, and after 16 h stirring was quenched with a saturated solution of NH_4Cl. The aqueous phase was extracted with EtOAc, dried over $MgSO_4$, and concentrated under vacuum. The crude product mixture was purified by flash-column chromatography (90% hexanes, 10% CH_2Cl_2) to afford **S.7** (3 g, 28% yield) as an off-white solid and **S.8** (3.2 g, 30% yield) as an off-white solid.

Characterization data for **S.7**: ^{1}H-NMR (300 MHz, $CDCl_3$) δ(ppm): 8.14 (apparent d, J = 0.8 Hz, 1H, ArH), 8.12-8.05 (m, 1H, ArH), 7.88-7.80 (m, 2H, ArH), 7.49 (ddd, J = 6.4, 5.8, 1.6 Hz, 1H, ArH), 7.46-7.37 (m, 2H, ArH), 1.54 (s, 6H, CH_3). ^{19}F-NMR (283 MHz, $CDCl_3$) δ(ppm): −70.82.

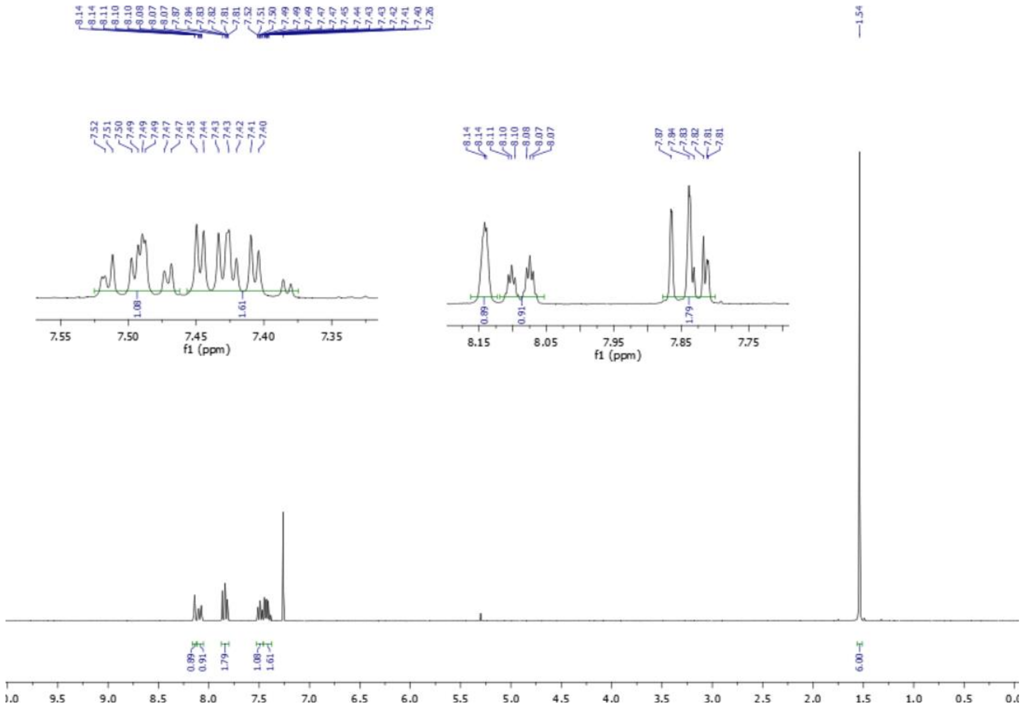

Figure S25. ^{1}H-NMR spectrum of **S.7** in $CDCl_3$.

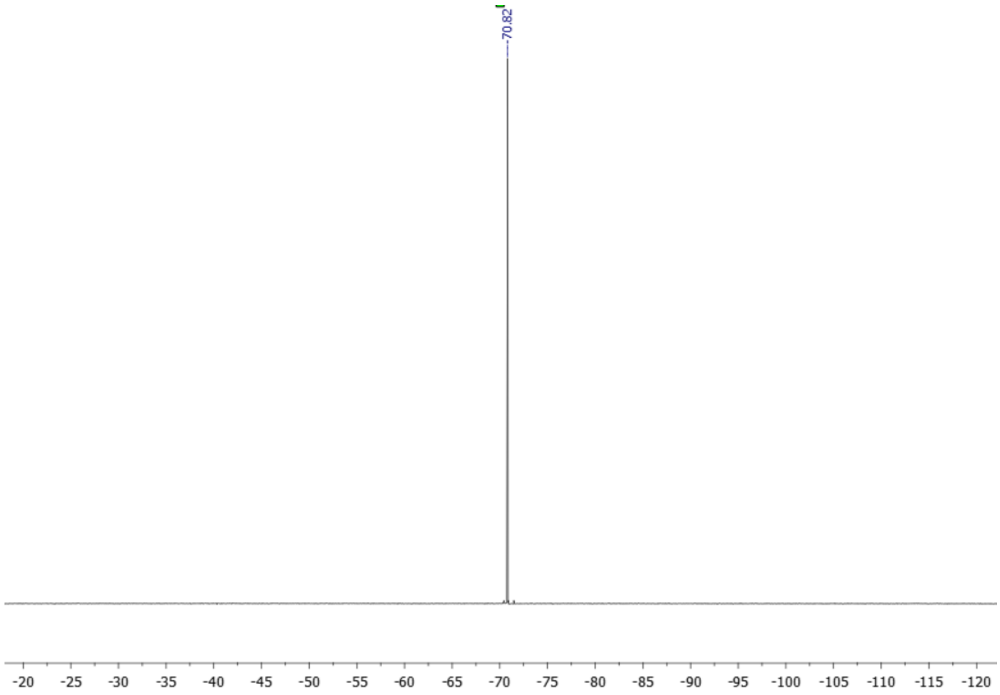

Figure S26. ^{19}F-NMR spectrum of **S.7** in $CDCl_3$.

Characterization data for **S.8**: ^{1}H-NMR (300 MHz, $CDCl_3$) δ(ppm): 7.74 (dd, J = 8.3, 2.4 Hz, 2H, ArH), 7.55 (s, 1H, ArH), 7.49-7.41 (m, 2H, ArH), 7.35 (dt, J = 4.8, 4.1 Hz, 2H, ArH), 5.16-5.05 (m, 1H, ArCH), 2.65-2.49 (m, 1H, OH), 1.52 (s, 6H, CH_3). ^{19}F-NMR (283 MHz, $CDCl_3$) δ(ppm): −78.22.

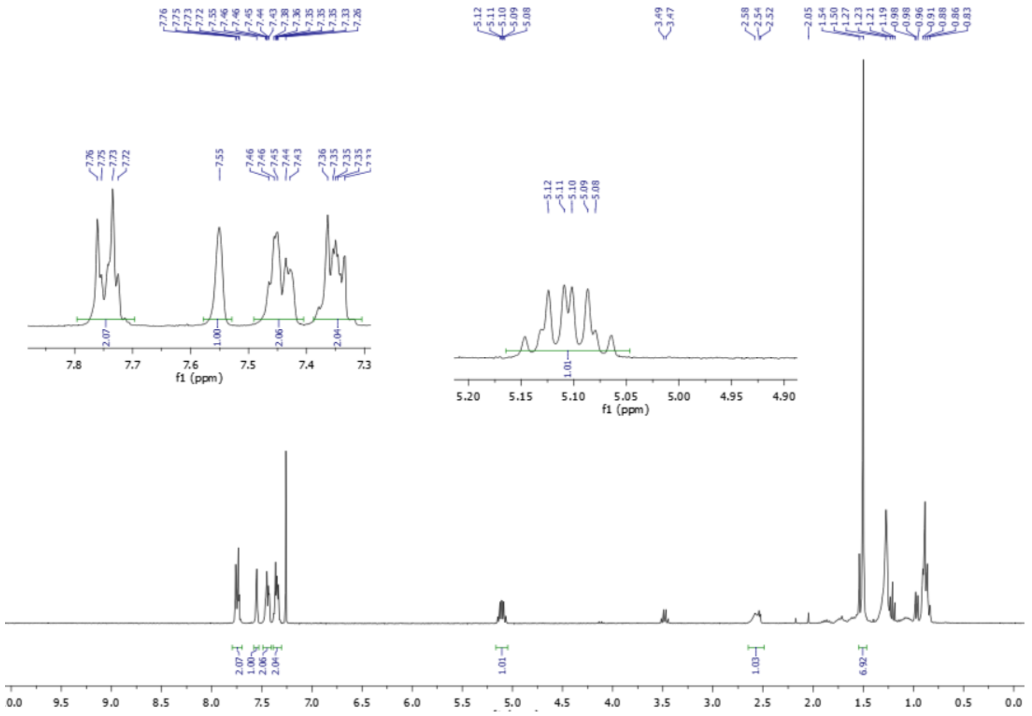

Figure S27. ^{1}H-NMR spectrum of **S.8** in $CDCl_3$.

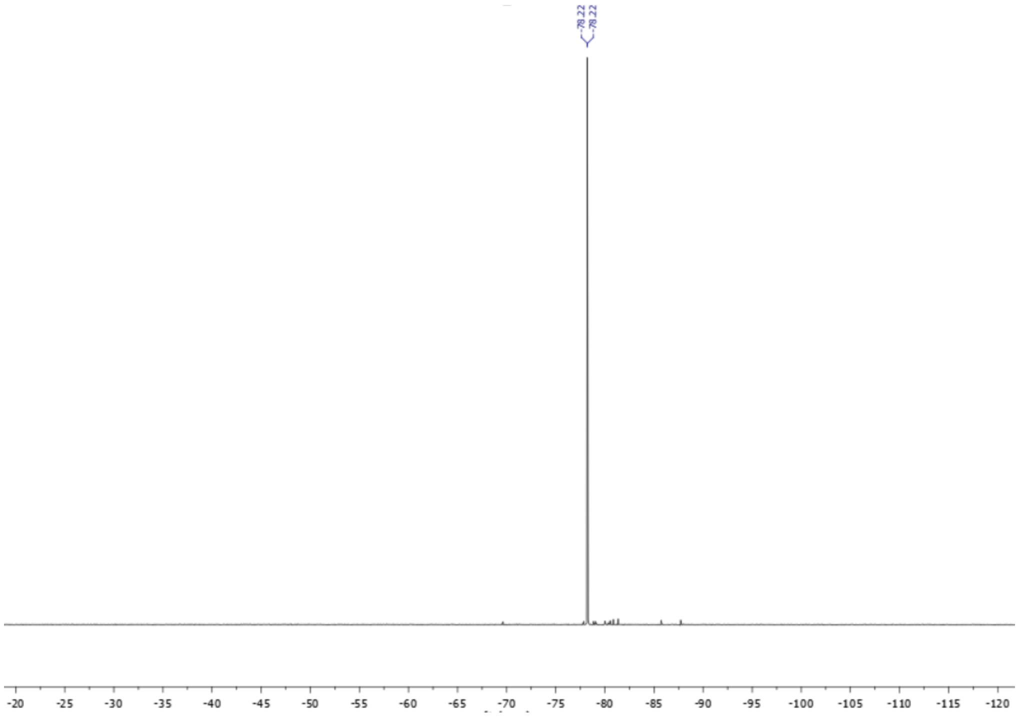

Figure S28. ^{19}F-NMR spectrum of **S.8** in $CDCl_3$.

Conversion of 1-(9,9-dimethyl-9H-fluoren-2-yl)-2,2,2-trifluoroethan-1-ol (S.8) to 1-(9,9-dimethyl-9H-fluoren-2-yl)-2,2,2-trifluoroethan-1-one (S.7)

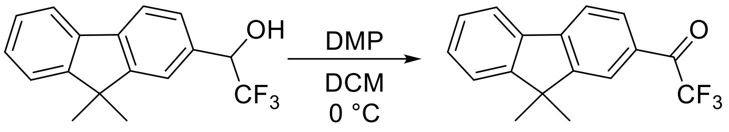

A solution of alcohol **S.8** (200 mg, 0.68 mmol) in DCM (5 mL) was cooled to 0 °C, and DMP (432 mg, 1.02 mmol) was added to the reaction mixture. The reaction was stirred overnight at 0 °C and was monitored by TLC. After complete consumption of starting material, saturated $NaHCO_3$ was added and the mixture was extracted three times with DCM. The combined organic phases were dried over $MgSO_4$, and finally concentrated under vacuum to obtain crude product **S.7**. The product identity was confirmed by ^{19}F NMR.

Synthesis of 1-(9,9-dimethyl-9H-fluoren-2-yl)-2,2,2-trifluoroethan-1-one oxime (S.9):

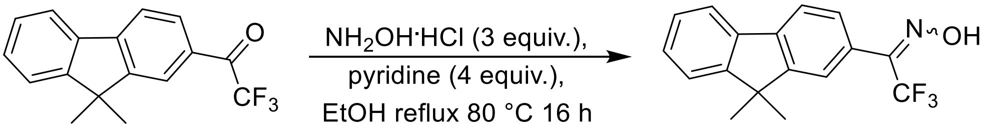

A solution of **S.7** (1 g, 3.4 mmol) in 15 mL of absolute EtOH was prepared in a 25 mL round bottom flask. Under stirring, pyridine (1.1 mL, 13.6 mmol) and hydroxylamine hydrochloride (0.7 g, 10.2 mmol) were added to the solution. The mixture obtained was heated under reflux at 80 °C for 16 h, then was treated with a 1M solution of HCl and the aqueous phase was extracted several times with Et_2O. The combined organic phases were washed with water until reaching pH 7, dried over $MgSO_4$, and finally concentrated under vacuum. The crude product **S.9**, obtained as a mixture of two geometrical isomers, was submitted to the next step without further purification.

^1H-NMR (300 MHz, $CDCl_3$) δ(ppm): 8.75 (s, 1H, OH), 8.46 (s, 1H, OH), 7.80 (dd, J = 7.9, 0.6 Hz, 1H, ArH), 7.78-7.72 (m, 3H, ArH), 7.59-7.53 (m, 2H, ArH), 7.53-7.43 (m, 4H, ArH), 7.39-7.34 (m, 4H, ArH), 1.52-1.49 (m, 12H, CH_3). ^{19}F-NMR (283 MHz, $CDCl_3$) δ(ppm): −62.12 major isomer, −66.2 minor isomer.

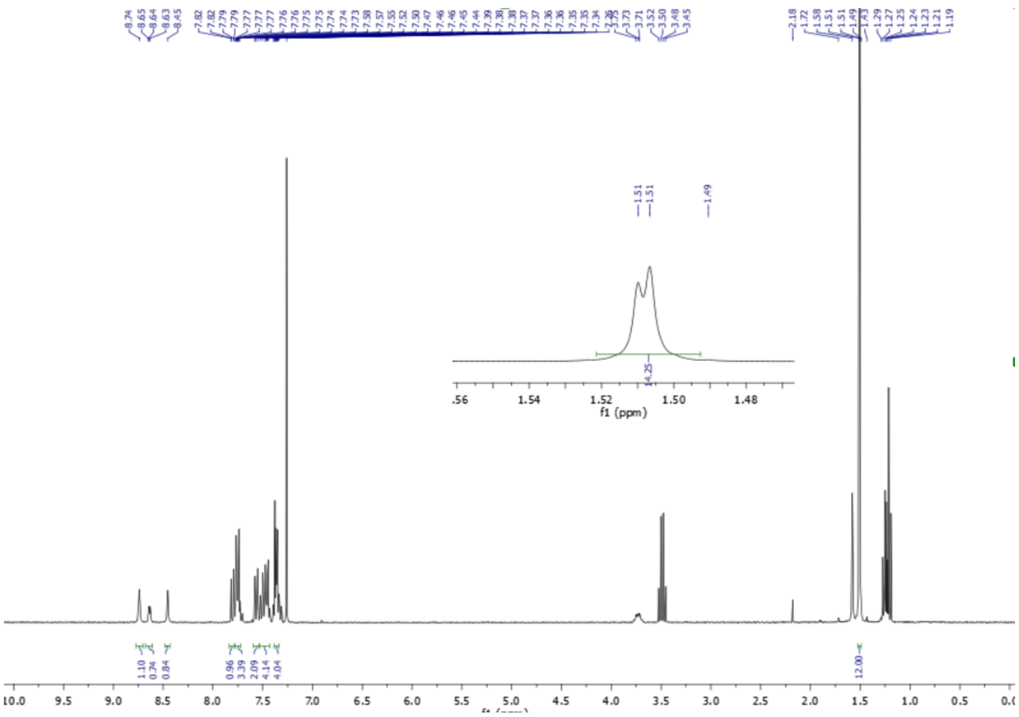

Figure S29. ^{1}H-NMR spectrum of **S.9** in $CDCl_3$.

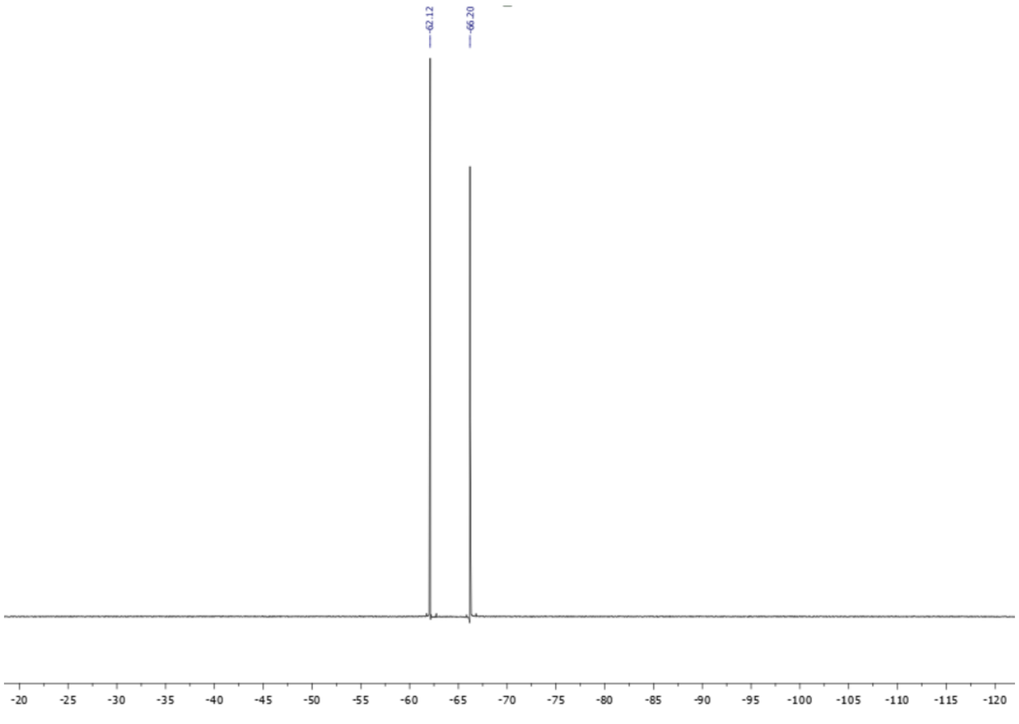

Figure S30. ^{19}F-NMR spectrum of **S.9** in $CDCl_3$.

Synthesis of 1-(9,9-dimethyl-9H-fluoren-2-yl)-2,2,2-trifluoroethan-1-one O-((2-nitrophenyl) sulfonyl) oxime (S.10):

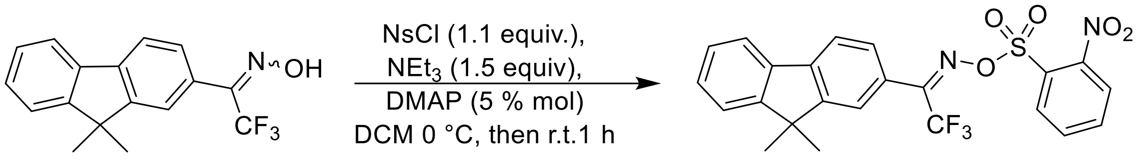

S.9 **S.10**

A solution of **S.9** (1.00 g, 3.27 mmol) in 20 mL of DCM was prepared in a 50 mL round bottom flask. The solution was cooled at 0 °C and NEt_3 (0.68 mL, 4.9 mmol), DMAP (0.02 g, 0.16 mmol), and 2-nitrobenzensulfonyl chloride (797 mg, 3.6 mmol) were added in this order. After 5 min, the mixture obtained was allowed to reach room temperature and was left under stirring for 1 h. Subsequently, the mixture was treated with NH_4Cl reaching approximately pH 6, and the aqueous phase was extracted with Et_2O three times. The combined organic phases were dried over $MgSO_4$ and concentrated under vacuum. The crude product **S.10** obtained was submitted to the next step without further purification.

^{1}H-NMR (300 MHz, $CDCl_3$) δ(ppm): 8.31 (ddd, *J* = 7.5, 1.4, 0.6 Hz, 1H), 8.27-8.21 (m, 1H), 7.95-7.71 (m, 13H), 7.56 (dd, *J* = 8.0, 0.8 Hz, 1H), 7.53-7.31 (m, 10H), 1.54 (s, 6H), 1.48 (s, 7H). ^{19}F-NMR (283 MHz, $CDCl_3$) δ(ppm): −61.05 major isomer, −65.74 minor isomer.

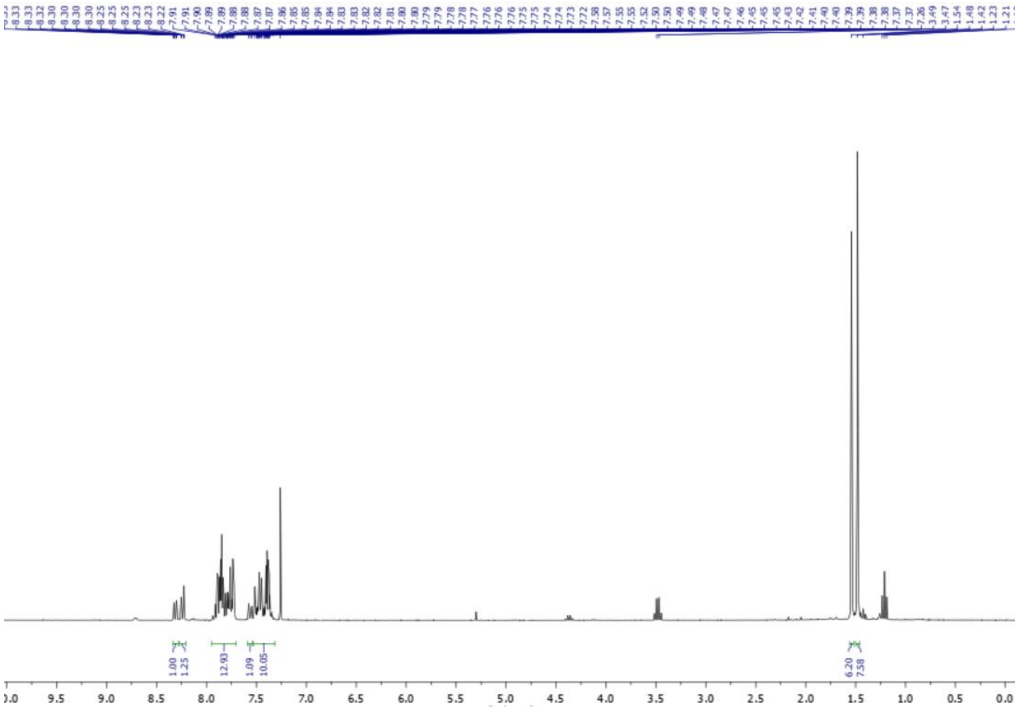

Figure S31. ^{1}H-NMR spectrum of **S.10** in $CDCl_3$.

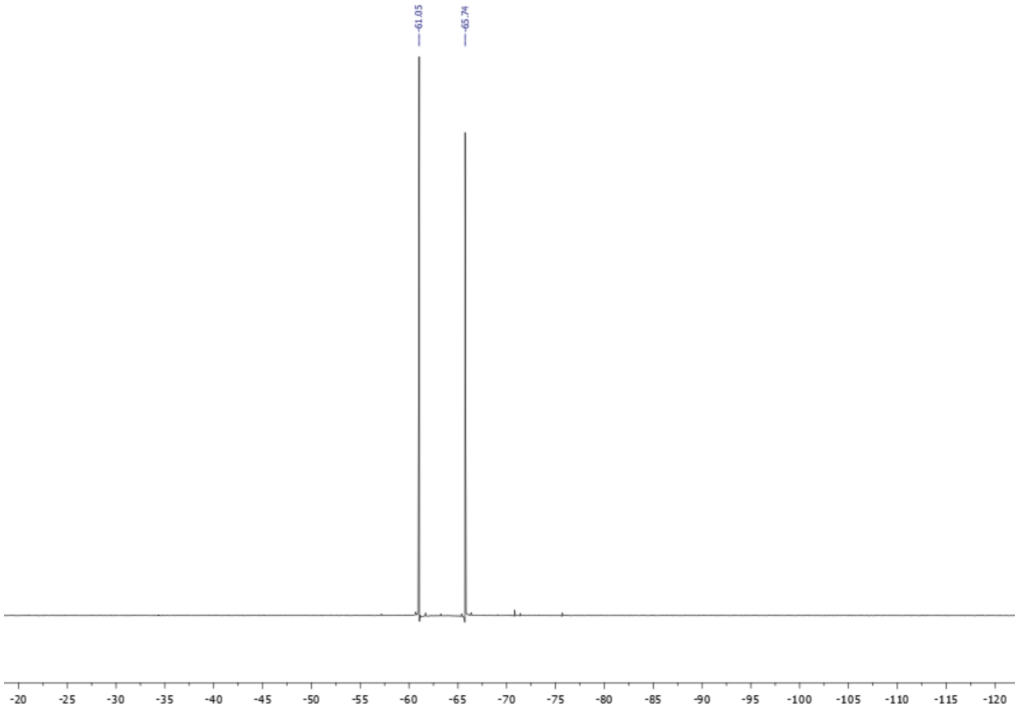

Figure S32. ^{19}F-NMR spectrum of **S.10** in $CDCl_3$.

Synthesis of 3-(9,9-dimethyl-9H-fluoren-2-yl)-3-(trifluoromethyl)diaziridine (S.11):

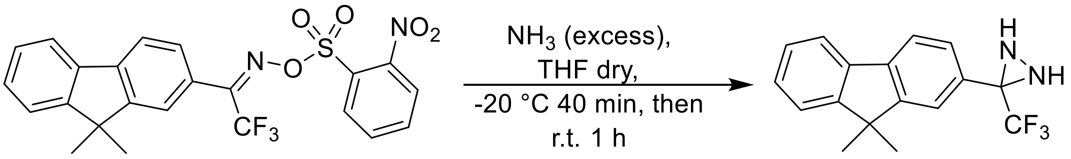

S.10 **S.11**

A solution of crude **S.10** (1.6 g, 3.27 mmol) in 25 mL of THF was prepared in a 50 mL round bottom flask, a magnet stirrer was added to the solution, the flask was cooled at –78 °C and the neck was sealed with a rubber septum. Anhydrous ammonia was bubbled in the solution for 40 min, then the mixture was allowed to reach room temperature and was left under stirring for 1 h. The mixture obtained was quenched with NH_4Cl and the aqueous phase was extracted with Et_2O three times. The combined organic phases were dried over $MgSO_4$ and concentrated under vacuum and the crude product **S.11** used in the next step without purification.

^{1}H-NMR (300 MHz, $CDCl_3$) δ(ppm): 7.78-7.71 (m, 2H), 7.66 (s, 1H), 7.59 (dd, J = 7.9, 0.9 Hz, 1H), 7.45 (ddd, J = 7.5, 3.8, 2.3 Hz, 1H), 7.39-7.32 (m, 2H), 2.83 (d, J = 8.0 Hz, 1H), 2.29 (d, J = 8.9 Hz, 1H), 1.50 (s, 6H). ^{19}F-NMR (283 MHz, $CDCl_3$) δ(ppm): −75.45.

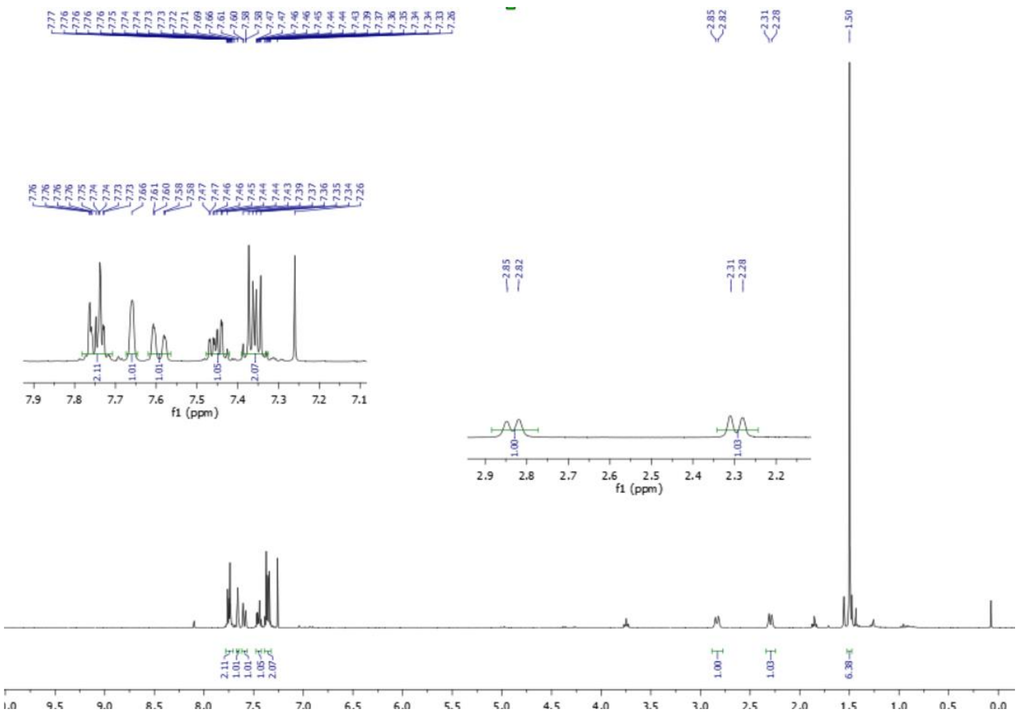

Figure S33. ^{1}H-NMR spectrum of **S.11** in $CDCl_3$.

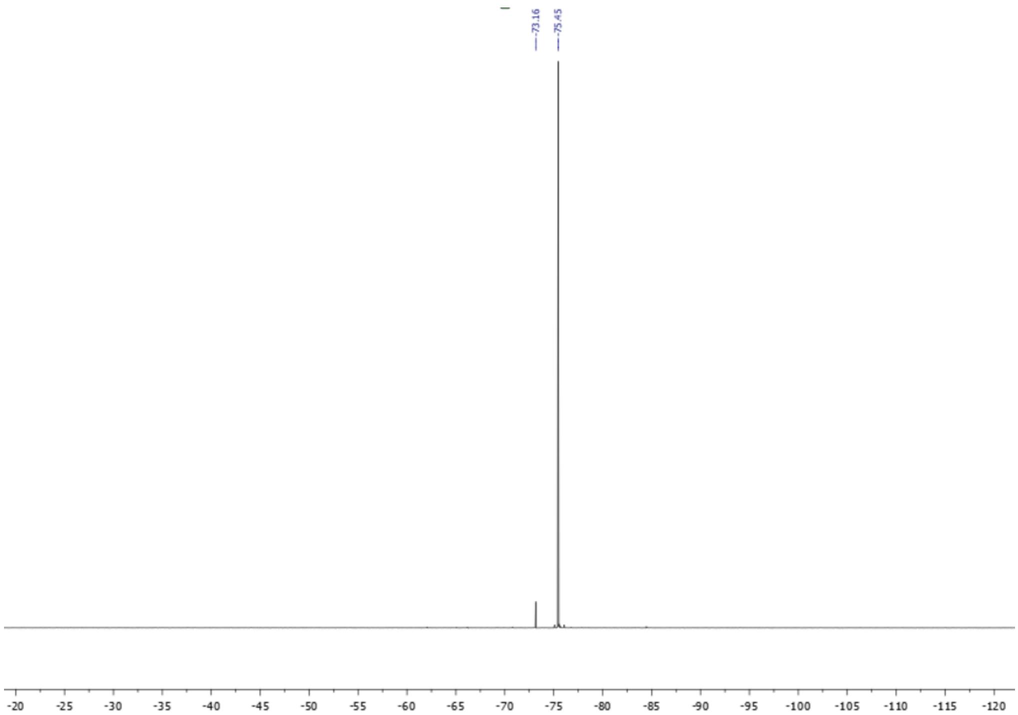

Figure S34. ^{19}F-NMR spectrum of **S.11** in $CDCl_3$.

Synthesis of 3-(9,9-dimethyl-9H-fluoren-2-yl)-3-(trifluoromethyl)-3H-diazirine (1'):

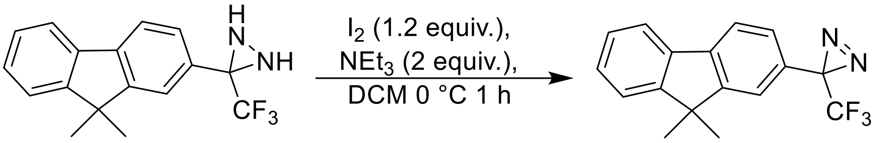

S.11 **1'**

A solution of crude **S.11** (1.00 g, 3.27 mmol) in 20 mL of DCM was prepared in a 50 mL round bottom flask and was cooled at 0 °C. Subsequently, I_2 (995 mg, 3.92 mmol) and NEt_3 (0.91 mL, 6.54 mmol) were added to the solution and the mixture obtained was left under stirring for 1 h before being quenched with a saturated solution of $Na_2S_2O_3$. The aqueous phase was extracted three times with DCM and the combined organic phases were washed with brine, dried over $MgSO_4$, and concentrated under vacuum. The crude obtained was purified by flash chromatography (90% hexane, 10% Et_2O) affording **1'** (873 mg, 88% yield) as a white solid.

^{1}H-NMR (300 MHz, $CDCl_3$) δ(ppm): 7.76-7.69 (m, 2H, ArH), 7.48-7.41 (m, 1H, ArH), 7.39-7.32 (m, 2H, ArH), 7.23-7.17 (m, 2H, ArH), 1.47 (s, 6H, CH_3). ^{19}F-NMR (283 MHz, $CDCl_3$) δ(ppm): −65.07. ^{13}C-NMR (500 MHz, $CDCl_3$) δ(ppm): 154.19, 153.90, 140.87, 137.86, 128.18, 127.76, 127.23, 125.64, 122.29 (q, *J*= 147.5 Hz), 122.74, 120.66, 120.53, 120.29, 47.05, 28.87 (q, *J*= 40 Hz), 26.96

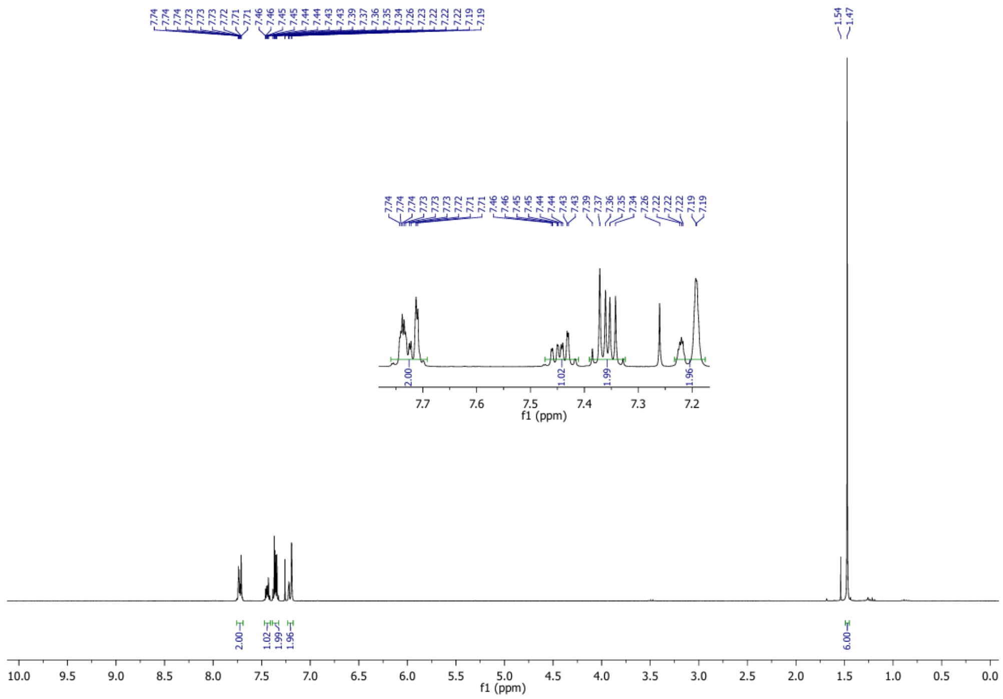

Figure S35. ^{1}H-NMR spectrum of **1'** in $CDCl_3$.

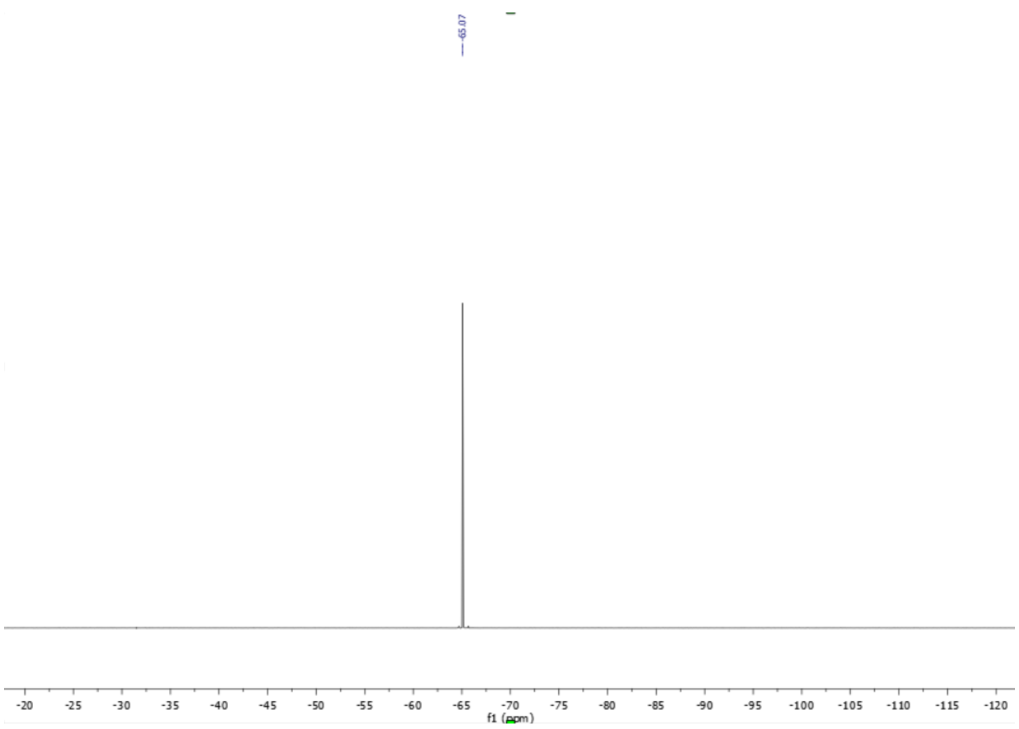

Figure S36. ^{19}F-NMR spectrum of **1'** in $CDCl_3$.

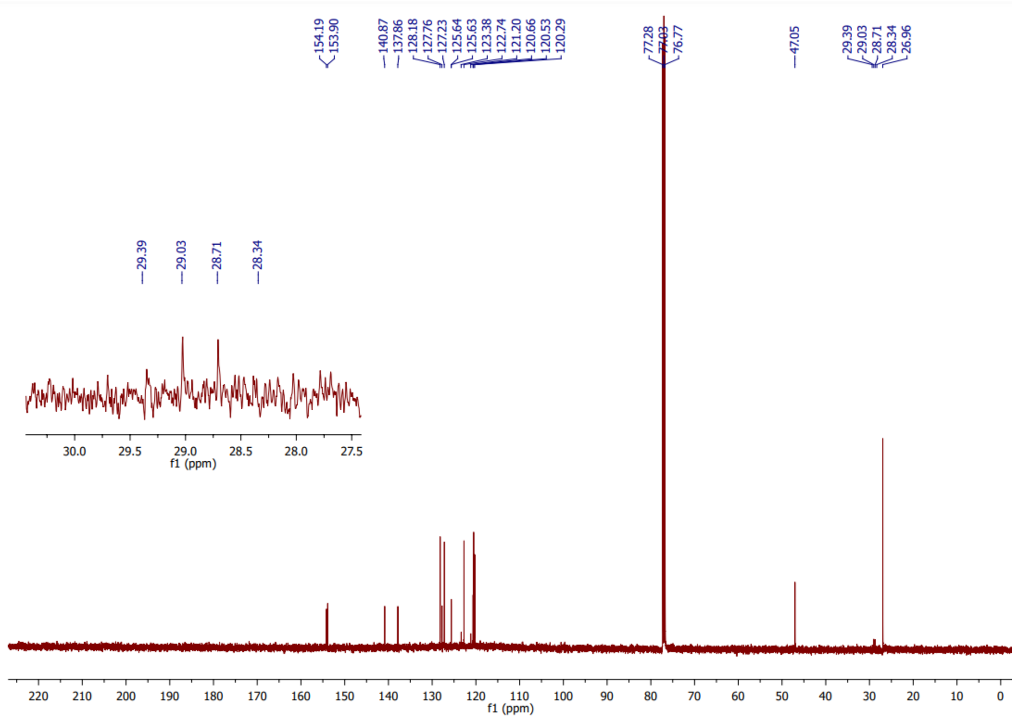

Figure S37. ^{13}C-NMR spectrum of **1'** in $CDCl_3$.

Preparative Scale C–H Insertion Reactions:

Photochemical insertion in cyclohexane with visible light (blue, 460 nm):

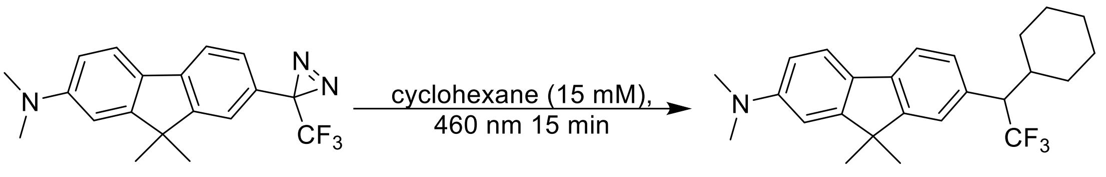

1 **7**

A solution of **1** (18.7 mg, 0.05 mmol) in cyclohexane (15 mM) was prepared in a vial that was subsequently sealed with a septum and degassed with argon for 15 min. The vial was placed in a photochemical chamber with an air-cooling system and the light (blue, 460 nm) was turned on. The solution was stirred for 15 min under irradiation of blue light. The mixture was then concentrated under vacuum and the product obtained was purified by flash chromatography (80% pentane, 20% Et_2O) affording **7** (13.1 mg, 60% yield) as an orange solid. Melting point: 45-50 °C. ^{1}H-NMR (500 MHz, $CDCl_3$) δ(ppm): 7.56 (d, *J*= 8.4 Hz, 1H, ArH), 7.53 (d, *J*= 7.8 Hz, 1H, ArH), 7.20 (s, 1H, ArH), 7.14 (d, *J*= 7.8 Hz ,1H, ArH), 6.78 (d, *J*= 2.4 Hz, 1H, ArH), 6.72 (dd, *J*= 8.4, 2.4 Hz, 1H, ArH), 3.10-30.7 (m, 1H, CF_3CH), 3.04 (s, 6H, NCH_3), 2.03-1.95 (m, 2H, CH_2), 1.81-1.75 (m, 1H, CH), 1.67-1.60 (m, 2H, CH_2), 1.57 (d, *J*= 12.9 Hz, 1H, CH_2), 1.47, (d, *J*= 3.2 Hz, 6H, CH_3), 1.33 (dt, *J*= 13.0, 3.6 Hz, 1H, CH_2), 1.30-1.27 (m, 1H, CH_2), 1.20-1.08 (m, 3H, CH_2). 0.91-0.82 (m, 1H, CH_2). ^{1}H-NMR (500 MHz, CD_3OD) δ(ppm): 7.56 (d, *J*= 8.4 Hz, 1H, ArH), 7.53 (d, *J*= 7.8 Hz, 1H, ArH), 7.26 (s, 1H, ArH), 7.14 (d, *J*= 8.6 Hz, 1H, ArH), 6.87 (d, *J*= 2.4 Hz, 1H, ArH), 6.76 (dd, *J*= 8.4, 2.4 Hz, 1, ArH), 3.20 (m, 1H, CF_3CH), 3.00 (s, 6H, NCH_3), 2.04-1.96 (m, 2H, CH_2), 1.81-1.77 (m, 1H, CH), 1.66-1.61 (m, 2H, CH_2), 1.53 (d, *J*= 7.8 Hz, 1H, CH_2), 1.44 (d, *J*= 4.5 Hz,), 1.37-1.33 (m, 1H, CH_2), 1.32-1.29 (m, 1H, CH_2), 1.22-1.19 (m, 3H, CH_2), 0.94-0.86 (m, 1H, CH_2). ^{13}C-NMR (500 MHz, $CDCl_3$) δ(ppm): 155.50, 153.27, 150.75, 139.58, 132.10, 128.73, 127.94, 126.08 (d, *J*= 103.2 Hz), 123.31, 120.83, 118.44, 111.65, 106.71, 56.40 (q, *J*= 24.9 Hz), 46.84, 41.10, 38.86, 31.75, 30.90, 27.67, 27.63, 26.40, 26.31, 26.21. ^{13}C-NMR (500 MHz, CD_3OD) δ(ppm): 156.56, 154.50, 152.48, 140.85, 133.38, 129.34, 129.01, 127.0 (d, *J*= 219.4 Hz), 124.39, 121.63, 119.31, 113.25, 108.21, 57.10 (q, *J*= 24.6 Hz), 47.69, 41.39, 40.05, 32.70, 31.92, 27.86, 27.84, 27.29, 27.21, 27.18. ^{19}F-NMR (283 MHz, $CDCl_3$) δ(ppm): −63.26. ^{19}F-NMR (283 MHz, CD_3OD) δ(ppm): −64.56. IR (ν): 807.0, 844.2, 963.5, 1099.6, 1153.6, 1176.0, 1218.8, 1252.4, 1291.5, 1354.9, 1435.0, 1448.1, 1474.2, 1504.0, 1578.5, 1612.1, 2806.7, 2855.1, 2924.1. HRMS (ESI+) m/z $[MH]^+$ calculated for $C_{25}H_{31}F_3N$: 402.2402, found: 402.2399.

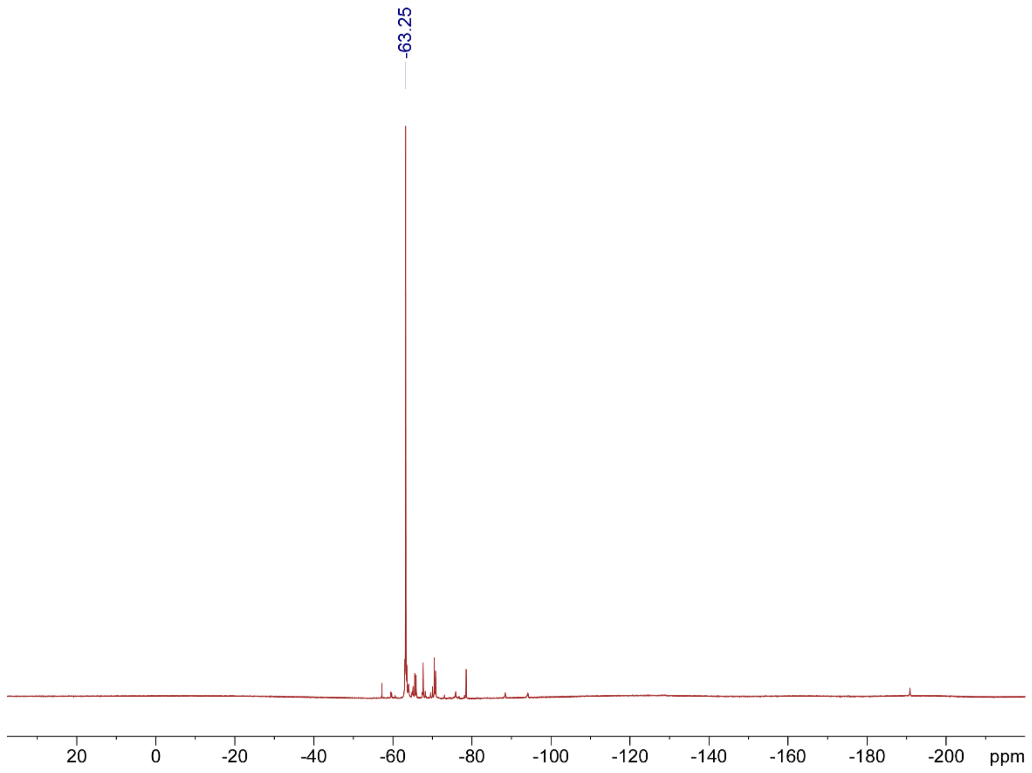

Figure S38. ^{19}F-NMR spectrum of the crude reaction mixture for the C–H insertion experiment, in $CDCl_3$. The major peak corresponds to the desired product for the reaction.

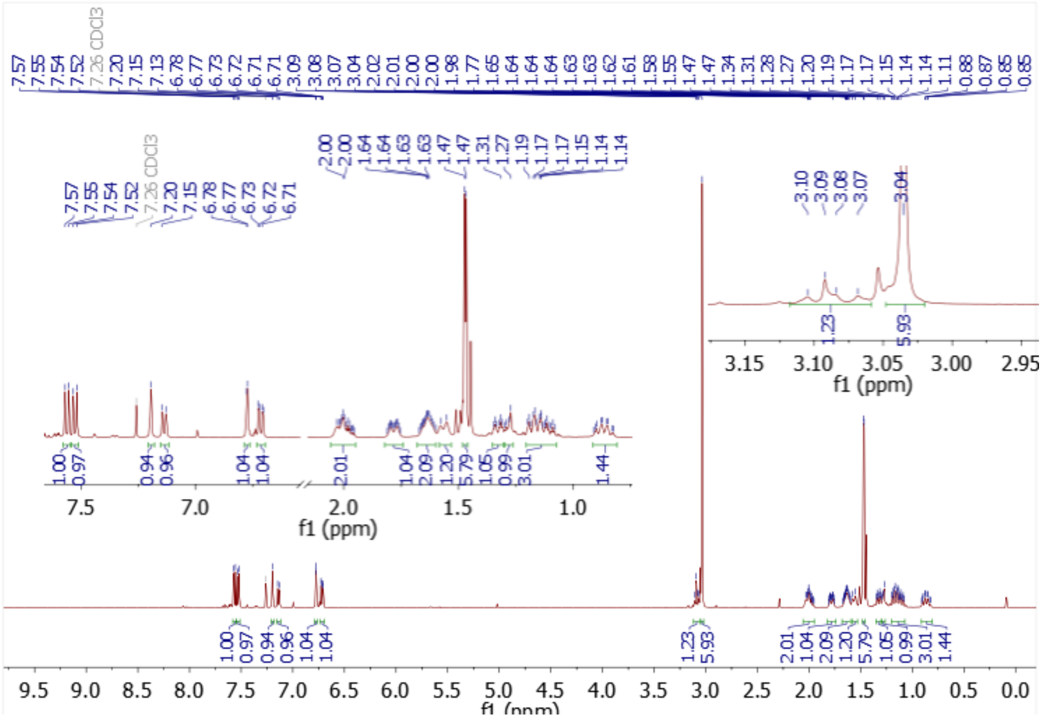

Figure S39. ^{1}H-NMR spectrum of **7** in $CDCl_3$.

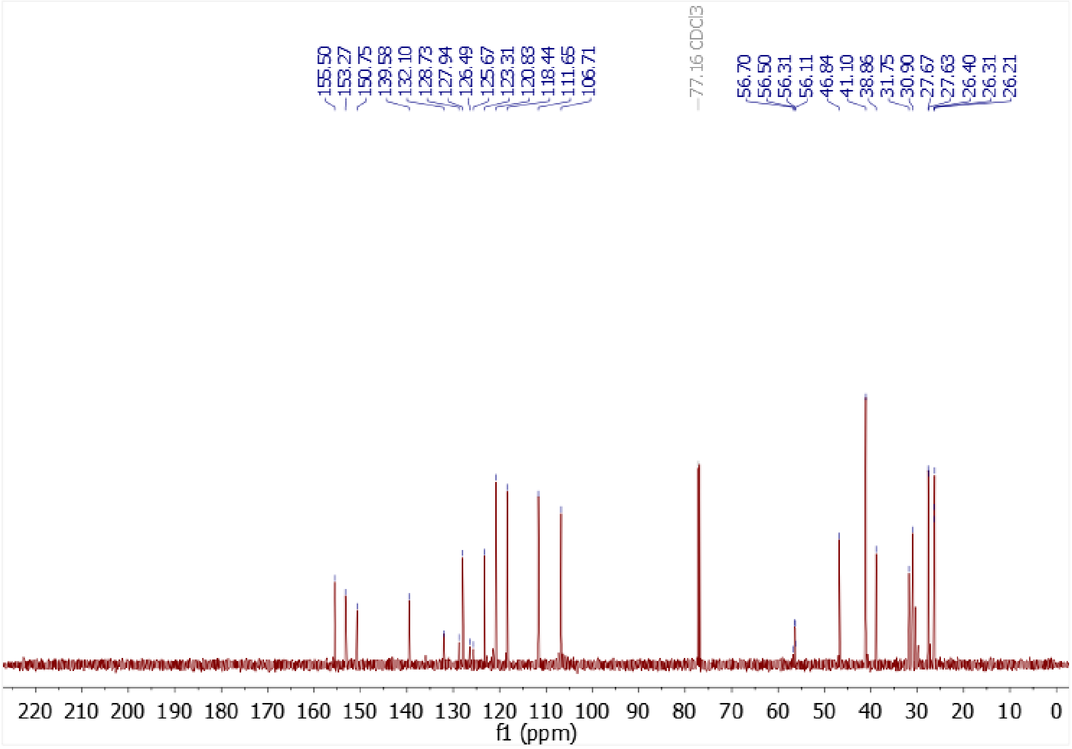

Figure S40. ^{13}C-NMR spectrum of **7** in $CDCl_3$.

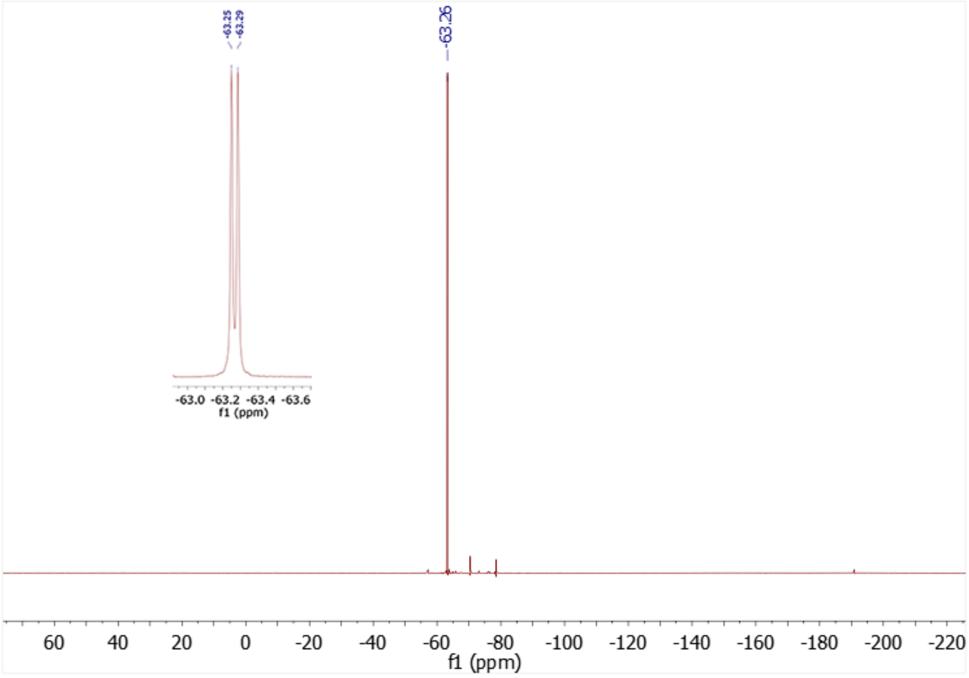

Figure S41. ^{19}F-NMR spectrum of **7** in $CDCl_3$. Inset plot shows the ^{1}H coupled spectrum.

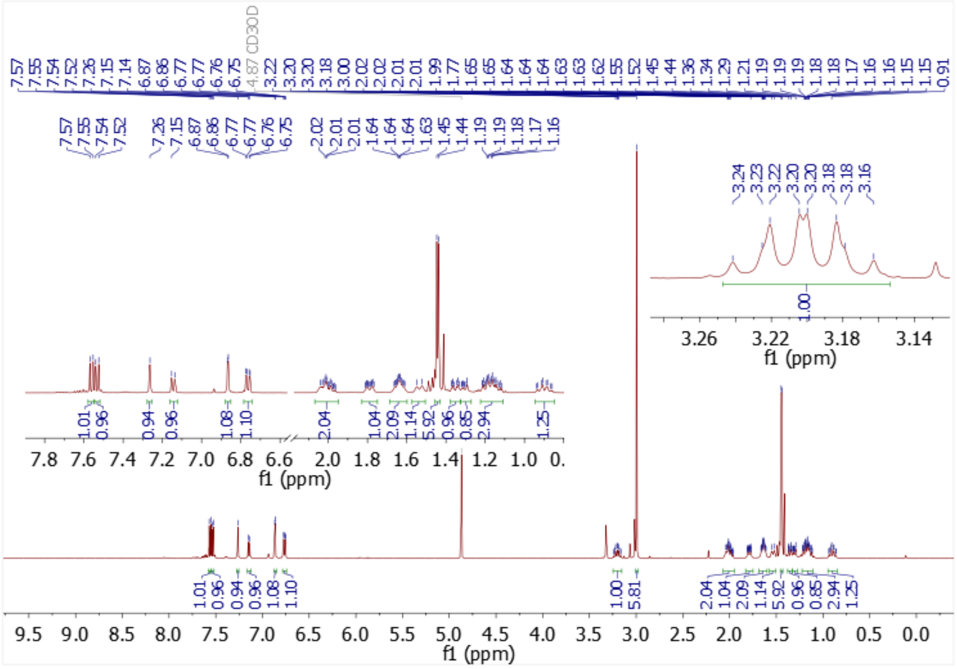

Figure S42. ^{1}H-NMR spectrum of **7** in CD_3OD.

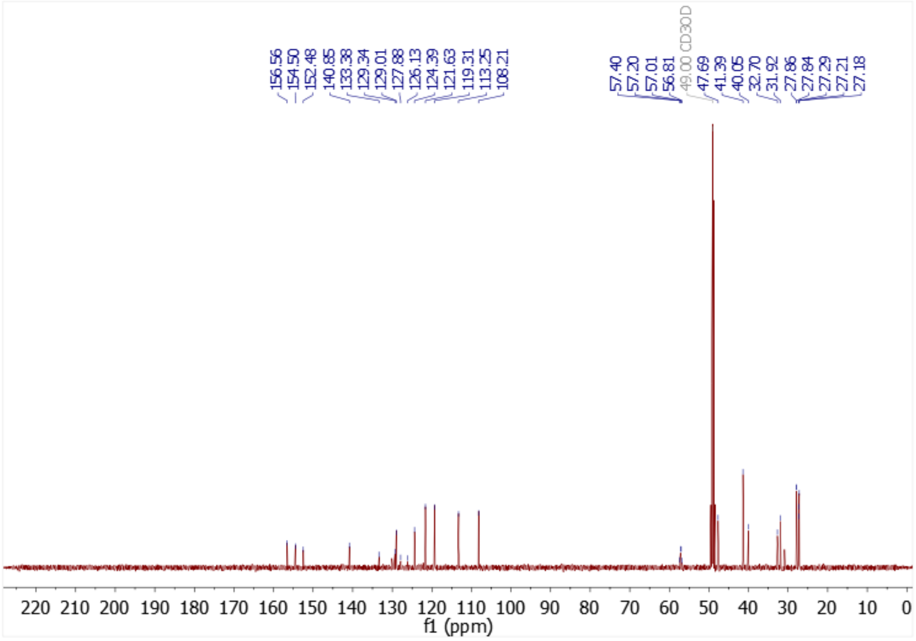

Figure S43. ^{13}C-NMR spectrum of **7** in CD_3OD.

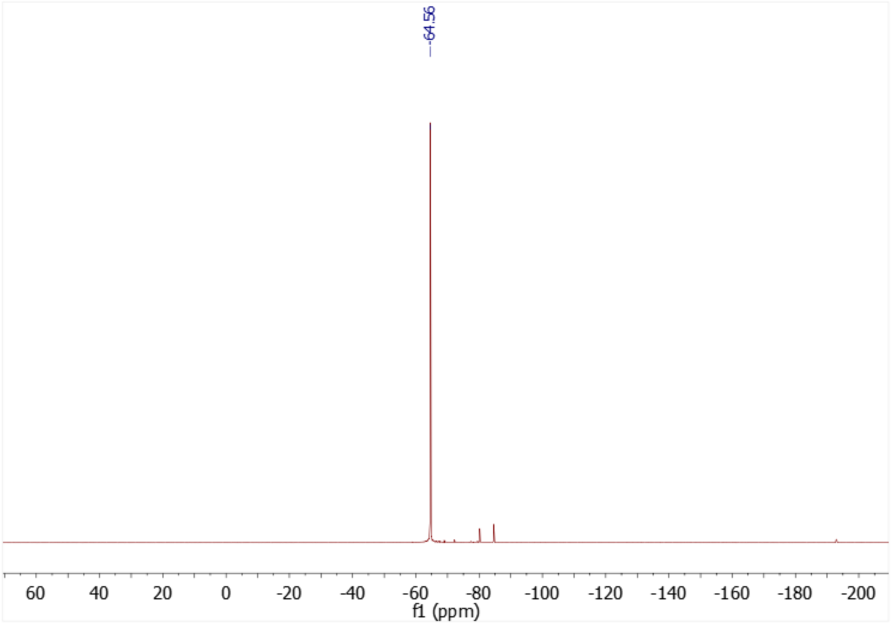

Figure S44. ^{19}F-NMR spectrum of **7** in CD_3OD.

Thermal insertion in cyclohexane:

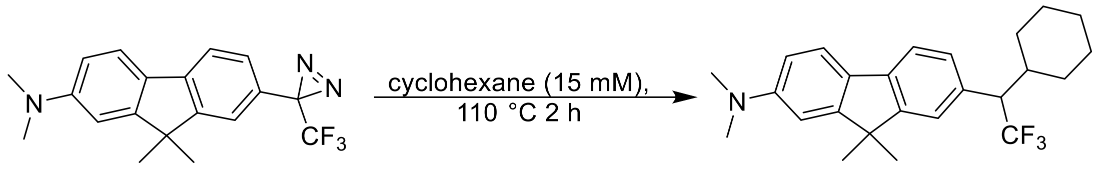

1 **7**

A solution of **1** (18.7 mg, 0,05 mmol) in cyclohexane (15 mM) was prepared under stirring in a pressure tube that was subsequently sealed with a septum and degassed 10 min with argon. The tube was put in a pre-heated oil bath and the solution was stirred for 2 h at 110 °C. The product obtained was concentrated under vacuum and purified by flash chromatography (80% pentane, 20% Et_2O), affording **7** (9.1 mg, 42% yield) as an orange solid.

Two-photon activation experiments:

General protocol for two-photon activation experiment:

In an amber glass vial, a solution of diazirine in cyclohexane (50 mM) was prepared under stirring, and a small aliquot of solution was taken through a glass capillary. One end of the capillary was taped to a microscope slide, which was placed on the instrument holder. The laser source was focused on the center of the solution portion in the capillary using the microscope lens, and it was turned on. A Zeiss confocal microscope LMS-880 equipped with a Ti:Saphire laser source (Chameleon from Coherent) was used for all two-photon activation experiments. The instrument settings for the two-photon microscope experiments were as follows: excitation wavelength 815 nm, laser power on the sample 140 mW, pulse duration 100 fs, repetition rate 80 MHz, 10x objective, focal parameter 2 μm, confocal parameter 6 μm, irradiation area 855 x 855 μm. The irradiation time for each spatial point was 2.4 μs and the distance between points in a transverse plane was 1.7 μm and 10 μm in the z-plane. Every spatial point in the sample volume was rastered 4 times. It took 6 hours to cover the entire sample volume. The temperature was monitored with an infrared temperature reader three times over the span of the experiment (during which the measured temperature remained between 20°C to 22°C). After turning the laser off, the solution in the capillary was diluted in $CDCl_3$, and both ^{19}F-^{1}H decoupled and ^{19}F-^{1}H coupled NMR spectra were acquired.

General protocol for control experiment:

In an amber glass vial, a solution of diazirine in cyclohexane (50 mM) was prepared under stirring and a small aliquot of solution was taken through a glass capillary. The solution in the capillary was transferred to an amber vial and left under the fume hood for the same duration of the activation experiment (6 hours). At the end of this time, the solution in the amber vial was diluted with $CDCl_3$ and a ^{19}F-NMR spectrum was acquired.

***19*F-NMR Spectra acquired from microscope experiments:**

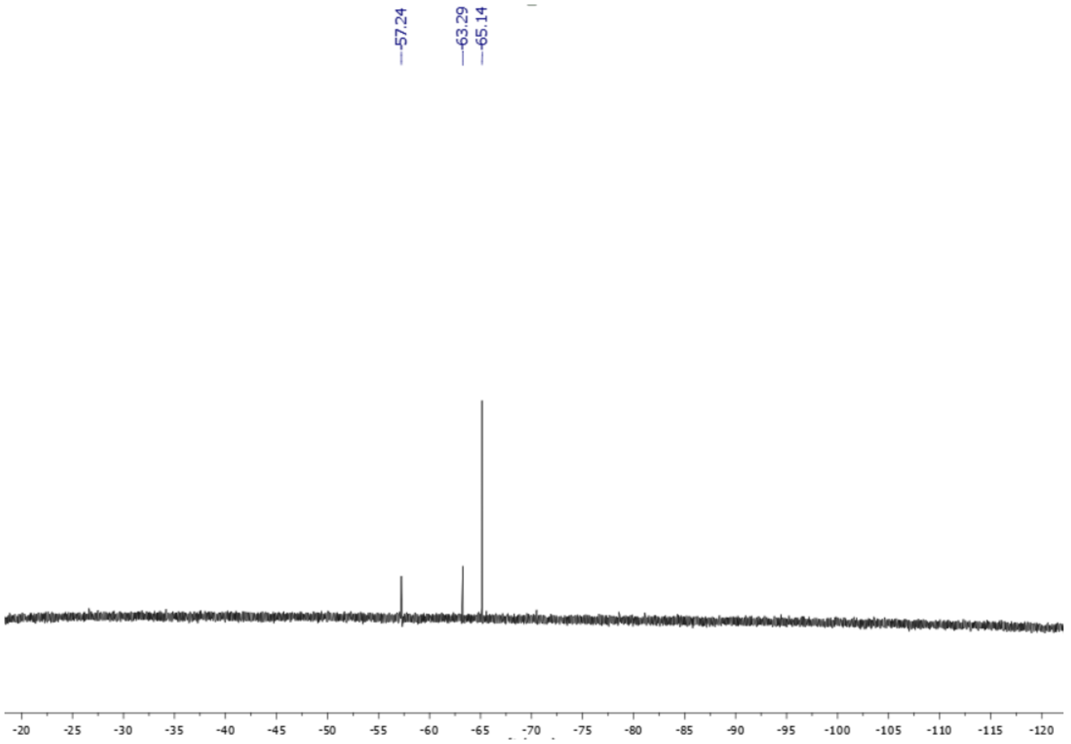

Figure S45. ^{19}F-^{1}H decoupled NMR spectrum in $CDCl_3$ following two-photon activation. The peaks shown are identified as: −57.23 ppm (diazoalkane derivative), −63.28 ppm (C–H insertion product **7**), −65.12 ppm (diazirine **1**).

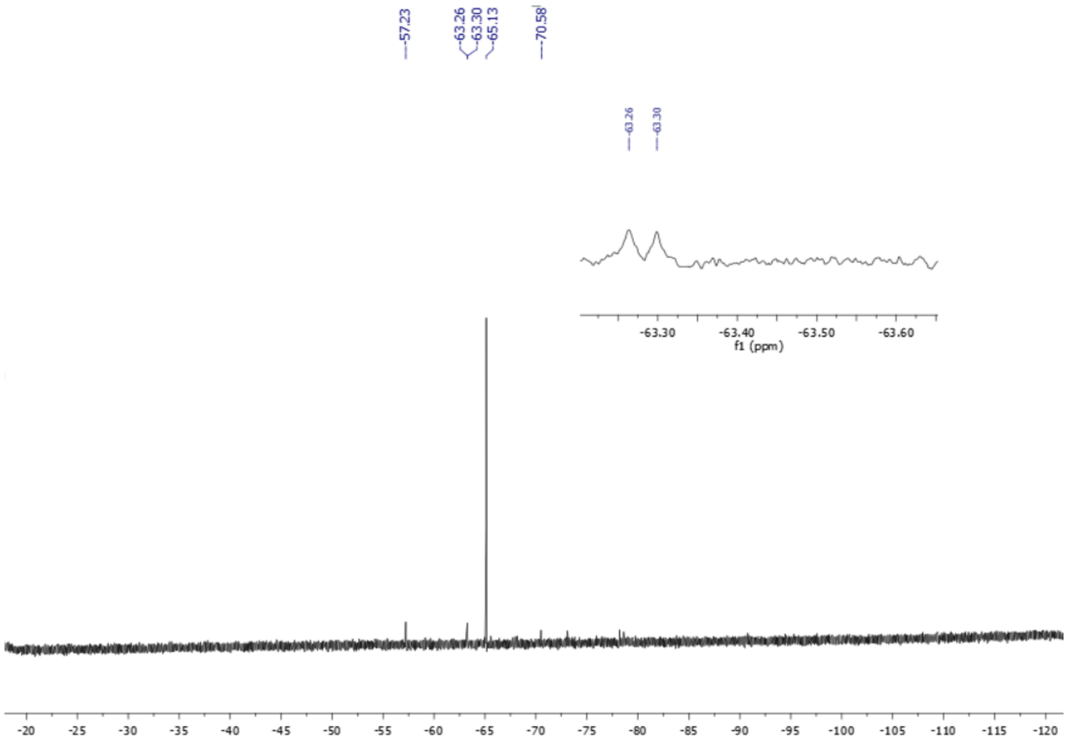

Figure S46. ^{19}F-^{1}H coupled NMR spectrum of activation experiment at the microscope in $CDCl_3$. The peaks shown are identified as: −57.23 ppm (diazoalkane derivative), −63.27 ppm (doublet, C–H insertion product **7**), −65.12 ppm (diazirine **1**).

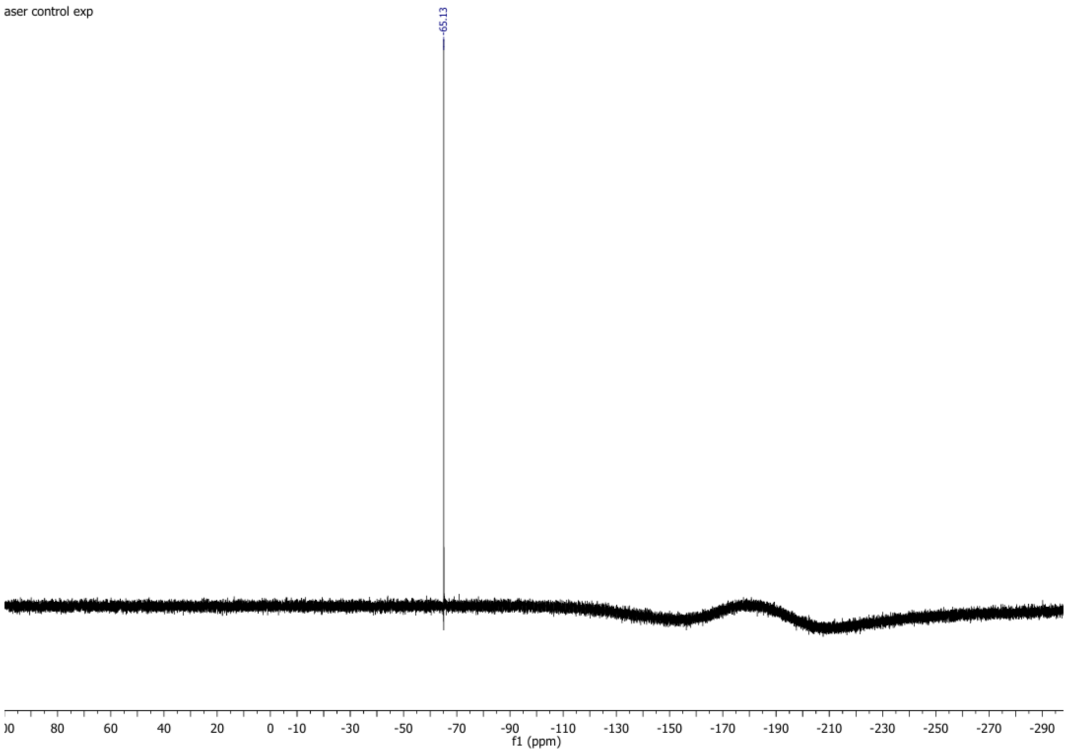

Figure S47. ^{19}F-^{1}H decoupled NMR spectrum of control experiment for microscope activation in $CDCl_3$. The peaks shown are identified as: −65.13 ppm (diazirine **1**).

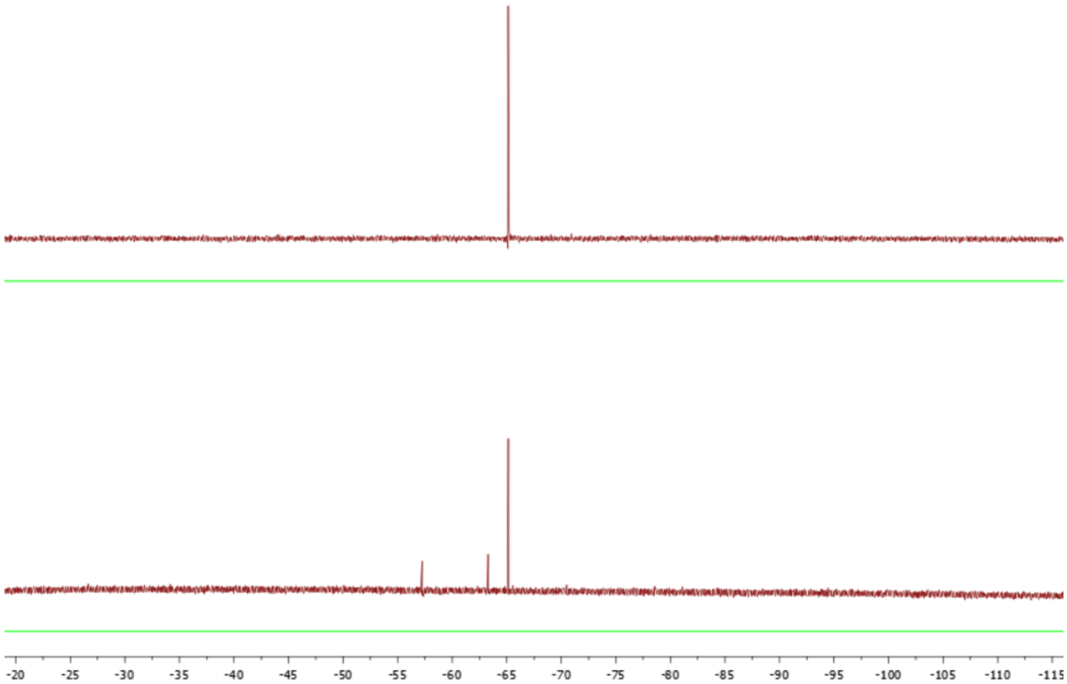

Figure S48. Comparison between ^{19}F-^{1}H decoupled NMR spectrum following near-IR light activation experiment (bottom) and control experiment spectrum (top) in $CDCl_3$.

Two-photon control experiment using bis-diazirine S.12

As a control experiment to rule out thermal activation, the two-photon activation of diazirine **S.12** was investigated, according to the two-photon excitation protocol described above. As confirmed by the ^{19}F-NMR spectrum collected at the end of the experiment (see Figure S49), *bis*-diazirine **S.12** was recovered following 195 minute of irradiation and did not get activated under the experimental conditions used. During this same 195 minute period of irradiation, fluorene–diazirine **1** exhibited substantial diazirine activation and clear C–H insertion (see Figure S50). When a longer irradiation was employed (6 hours), traces of linear diazoalkane and C–H insertion product were observed, (see Figure S51) but the percent conversion remained much lower than for fluorene–diazirine **1** (see Figure S45).

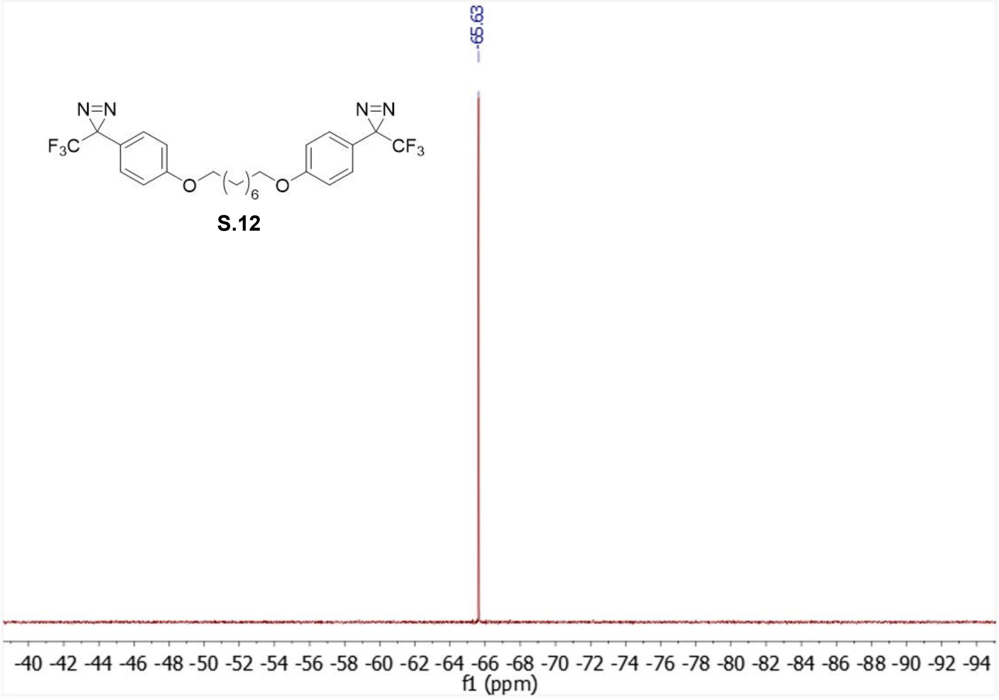

Figure S49. ^{19}F-^{1}H decoupled NMR spectrum of the two-photon activation experiment run on control diazirine **S.12**, following 195 minutes of activation. The single peak at −65.63 ppm is assigned to **S.12** (in perfect accordance with the literature[8]), demonstrating a complete lack of activation of the diazirine under the experimental conditions tested.

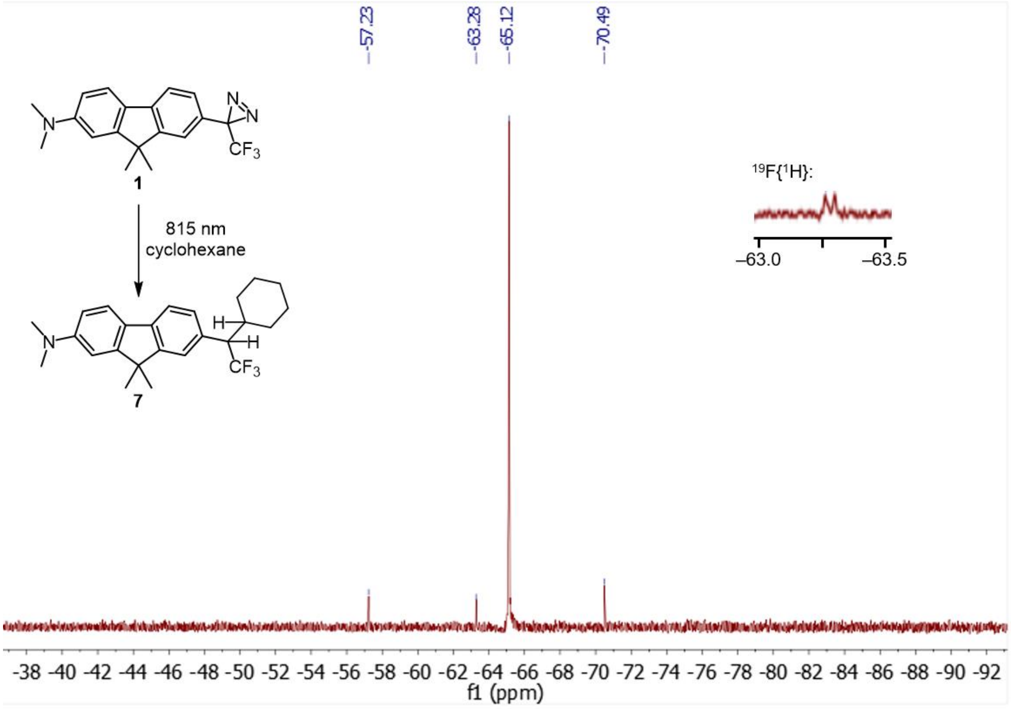

Figure S50. ^{19}F-^{1}H decoupled NMR spectrum (with inset ^{19}F-^{1}H coupled spectrum) of the two-photon activation experiment run on fluorene–diazirine **1**, following 195 minutes of activation. Signals at –57.23 ppm (for the linear diazoalkane) and –63.28 ppm (for the C–H insertion product) clearly indicate two-photon activation for diazirine **1** (–66.12 ppm) under these reaction conditions.

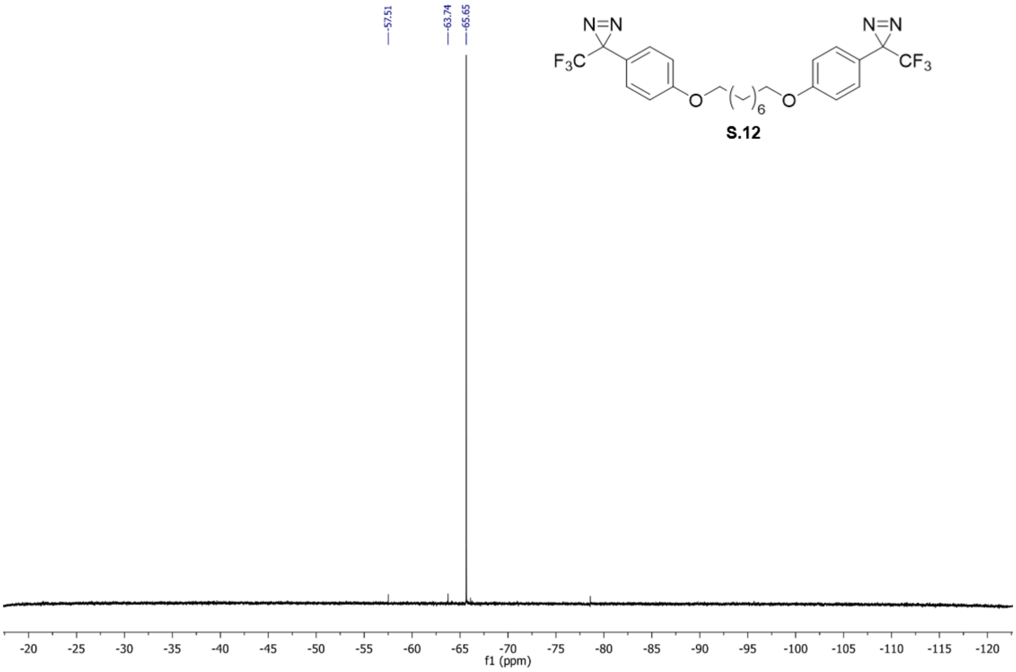

Figure S51. ^{19}F-^{1}H decoupled NMR spectrum of the two-photon activation experiment run on control diazirine **S.12**, following 6 hours of activation. The appearance of new signals at –57.51 ppm (for the linear diazoalkane) and –63.74 ppm (for the C–H insertion product) indicates that **S.12** can also be activated using two-photon excitation, but that the process is much less efficient than that for fluorene–diazirine **1** (compare with Figure S45).

Photophysical Characterization of Diazirine 1:

Determination of one-photon photochemical parameters:

The compound of interest (diazirine **1**) was dissolved in the desired reaction substrate (cyclohexane, *n*-hexane, or dodecane), to afford a stock solution of 10 mM, which was maintained in the dark at 4°C. Aliquots were diluted (in the same reaction substrate) to a final concentration between 20 and 50 μM to perform the 1-photon irradiation experiments, at two different wavelengths: 405 and 446 nm. The irradiation was performed into an optical bench by means of laser diodes focused to infinity with a diameter of 2–3 mm. A variable neutral density filter (Edmund Optics, #63-048) was used to reduce the power to a desired value. The beam intensity was measured with a Coherent Fieldmaster and a LM2-Vis sensor. The cuvette was stirred continuously during the photolysis to avoid inhomogeneous depletion of the reactant, and the spectra were taken with an Ocean Optics Chem2000 diode array spectrometer. A typical run is depicted in Figure S52. The presence of isosbestic points indicates that the reaction exhibits clean conversion, with just two absorbent species.

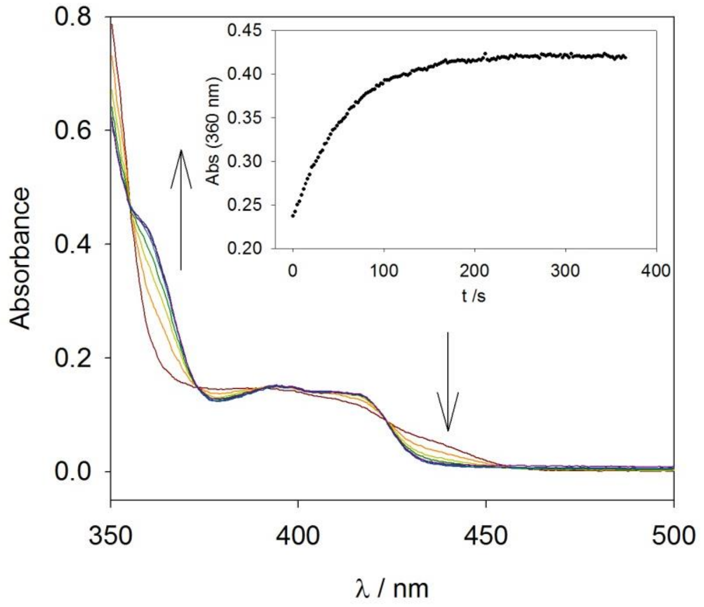

Figure S52. Typical run of an irradiation experiment. Solvent: cyclohexane. P = 1.79 mW. λ = 405 nm. c = 29 μM. Inset: Absorbance trace at 360 nm.

The analysis was performed using the complete spectra,[9] integrating and fitting the following differential equation to the experimental data:

$$\frac{dn_P}{dt} = I_{beam} \cdot \left(1 - 10^{-Abs_T}\right) \cdot \frac{Abs_R}{Abs_T} \cdot \varphi_{PC} \qquad (1)$$

where φ_{PC} is the adjustable parameter.[10] A Matlab script fits the concentration profiles to the obtained data and yields three parameters: the spectra for pure reactant and product, the concentration vs. time profile of both compounds, and the best fitted φ_{PC} value. The results for the data shown in Figure S52 are depicted in Figure S53.

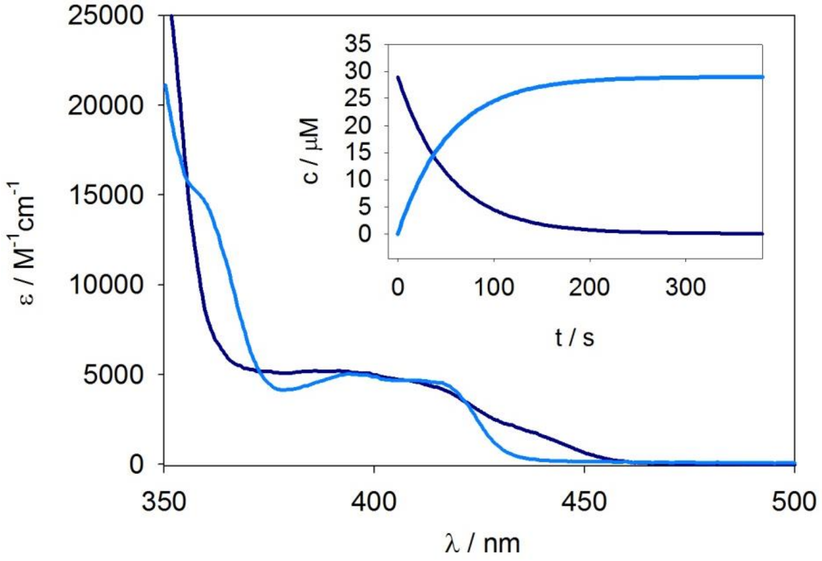

Figure S53. Analysis of data from Figure S52. Dark blue line: spectrum of diazirine **1**. Light blue line: Photoproduct spectrum. Inset: Concentrations of reactant and product during irradiation. φ_{PC} =0.733.

Table S4: Results of irradiation experiments at different conditions[(a)]

entry	solvent	wavelength (nm)	P (mW)	φ_{PC}
1	cyclohexane	405	1.79	0.733
2	cyclohexane	405	0.87	0.748
3	cyclohexane	405	0.89	0.720
4	cyclohexane	446	3.94	0.879
5	cyclohexane	446	3.94	0.885
6	*n*-hexane	405	0.89	0.778
7	*n*-hexane	405	0.89	0.773
8	*n*-hexane	446	3.97	0.836
9	dodecane	405	0.87	0.603

(a) T = 25 °C

One-photon fluorescence measurements:

Diazirine **1** shows rather strong fluorescence. Figure S54 shows the increase of emission during irradiation at 405 nm. This change in emission offers an adequate means to follow the photoconversion during irradiation in a 2-photon microscope.

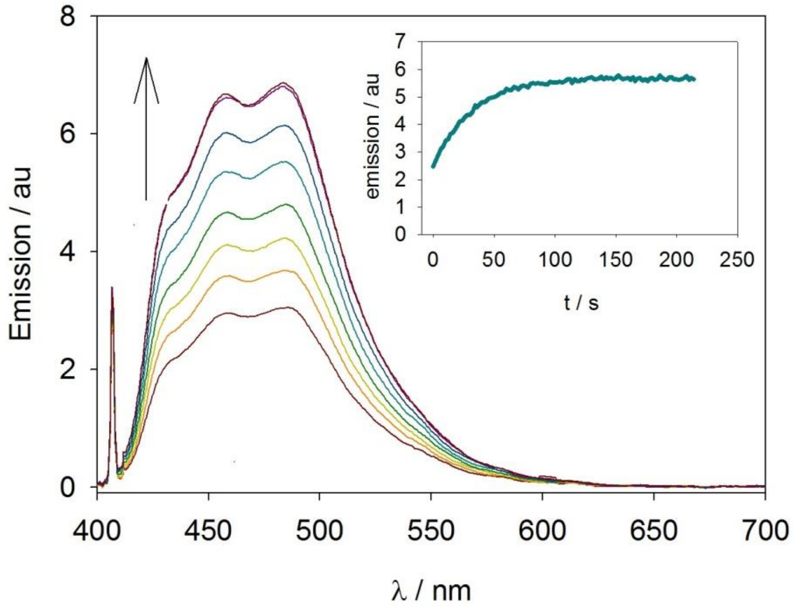

Figure S54. Fluorescence of the fluorine–diazirine conjugate (**1**) and subsequent increase during irradiation with 405 nm. Inset: emission trace at 498 nm. Solvent = cyclohexane. Spectra are not corrected.

Determination of two-photon absorption cross-section:

The determinations for absorption cross section were performed in a microscope Zeiss LSM980 with a Ti-Sapphire laser excitation (Spectraphysics Mai Tai eHP DS, 80 MHz, 70 fs pulse at 800 nm). The modified Webb equation[10] was fitted to the photolysis data. The complete procedure is published elsewhere.[11]

$$n_{5HT} = \frac{1}{2} g \varphi_{fot} C \delta \frac{8n\langle P\rangle^2}{\pi\lambda} \Delta t \qquad (2)$$

The samples were dissolved in n-dodecane to prevent undesired evaporation. The quantum yield of photolysis was considered as unchanged among 1P and 2P excitation at the same conditions. The irradiation was done in a nanowell cuvette array (Fluidic 18, Chipshop, cat #10000201, wells in structure 1, well volume = 2.38×10^{-7} cm^3). Images comprising 80% of the total area of the well were scanned, in order to avoid depletion in single spots. The images were processed with ImageJ/FIJI.

Figure S55 shows a typical response during irradiation at 800 nm through a 20X NA=0.9 objective. The increase in fluorescence indicates the extent of the photolysis. A biexponential fitting is used to take into account the diffusion of new reactant into the well during photolysis.[11]

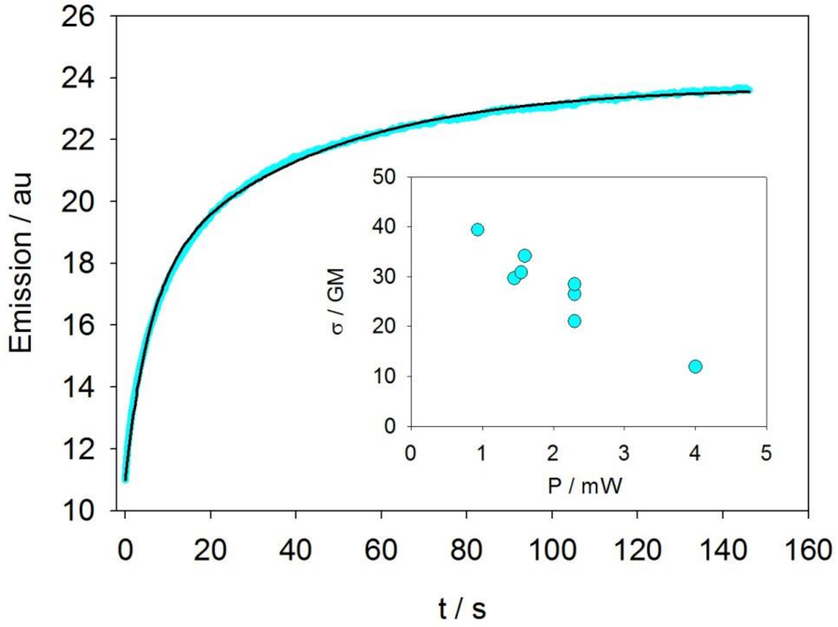

Figure S55. Average emission during irradiation at 800 nm in two-photon regime. (P_{avg} = 2.3 mW, λ = 800 nm). Cyan: experimental values. Black line: best fit at σ = 21.0 GM. Inset: values of fitted 2P cross section in GM for different power conditions.

Table S5 shows a summary of several measurements in the 2P regime. The absorption cross section ranges around 40 GM at 1 mW average power, becoming lower at higher excitation power due to the partial depletion of the reactants in the diffraction-limited focus spot.

Table S5: Results of two-photon cross-section determinations

entry	solvent	P (mW)	σ (GM)
1	dodecane	0.94	39.3
2	dodecane	1.45	29.6
3	dodecane	1.55	30.8
4	dodecane	1.60	34.1
5	dodecane	2.30	26.4
6	dodecane	2.30	21.0
7	dodecane	2.30	28.4
8	dodecane	4.00	11.9

Differential Scanning Calorimetry (DSC):

General protocol for DSC analysis:

A sample of the substance to be analyzed (4–5 mg) was placed in a Tzero aluminum hermetic pan and sealed by a matching lid. The pan was pierced with a small pinhole to allow evolution of nitrogen gas. The pan was placed in the oven of DSC25 device (TA instruments) and heated from 0 °C to 250 °C at the rate of 10 °C/min, with an identical empty pan as reference. The oven was constantly flushed with a 50 mL/min flow of nitrogen. The device recorded the difference in heat flow between the reference and the studied sample, allowing the assignment of T_{onset} and T_{max}. DSC analysis for **1** was conducted 3 times. A representative DSC trace is provided below.

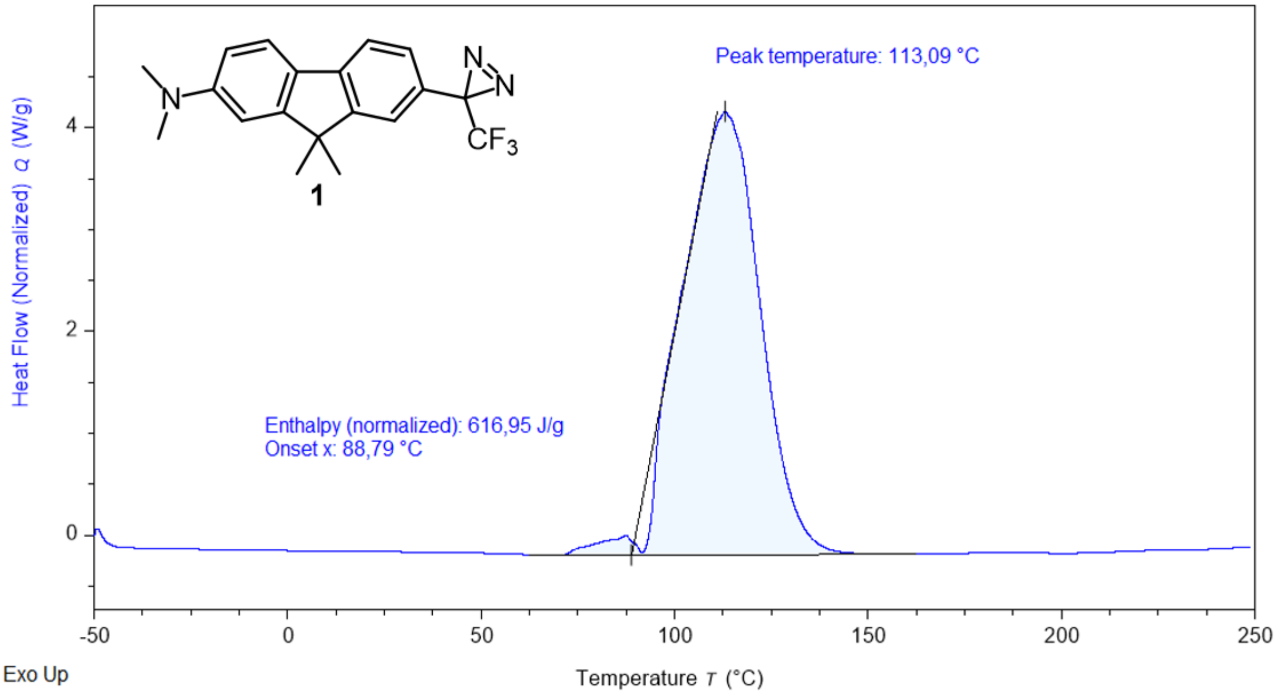

Figure S56. Representative DSC trace for **1**.

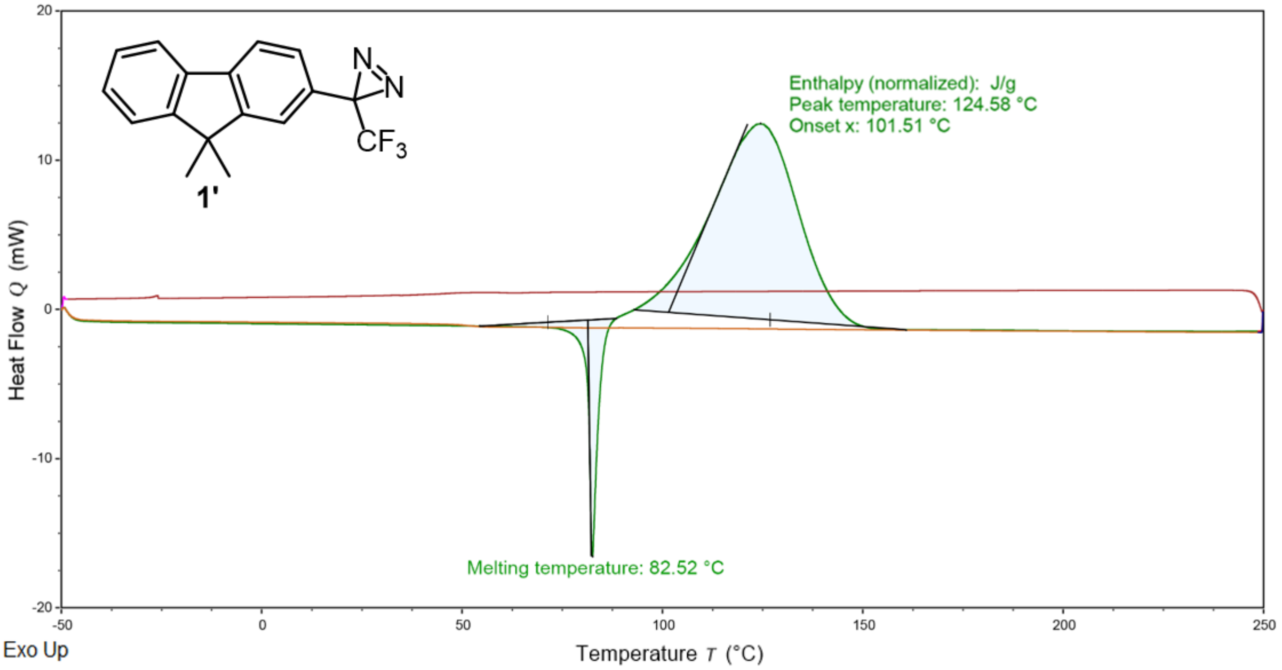

Figure S57. DSC trace for **1'**.

Supplementary Figures:

UV-Vis spectra:

All the UV-Vis spectra acquired in Spectranalyzed™ MeOH and anhydrous cyclohexane were obtained preparing a 0.1 and/or 0.05 mM solution of the sample analyzed. For any sample, the analysis was conducted three times. Representative UV-Vis spectra are provided below.

UV-Vis spectra in MeOH:

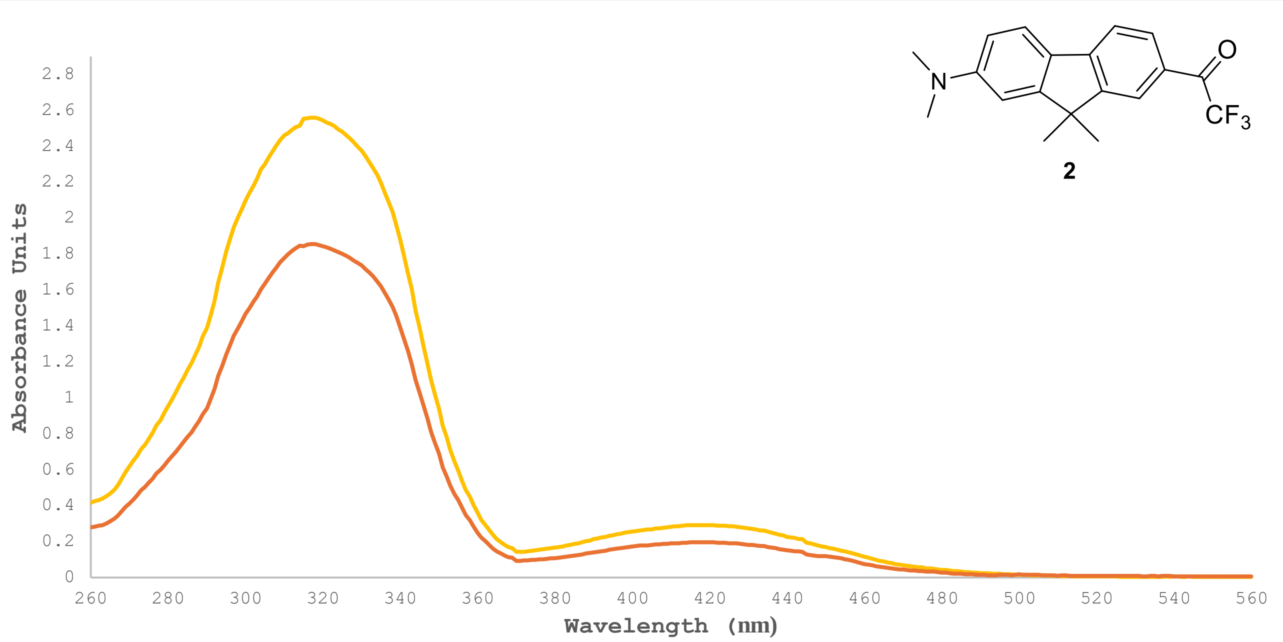

Figure S58. Absorbance UV-Vis spectrum for **2** at 0.1 and 0.05 mM in methanol.

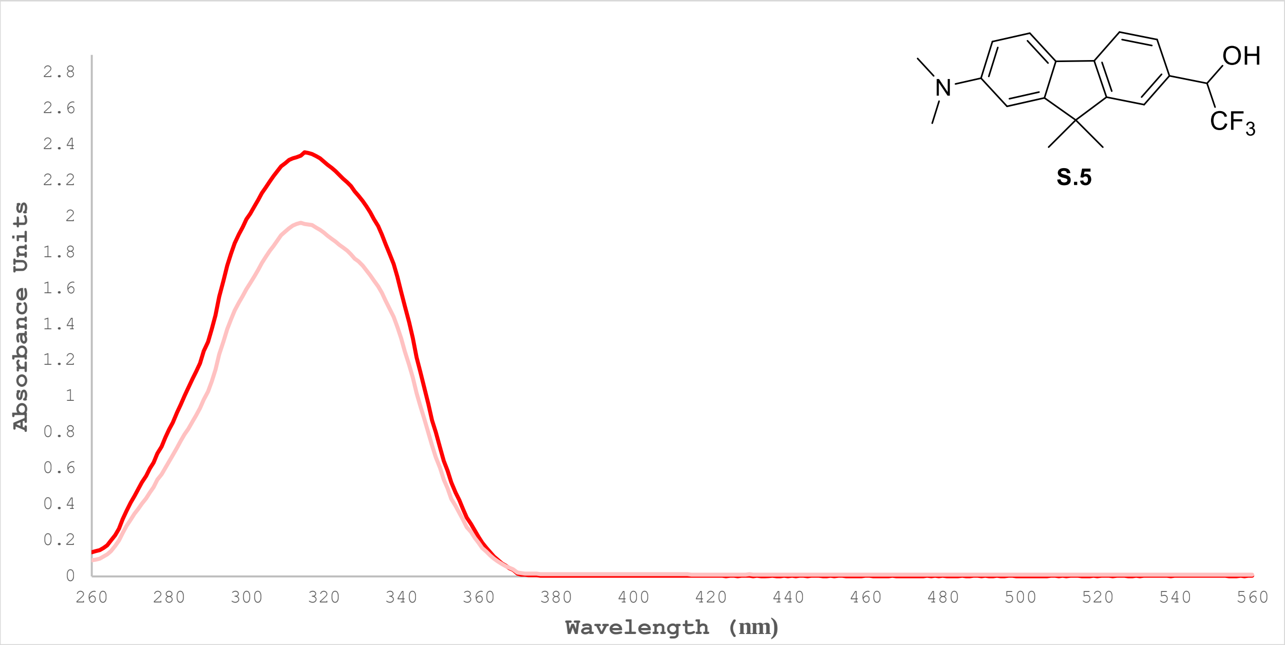

Figure S59. Absorbance UV-Vis spectrum for **S.5** at 0.1 and 0.05 mM in methanol.

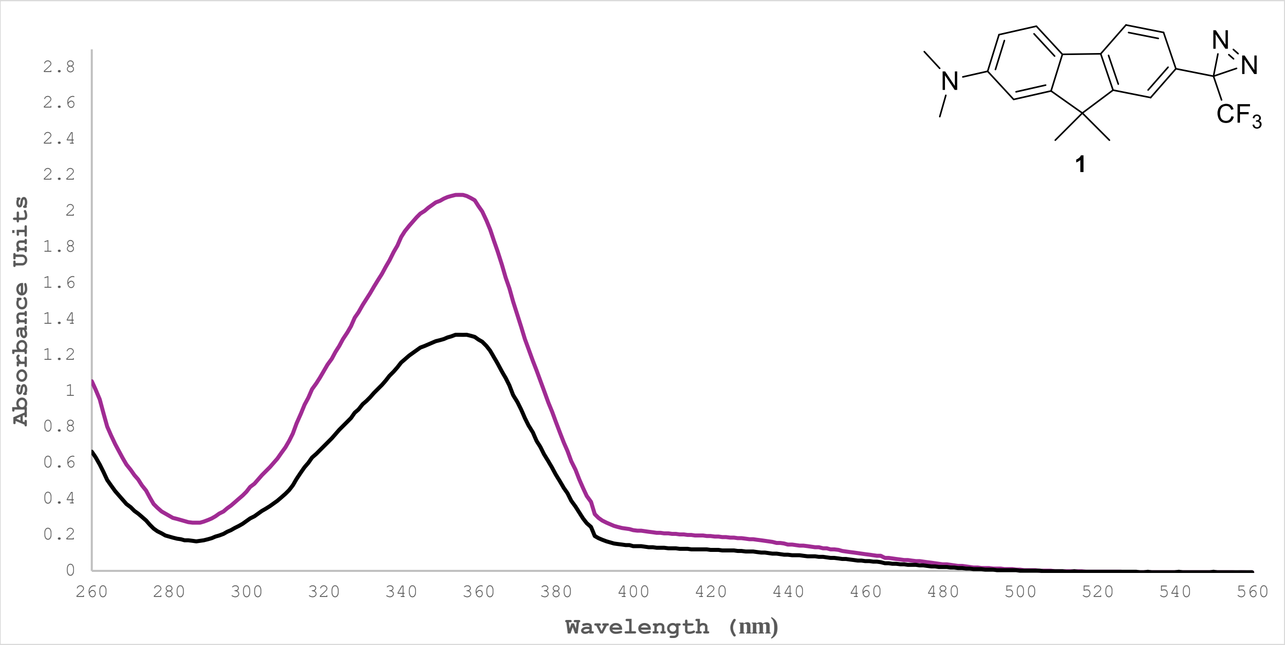

Figure S60. Absorbance UV-Vis spectrum for **1** at 0.1 and 0.05 mM in methanol.

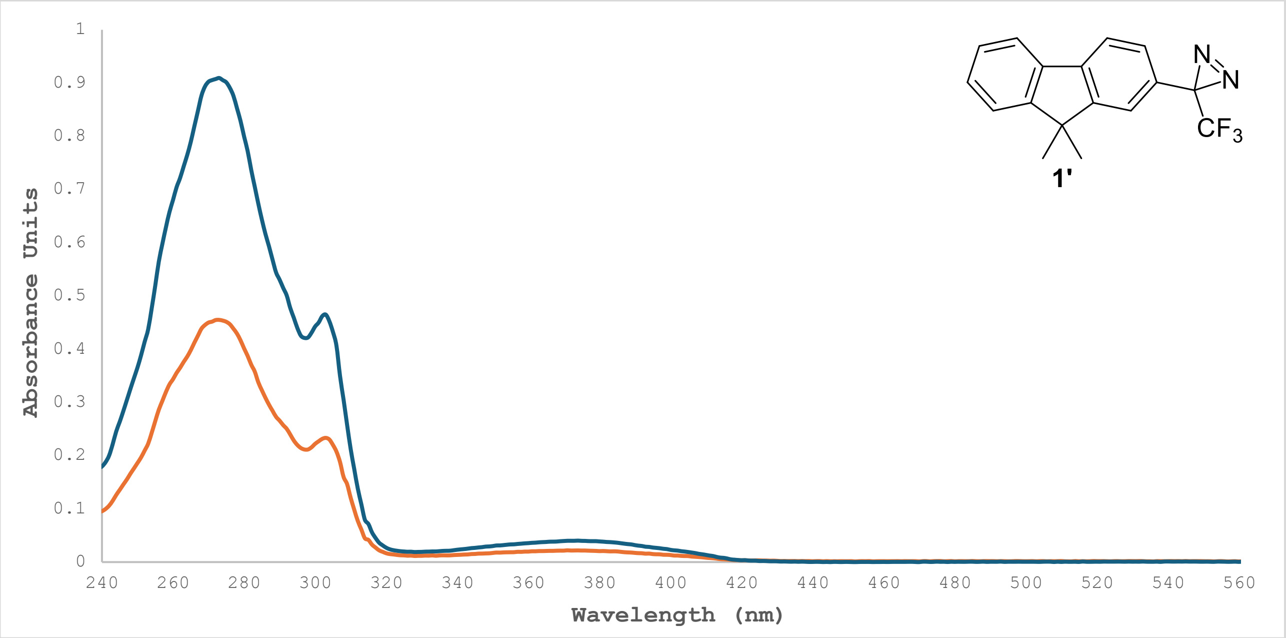

Figure S61. Absorbance UV-Vis spectrum for **1'** at 0.1 and 0.05 mM in methanol. Note the shifted x-axis relative to other plots in this section of the SI, which is necessary to accommodate the blue-shifted absorbance spectrum relative to the dimethylaminofluorenes.

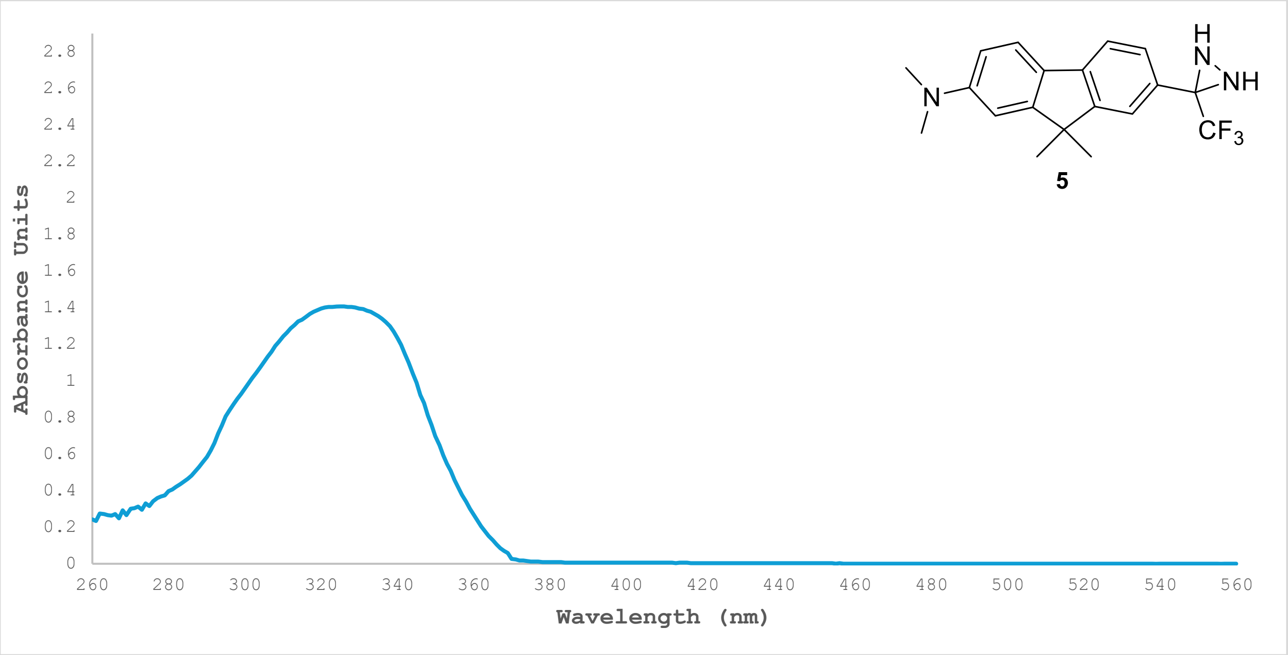

Figure S62. Absorbance UV-Vis spectrum for **5** at 0.05 mM in methanol.

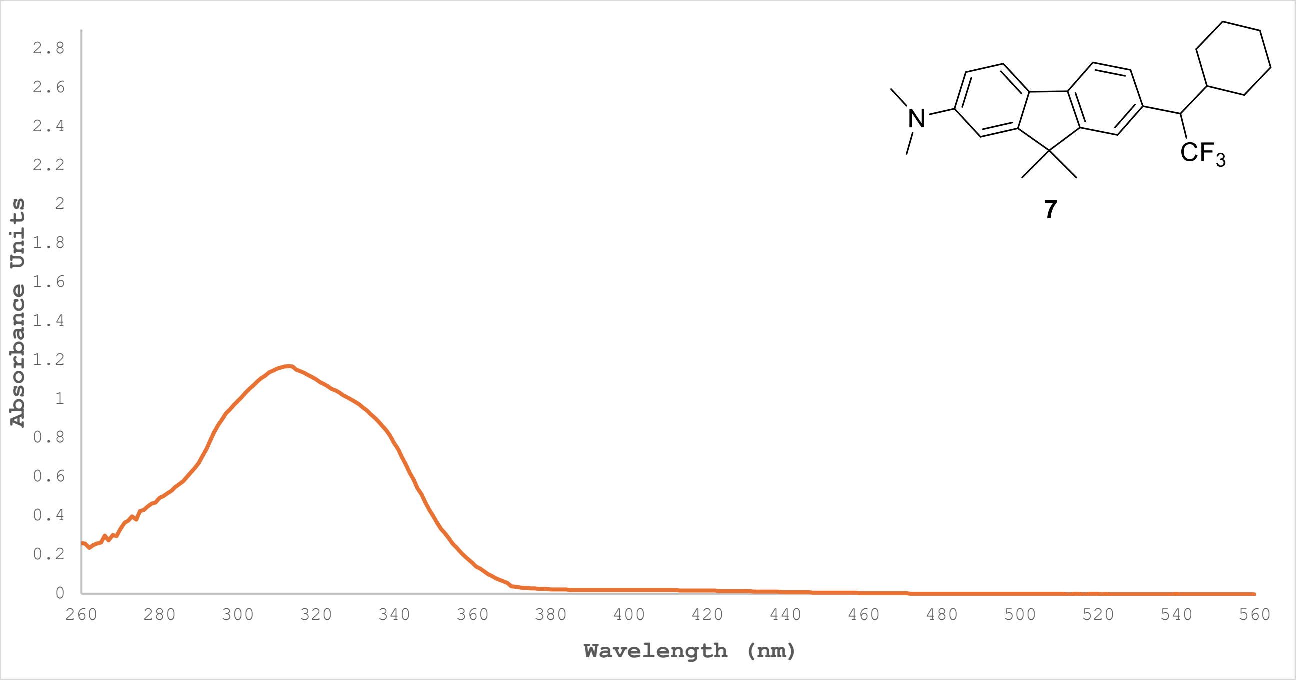

Figure S63. Absorbance UV-Vis spectrum for **7** at 0.05 mM in methanol.

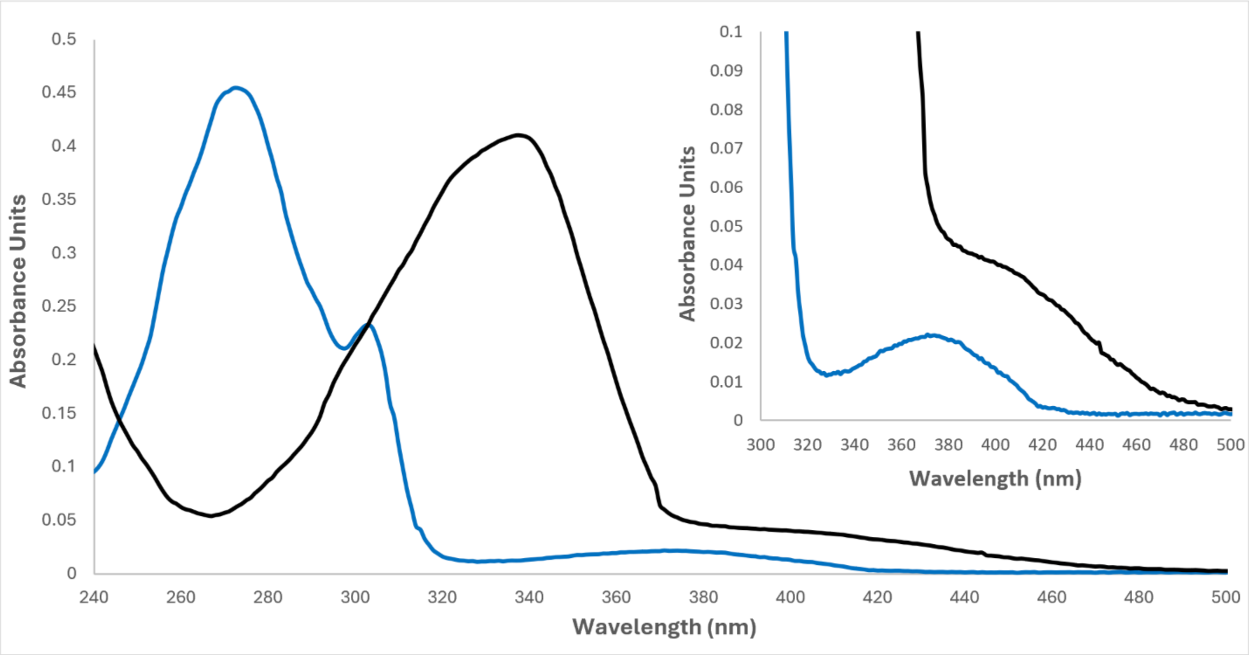

Figure S64. Comparison of absorbance UV-Vis spectrum for fluorene–diazirine **1** (black) vs. **1'** (blue) at 0.05 mM in methanol.

UV-Vis spectra in cyclohexane:

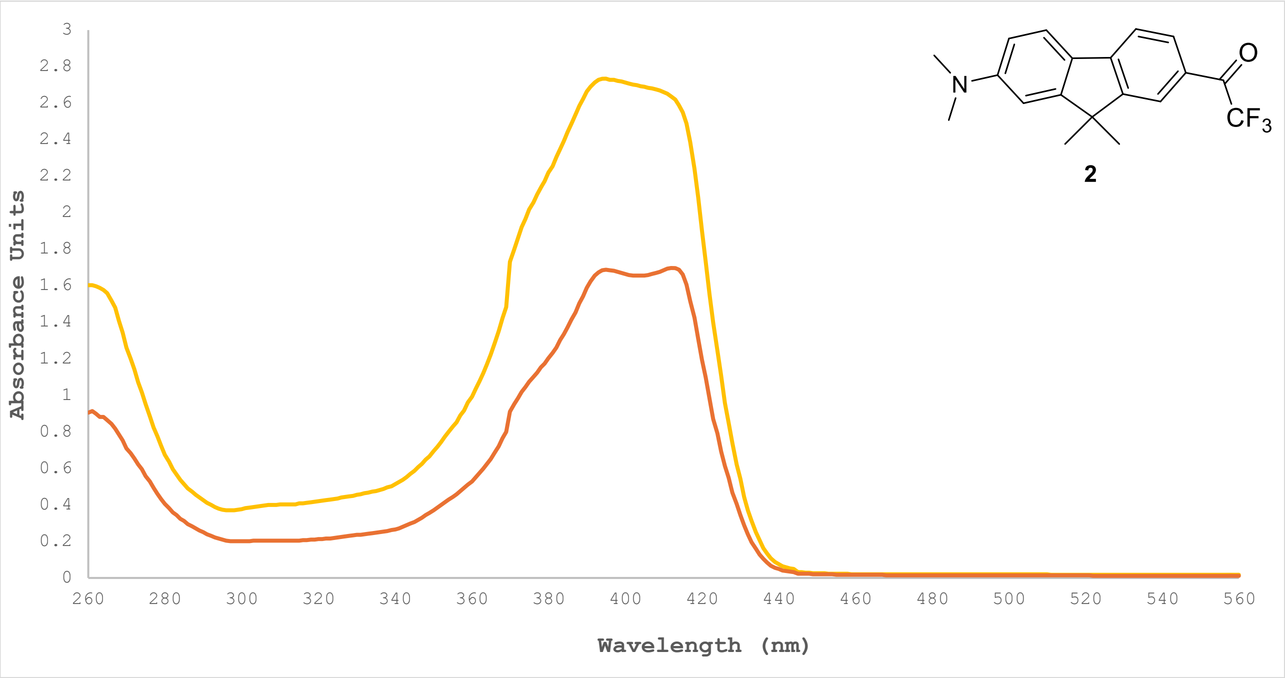

Figure S65. Absorbance UV-Vis spectrum for **2** at 0.1 and 0.05 mM in cyclohexane.

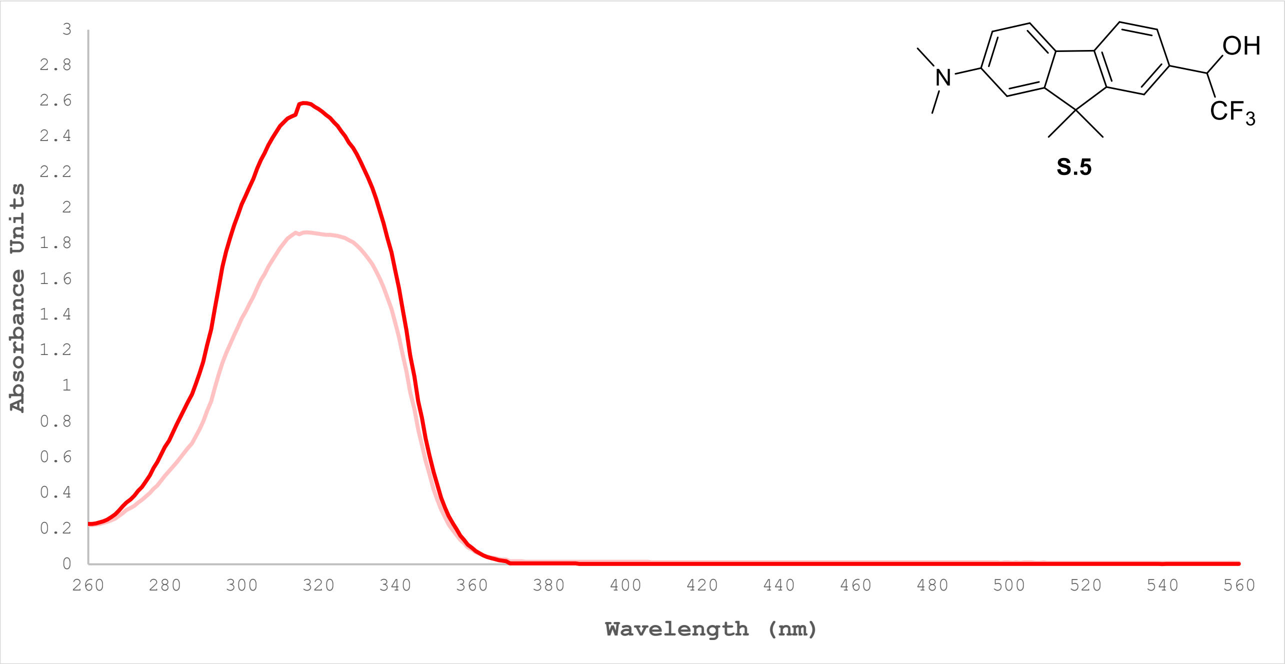

Figure S66. Absorbance UV-Vis spectrum for **S.5** at 0.1 and 0.05 mM in cyclohexane.

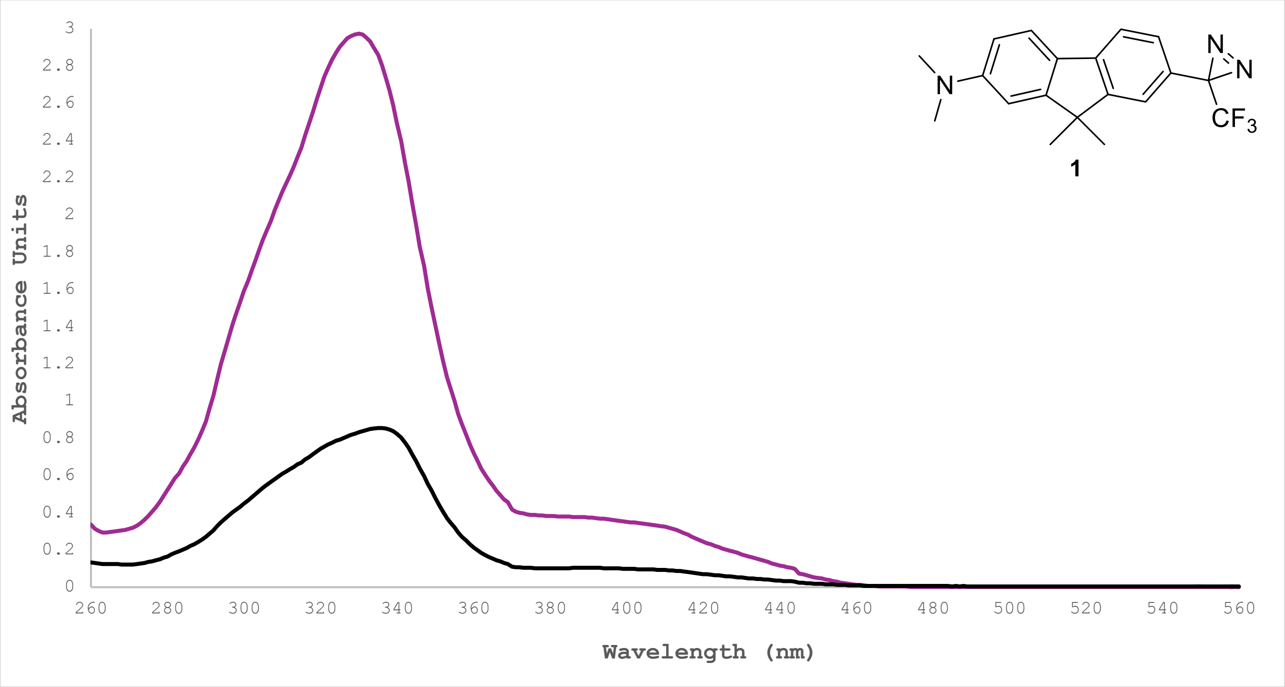

Figure S67. Absorbance UV-Vis spectrum for **1** at 0.1 and 0.05 mM in cyclohexane.

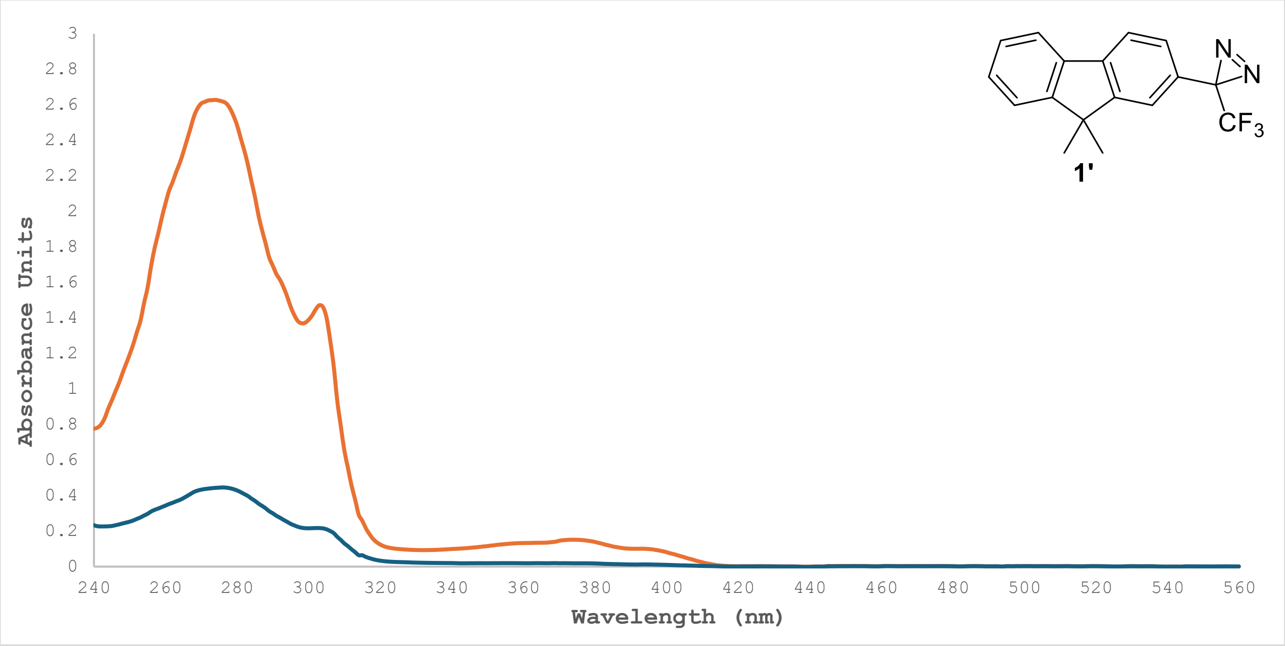

Figure S68. Absorbance UV-Vis spectrum for **1'** at 0.1 and 0.05 mM in cyclohexane. Note the shifted x-axis relative to other plots in this section of the SI, which is necessary to accommodate the blue-shifted absorbance spectrum relative to the dimethylaminofluorenes.

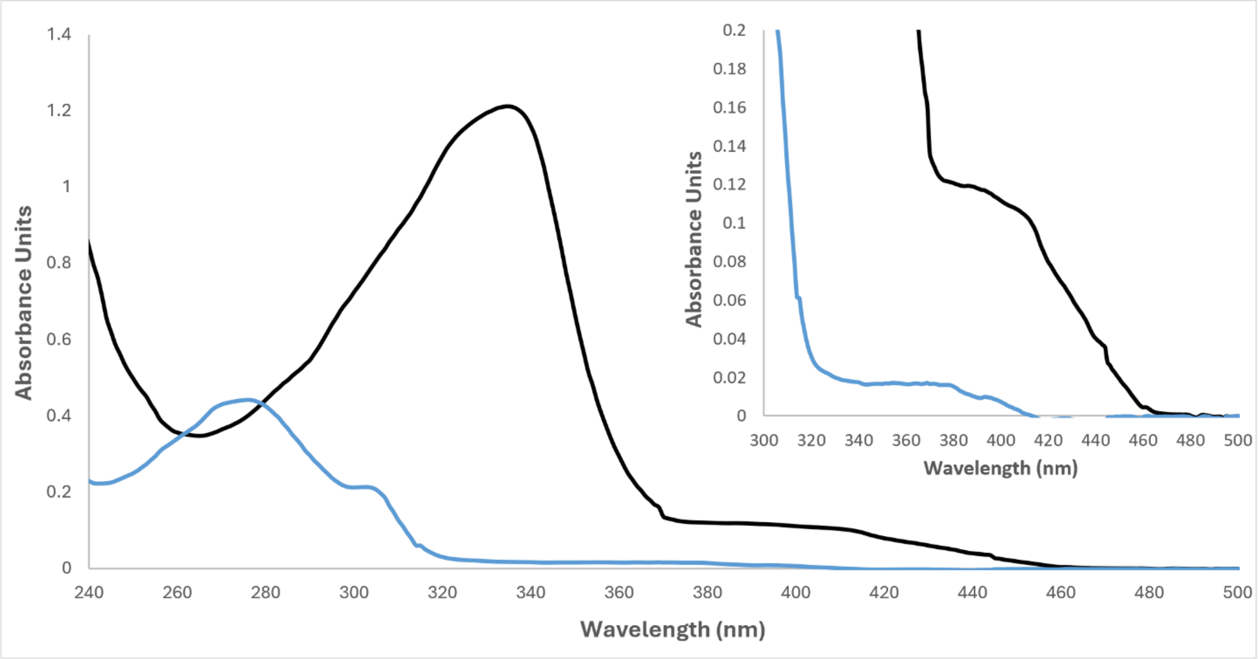

Figure S69. Comparison of absorbance UV-Vis spectrum for fluorene–diazirine **1** (black) vs. **1'** (blue) at 0.05 mM in cyclohexane.

Home-made photo chamber:

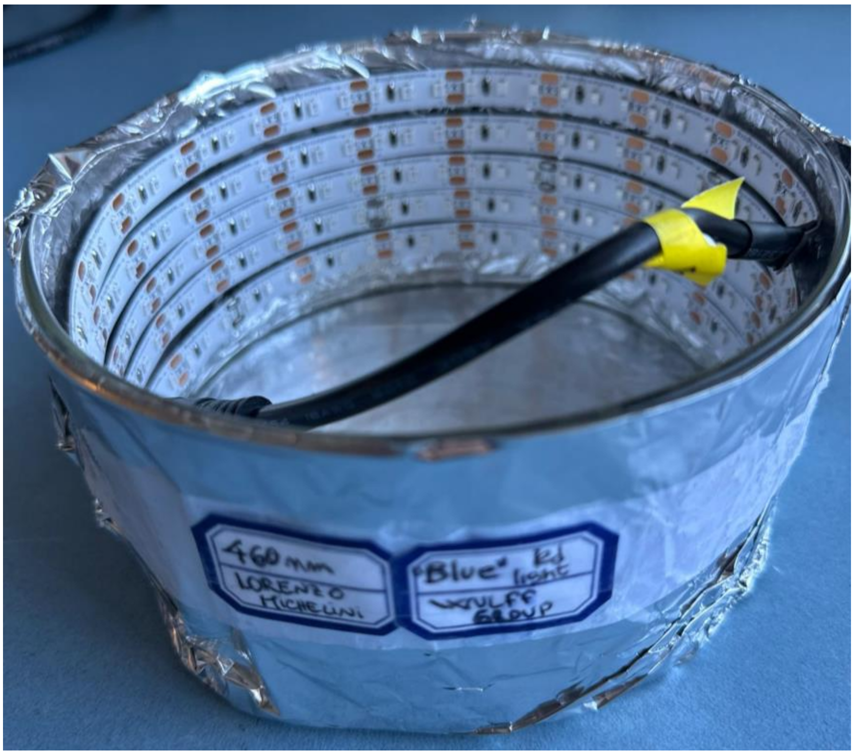

Figure S70. Photo chamber used for the photochemical experiment.

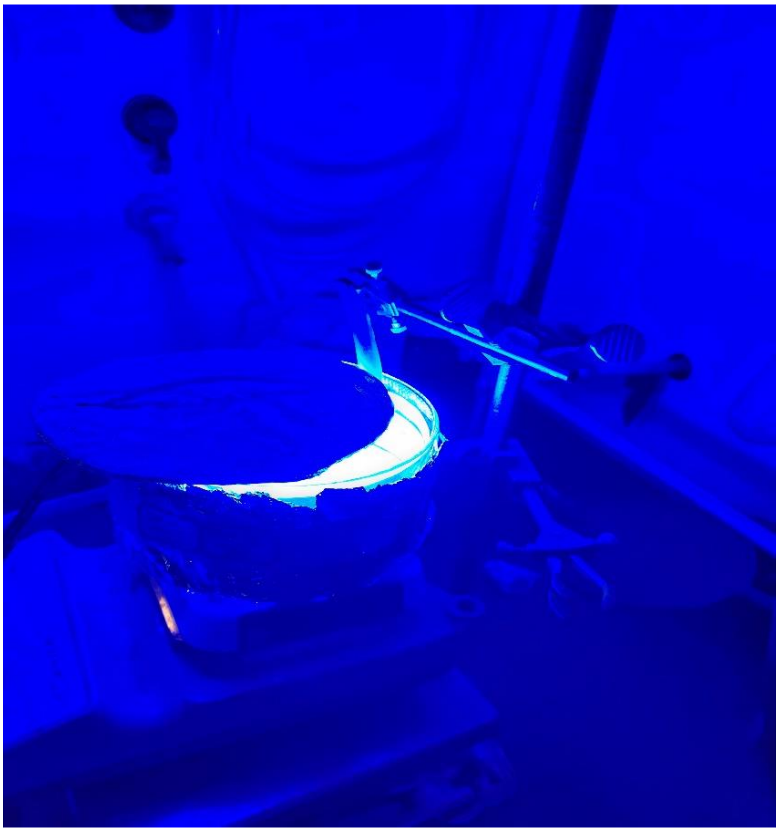

Figure S71. Photo-chamber (460 nm) with the light on.

Technical details for LED lights (Waveform Lighting catalogue number 7041.460):

SimpleColor™ Blue LED Strip Lights (460 nm, 5 m, 4.5 W/m)

88 Lumens per foot

Peak wavelength: 460 nm

Dominant wavelength: 465 nm

Spectral output FWHM: 19.5 nm

CIE (xy): (0.1391, 0.0490)

WPE: 33%

Illuminance: 44 lux

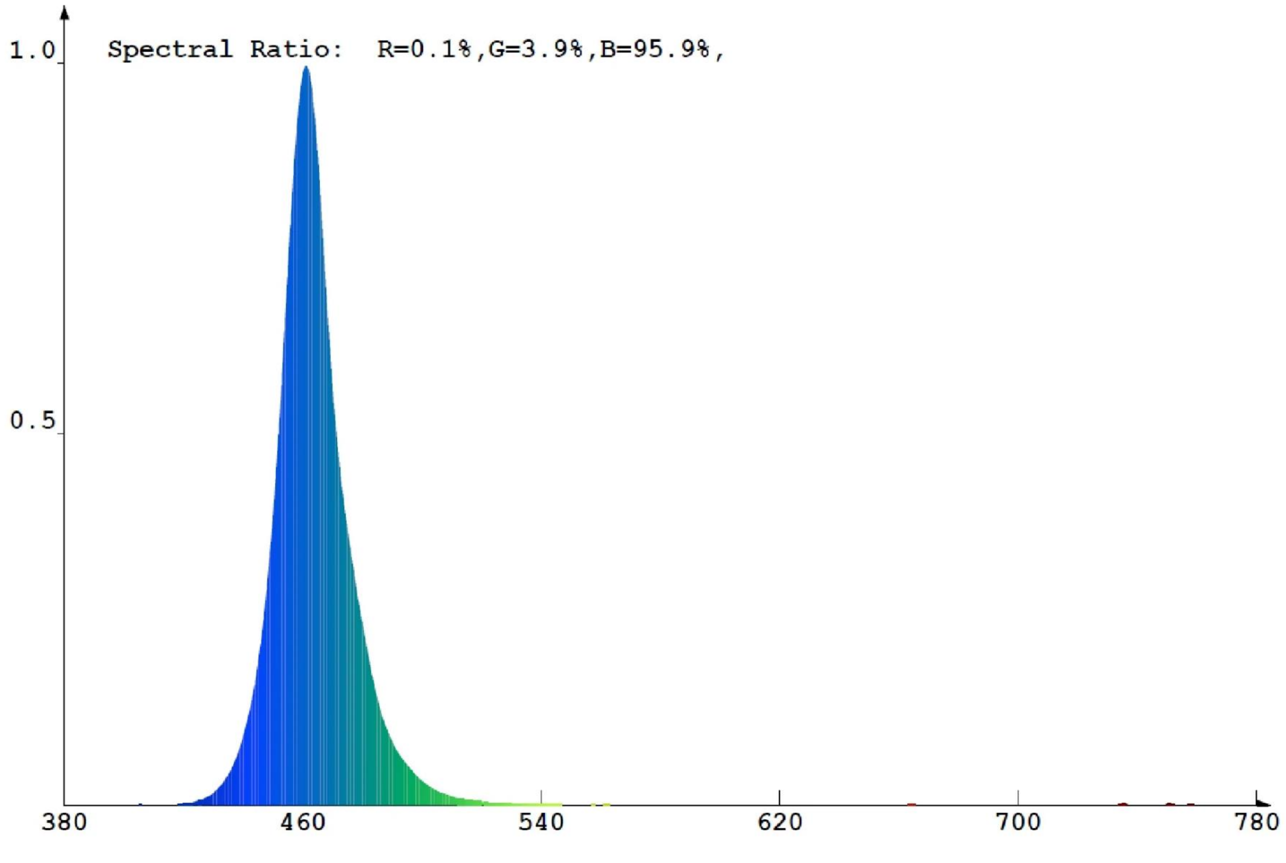

Figure S72. Spectral output from SimpleColor™ Blue LED Strip Lights (Waveform Lighting).

References:

1 S. Li and D. Ma, *J. Org. Chem.*, 2024, **89**, 6626–6630.

2 Shivani, A. Mishra, V. Kumar, P. Kaur and K. Singh, *Dyes Pigm.*, 2022, **200**, 110160.

3 D. Yousif, L. Vaghi, C. G. Daniliuc, R. Po, A. Papagni and F. Rizzo, *J. Org. Chem.*, 2023, **88**, 5285–5290.

4 Shivani, A. Mishra, P. Kaur and K. Singh, *ACS Omega*, 2022, **7**, 39045–39060.

5 P. Chen, H.-M. Wang, G.-J. Liu and S. X.-A. Zhang, *Org. Biomol. Chem.*, 2016, **14**, 4456–4463.

6 J. Shaya, F. Fontaine-Vive, B. Y. Michel and A. Burger, *Chem. Eur. J.*, 2016, **22**, 10627–10637.

7 S. Sasaki, Y. Niko, A. S. Klymchenko and G. Konishi, *Tetrahedron*, 2014, **70**, 7551–7559.

8 S. F. Musolino, M. Mahbod, R. Nazir, L. Bi, H. A. Graham, A. S. Milani and J. E. Wulff, *Polym. Chem.*, 2022, **13**, 3833–3839.

9 J. P. Marcolongo, J. Schmidt, N. Levin and L. D. Slep, *Phys. Chem. Chem. Phys.*, 2017, **19**, 21373–21381.

10 O. Filevich and R. Etchenique, *Photochem. Photobiol. Sci.*, 2013, **12**, 1565–1570.

11 R. Cabrera, M. Gabriel, L. C. Estrada and R. Etchenique, *Anal. Chem.*, 2019, **91**, 5968–5972.

12 Spartan 18. Wavefunction, Inc., Irvine CA, 2018.

13 Gaussian 16, Revision C.01, M. J. Frisch, G. W. Trucks, H. B. Schlegel, G. E. Scuseria, M. A. Robb, J. R. Cheeseman, G. Scalmani, V. Barone, G. A. Petersson, H. Nakatsuji, X. Li, M. Caricato, A. V. Marenich, J. Bloino, B. G. Janesko, R. Gomperts, B. Mennucci, H. P. Hratchian, J. V. Ortiz, A. F. Izmaylov, J. L. Sonnenberg, D. Williams-Young, F. Ding, F. Lipparini, F. Egidi, J. Goings, B. Peng, A. Petrone, T. Henderson, D. Ranasinghe, V. G. Zakrzewski, J. Gao, N. Rega, G. Zheng, W. Liang, M. Hada, M. Ehara, K. Toyota, R. Fukuda, J. Hasegawa, M. Ishida, T. Nakajima, Y. Honda, O. Kitao, H. Nakai, T. Vreven, K. Throssell, J. A. Montgomery, Jr., J. E. Peralta, F. Ogliaro, M. J. Bearpark, J. J. Heyd, E. N. Brothers, K. N. Kudin, V. N. Staroverov, T. A. Keith, R. Kobayashi, J. Normand, K. Raghavachari, A. P. Rendell, J. C. Burant, S. S. Iyengar, J. Tomasi, M. Cossi, J. M. Millam, M. Klene, C. Adamo, R. Cammi, J. W. Ochterski, R. L. Martin, K. Morokuma, O. Farkas, J. B. Foresman and D. J. Fox, Gaussian, Inc., Wallingford CT, 2016.
